# Supplementary material for: Autocatalytic degradation of the extremely potent greenhouse gas SF6 in basic alcoholic solution
Source: Nat Commun. 2025 Dec 6;17:465. doi: 10.1038/s41467-025-67158-w (PMC12800194; doi:10.1038/s41467-025-67158-w)
Supplement: Supplementary file 1 — Supplementary Information [file 41467_2025_67158_MOESM1_ESM.pdf]

Supplementary Information for

Autocatalytic degradation of the extremely potent greenhouse gas SF_6 in basic alcoholic solution

A. Sietmann[1], P. Heinzel[2], J. Gamper[1], D. Leitner[1], L. C. Pasqualini[1], F. R. S. Purtscher[1], H. Kopacka[1], T. S. Hofer[1], A. Zemann[2], F. Dielmann[1]*

[1]Institute of General, Inorganic and Theoretical Chemistry, Universität Innsbruck; 6020 Innsbruck, Austria

[2]Institute of Analytical Chemistry and Radiochemistry, Universität Innsbruck; 6020 Innsbruck, Austria

*Corresponding author: fabian.dielmann@uibk.ac.at

Table of Contents

1 Materials ... 4
2 Light sources ... 4
3 Analytical Methods ... 7
3.1 NMR Spectroscopy ... 7
3.2 Gas Chromatography Coupled to Mass Spectrometry (GC-MS) ... 7
3.3 Infrared (IR) Spectroscopy ... 8
3.4 X-ray Powder Diffraction (XRD) ... 8
3.5 Capillary Electrophoresis (CE) ... 9
3.6 pH Determination ... 10
3.7 Ultraviolet-visible (UV-vis) Spectroscopy ... 10
3.8 Electron Paramagnetic Resonance (EPR) Spectroscopy ... 10
3.9 Monitoring of the SF_6 Uptake: Pressure Control Unit (PCU) ... 11
4 Experimental Setup and Procedure of the Photochemical Reactions ... 14
4.1 General Procedure using Setup 1 (GP1) ... 14
4.2 General Procedure using Setup 2 (GP2) ... 14
4.3 General Procedure using Setup 3 (GP3) ... 15
4.4 General Procedure using Setup 4 (GP4) ... 15
5 Pre-Experiments and Screening of Reaction Conditions ... 17
5.1 Determination of the Solubility of SF_6 in Different Solvents ... 17
5.1.1 Quantitative Determination by ^{19}F NMR Spectroscopy ... 17
5.1.2 Qualitative Comparison Using the PCU ... 17
5.2 Base Screening using Isopropanol as Solvent ... 18
5.3 Wavelength Screening using the KOH/iPrOH System ... 19
5.4 Solvent Screening using KOH as Base ... 20
6 Photochemical Degradation of SF_6 in the KOH/iPrOH System ... 22
6.1 Analysis of the Reaction Products ... 22
6.1.1 Characterization of the Volatile Components ... 23
6.1.2 Characterization of the Solid Components ... 25
6.1.3 Determination of the Reaction Yield ... 28
6.2 Parameters Influencing the Reaction Rate ... 29
6.2.1 Molecular Oxygen ... 29
6.2.2 Pressure of the SF_6 Gas ... 30
6.2.3 Concentration of KOH ... 31
6.2.4 Irradiation Power ... 32
6.2.5 Irradiation time ... 34
6.2.6 Influence of phase mixing on the reaction rate ... 34
6.3 Scaleup of the KOH/iPrOH System ... 35
6.3.1 Irradiation Setup 3 ... 35
6.3.2 Irradiation Setup 4 ... 37
7 Photochemical Degradation of SF_6 in the biphasic KOH/H_2O/iPrOH System ... 39
7.1 Irradiation Setup 2 ... 39
7.2 Irradiation Setup 4 ... 40

8 Mechanistic Investigations 42
8.1 Influence of additives on the SF_6 degradation rate in the KOH/EtOH system 42
8.2 Influence of acetone on the SF_6 degradation rate in the biphasic KOH/H_2O/iPrOH system 43
8.2.1 Irradiation Setup 2 43
8.2.2 Irradiation setup 4 43
8.3 UV−vis absorption spectroscopy 45
8.4 EPR spectroscopy 48
8.5 Influence of diisopropyl sulfite 50
8.5.1 Hydrolysis of diisopropyl sulfite by excess of potassium hydroxide 50
8.5.2 Influence of diisopropyl sulfite on the SF_6 degradation rate in the KOH/iPrOH system51
8.6 Investigating the stability of acetone in the KOH/iPrOH and the KOH/H_2O/iPrOH system .. 52
8.7 The use of di-*tert*-butyl peroxide as radical initiator 53
8.8 Attempted generation of the dimethyl ketyl radical anion under anhydrous conditions and in-situ reaction with SF_6 54
8.9 Estimation of the quantum efficiency 54
8.10 Pulsed Irradiation to Assess the Contribution of the Dark Reaction 56
8.11 Influence of the isotope effect using the biphasic KOH/D_2O/iPrOH System 59
8.12 SF_6 degradation rate in quartz glass and borosilicate glass vessels 63
9 Degradation rates and energy efficiencies of the different setups 65
10 Overview of selected solution-based SF_6 degradation methods reported in the literature 66
11 Overview of selected gas phase photochemical SF_6 degradation methods reported in the literature 68
12 Computational Details 69
12.1 Methods 69
12.2 Results 69
12.2.1 Photo-induced formation of $SF_6^{\bullet-}$ 69
12.2.2 Dissociation pathways of $SF_6^{\bullet-}$ 72
12.2.3 Hydrogen atom abstraction from iPrOH by the radicals $F^{\bullet}$ and $SF_5^{\bullet}$ 72
12.2.4 Minimum Structures of $SF_6\cdots OH^-$ Encounter Complexes 72
13 Supplementary References 74

1 Materials

Sulfur hexafluoride 3.0 (99.9%) was generously donated by the company DILO GmbH. All other compounds were purchased from commercial sources and used as received if not stated otherwise. The water content of potassium hydroxide was determined by titration with an aqueous HCl solution (0.5 M) and bromothymol blue as pH indicator (KOH: 94.2%).

Alcohols (commercial source): methanol (HPLC grade 99.8%; FisherSci); ethanol (technical grade 96%, contains 1% isopropanol and 1% butanone, Donauchem); ethanol (absolute grade 99.5%; Acros Organics); *n*-propanol (p.a. 99.5%; Sigma Aldrich); isopropanol (technical grade 98%; VWR); isopropanol (HPLC grade 99.9%; Sigma Aldrich); *n*-butanol (99.9%; Sigma Aldrich); *sec*-butanol (ReagentPlus, 99%, Sigma Aldrich); 2-methyl-2-pentanol (99%; Sigma Aldrich); *n*-hexanol (98%; VWR); ethylene glycol (spectrophotometric grade 99%; Sigma Aldrich); 1-methoxypropan-2-ol (ReagentPlus 99.5%; Sigma Aldrich) and 2-propoxyethanol (98%; TCI).

Inorganic salts (commercial source): lithium hydroxide (98%; Sigma Aldrich); sodium hydroxide (98.6%; VWR); potassium hydroxide (>85%, pellets; Sigma Aldrich); calcium hydroxide (96%, Merck) potassium sulfate (99%, Sigma, Aldrich); potassium sulfite (90%; Sigma Aldrich); potassium thiosulfate (95%; Sigma Aldrich); potassium iodide (99.5%; Merck) and potassium fluoride (99%; VWR).

Chemicals used for the capillary electrophorese (commercial source): potassium iodide (99.5%; Merck); tris(hydroxymethyl)aminomethane (99.8%, Acros Organics); 2-(*N*-morpholino)-ethansulfonic acid hydrate (99.5%, Sigma Aldrich); arginine (99.5%, Sigma Aldrich); hexadimethrine bromide (95%, Sigma Aldrich).

Other chemicals used (commercial source): acetone (99.5%; Sigma Aldrich); 2-butanone (99%; Sigma Aldrich); pyrazine (98%, abcr); potassium triflate (98%; Sigma Aldrich); di-*tert*-butyl peroxide (98%; TCI).

2 Light sources

The photochemical experiments were carried out in quartz vessels with high optical transparency for wavelengths > 200 nm using the following light sources:

- **Light source for irradiation at 585 nm**: VCClilte LED Typ VAOL-SA1xAx-SA integrated into a customised irradiation device (Figure S1).
- **Light source for irradiation at 405 nm**: EvoluChem™ LED 405PF as part of the EvoluChem PhotoRedOx Box™ (Figure S2).
- **Light source for irradiation at 365 nm**: EvoluChem™ LED 365PF as part of the EvoluChem PhotoRedOx Box™ (Figure S2).
- **Light source for irradiation at 310 nm**: Seoulviosys LED Typ CUD1KFMA integrated into a customised irradiation device (Figure S1).
- **Light sources for irradiation at 280 nm**: Led-Tech XBT-3535-UV LED integrated into customised irradiation devices **LED-280-A** and **LED-280-B** (Figure S3). **LED-280-A** consists of an array of 5 LEDs powered by a Mean Well APC-25-500 LED driver leading to a total irradiation power of approximately 220 mW (input power: 17 W). **LED-280-B** consists of four arrays of 5 LEDs parallel connected and powered by a Mean Well HLG-150H-42A LED driver which results in a combined irradiation power of approximately 1.45 W (input power: 120 W).
- **Light source for irradiation at 250-550 nm**: Mercury-xenon lamp (MPDS-BASIC, nova®Light TXE 150) as part of a Peschl Photoreactor (Figure S4).

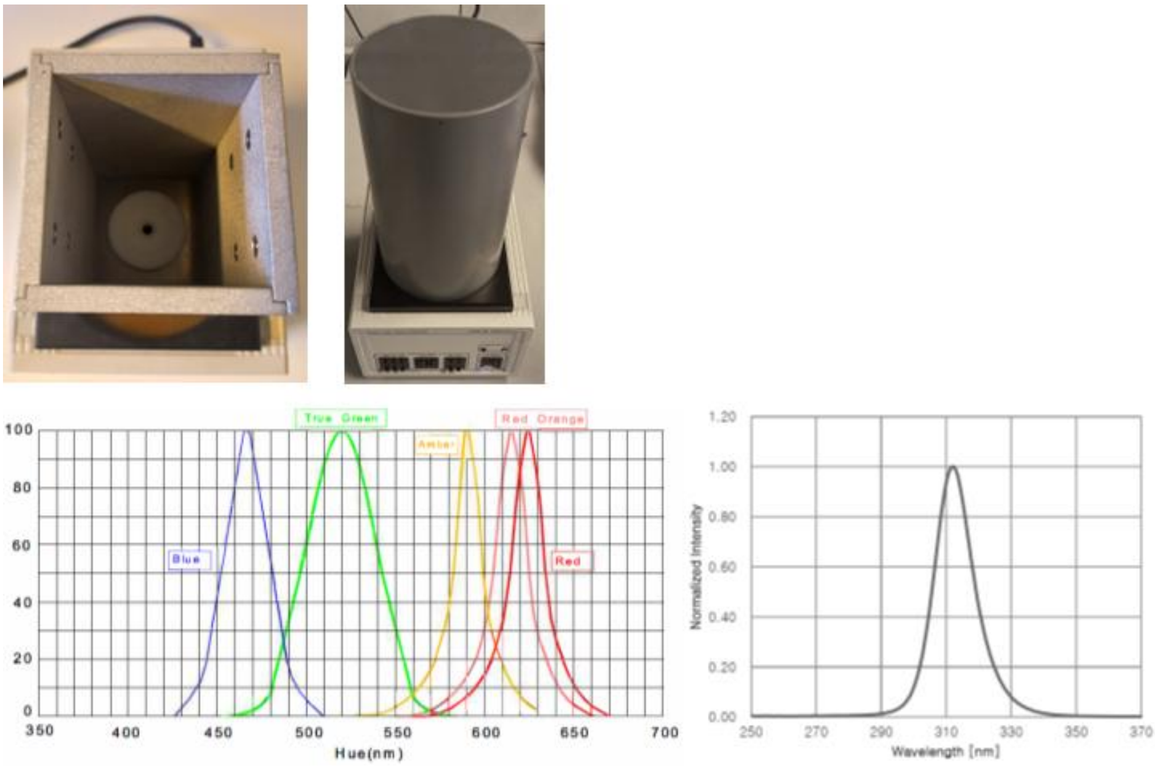

Figure S1: Picture of the custom build irradiation device with side on irradiation (top) and the emission spectra of the VCClilte LED Typ VAOL-SA1xAx-SA 585 nm (bottom left, amber) and the Seoulviosys LED Typ CUD1KFMA 310 nm (bottom right).[12]

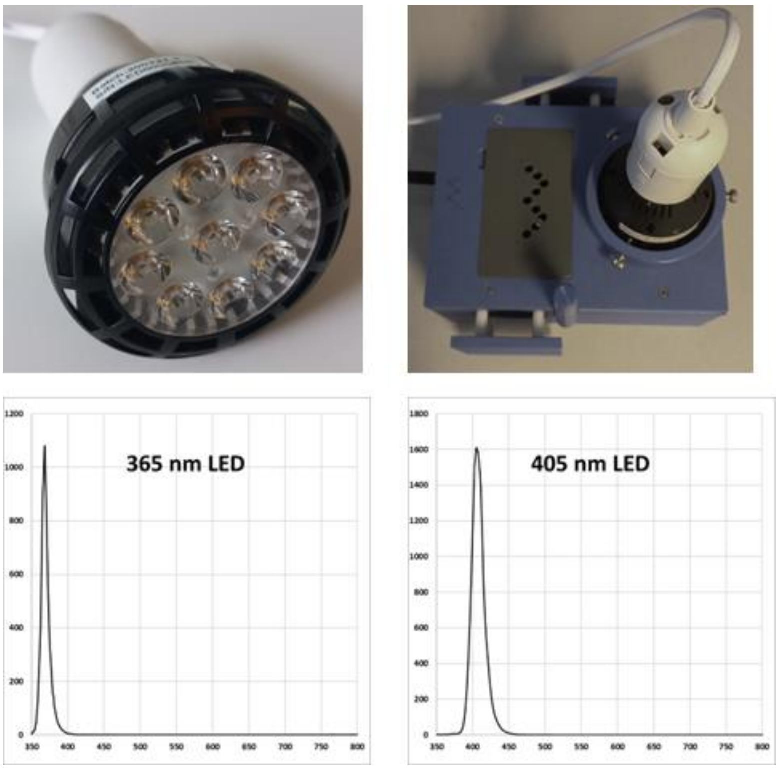

Figure S2: Picture of the EvoluChem PhotoRedOx Box™ with LED lamp (top) and the emission spectra of the EvoluChem™ LED 405 nm (bottomleft) and EvoluChem™ LED 365 nm (bottom right).[34]

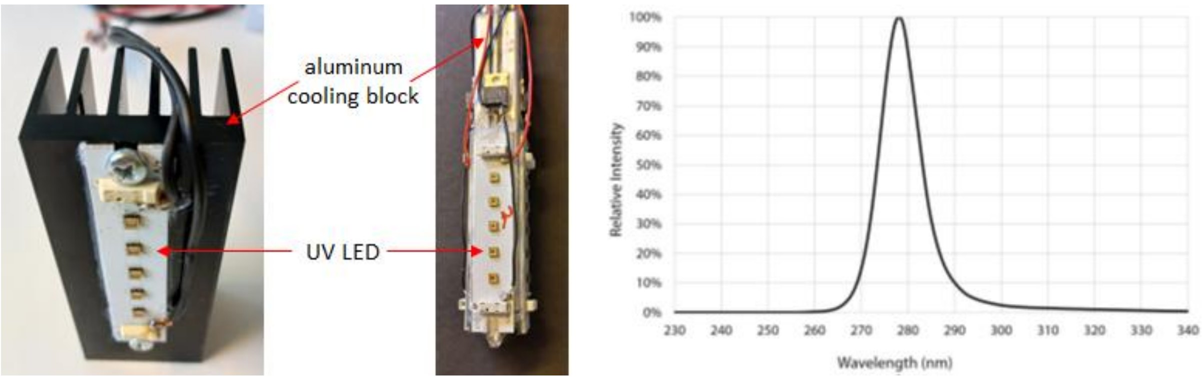

Figure S3: Picture of the customized light sources **LED-280-A** (left) and **LED-280-B** (middle) and the corresponding emission spectrum.[5]

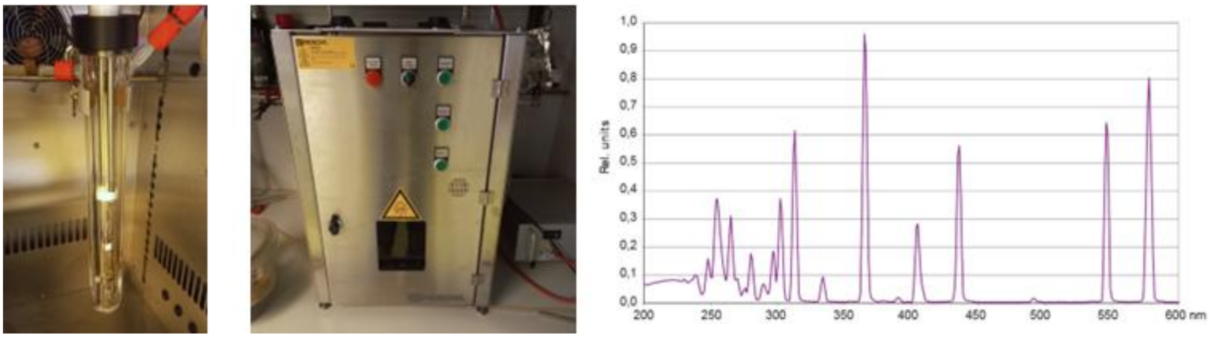

Figure S4: Picture of the mercury-xenon lamp (left) as part of the Peschl Photoreactor (middle) and the corresponding emission spectrum (right).[6]

3 Analytical Methods

3.1 NMR Spectroscopy

^{1}H, ^{13}C and ^{19}F NMR spectroscopy was performed on Bruker AVANCE I 400 or Bruker AVANCE III 400 spectrometers. Chemical shifts are given in parts per million (ppm) relative to $SiMe_4$ (^{1}H, ^{13}C) or $CFCl_3$ (^{19}F) and they were referenced to the residual solvent signals ($CDCl_3$: ^{1}H δ_H = 7.26 ppm, ^{13}C δ_C = 77.16 ppm). Chemical shifts (δ) are reported in ppm. NMR multiplicities are abbreviated as follows: s = singlet, d = doublet, sept = septet. For the determination of yields using NMR spectroscopy, quantitative ^{19}F NMR (^{19}F qNMR) and ^{1}H NMR experiments were performed with increased relaxations time (D1 = 25 seconds).

^{33}S NMR spectroscopy was performed on a Bruker DPX 300 spectrometer. To validate the detection limit of sulfate, sulfite and thiosulfate ions, aqueous solutions were prepared with different concentrations and analyzed by ^{33}S NMR spectroscopy (Table S1). The results show that sulfate ions can be detected at relatively low concentrations, while the resonance of thiosulfate and sulfite ions can only be detected in the case of concentrated solutions.

Table S1: ^{33}S NMR spectroscopy analysis of aqueous solutions of potassium sulfate, sulfite and thiosulfate.

Salt	Mass concentration (mg/mL)	Number of scans	Signal to noise	Chemical shift (ppm)
K_2SO_4	100	1472	6	0.4
	10	5920	2	
K_2SO_3	100	101377	2	0.1
		5920	-	
$K_2S_2O_3$	100	17888	4	33.9
		5920	-	

3.2 Gas Chromatography Coupled to Mass Spectrometry (GC-MS)

GC-MS was performed on a Shimadzu Nexis GC2030 gas chromatograph (GC) equipped with an autosampler, flame ionization detector (FID), triple quadrupole mass spectrometer (MS) with an EI ion source and a Rxi-5ms crossbond 5% diphenyl/ 95% polysiloxane column (30 m length, 250 µm diameter). The FID was operated at 300 °C with a gas flow consisting of He (24 ml/min), H_2 (32 ml/min) and air (200 ml/min) and the MS was operated at 200 °C (ion source) and 250 °C (interface). For the analysis one drop of the volatile components was added to ethyl acetate (1.5 ml) to analyze high boiling molecules or to decalin (1.5 ml) for low boiling molecules. The obtained solutions were filtered over silica. Afterwards the following standard GC-MS protocol was used for each measurement:

GC: 40 °C for 5 min → gradient increase from 40 °C – 300 °C (15 °C in 1 min) → 300 °C for 10 min.

FID: operated from 0 min → 30 min.

MS: operated from 4 min → 30 min (ethyl acetate) and 0 min → 10 min (decalin).

Using the standard GC-MS protocol, isopropanol, acetone, diisopropyl sulfite and a 1% solution of acetone in isopropanol were analyzed (Table S2). Isopropanol and acetone are not separated via gas chromatography under the applied conditions and are detected with the same retention time of 2.2 min using decalin. As confirmed by the analysis of the 1% solution of acetone in isopropanol, the GC-MS is not able to differentiate between acetone and isopropanol. Diisopropyl sulfite can be detected when ethyl acetate is used as an eluent. These results show that the acetone content of isopropanol solutions cannot be determined by GC-MS, but other volatile substances including diisopropyl sulfite can be identified.

Table S2: Results of the GC-MS measurements of isopropanol, acetone, diisopropyl sulfite and a 1% solution of acetone in isopropanol using the standard GC-MS protocol. (- = not detectible by the method)

Molecule	Solvent	Retention time of signal (min)
Isopropanol	Ethyl acetate	-
	Decalin	2.2
Acetone	Ethyl acetate	-
	Decalin	2.2
solution of 1% acetone in isopropanol	Ethyl acetate	-
	Decalin	2.2
Diisopropyl sulfite	Ethyl acetate	10.3
	Decalin	-

3.3 Infrared (IR) Spectroscopy

IR spectroscopy was carried out on a *Bruker ALPHA II* FT-IR spectrometer with Platin ATR device. To validate the analytical method, solid samples of the potassium salts of fluoride, sulfate, sulfite and thiosulfate were analyzed using the ATR device (Figure S5). Potassium fluoride shows no resonances in the range of 1300 – 400 cm^{-1}. However, resonances that are distinguishable appear for sulfate (1101 and 613 cm^{-1}), sulfite (949, 619 and 477 cm^{-1}), and thiosulfate (1122, 993, 662, 545 and 530 cm^{-1}) indicating that this method can be used for the identification of sulfur-containing anions in solid samples.

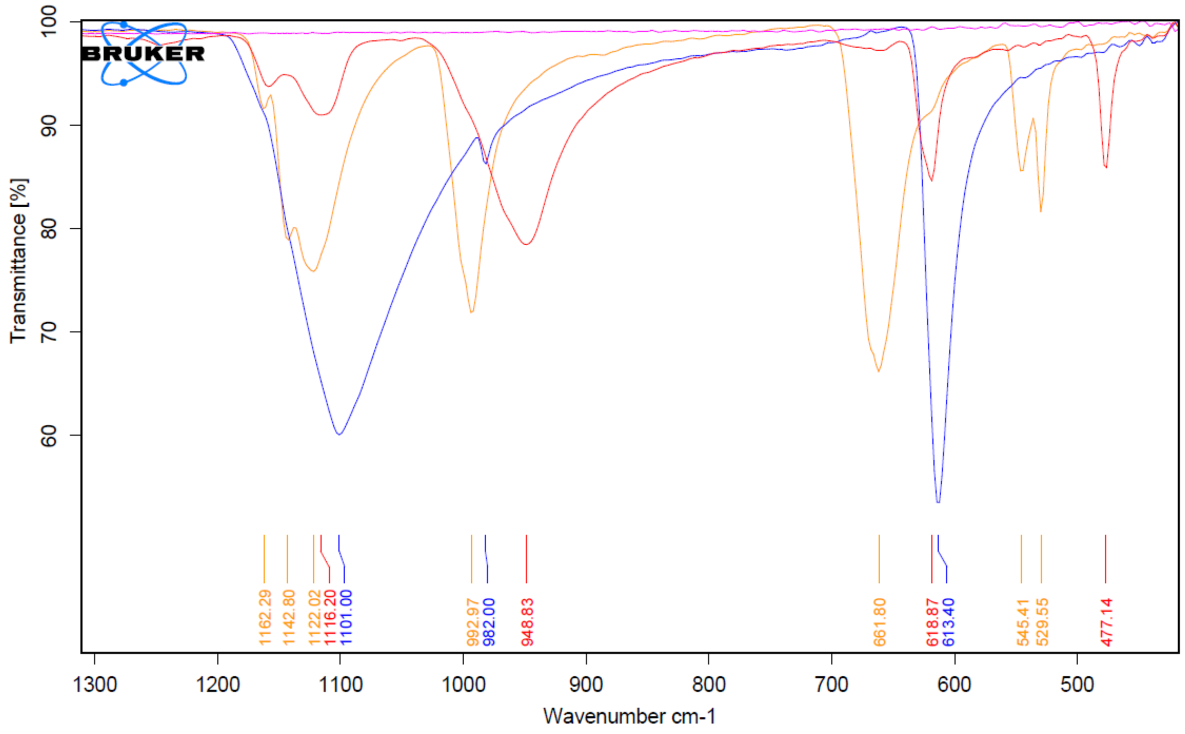

Figure S5: Overlayed IR spectra of potassium fluoride (pink), potassium sulfate (blue), potassium sulfite (red) and potassium thiosulfate (orange) in the range of 1300 – 400 cm^{-1}.

3.4 X-ray Powder Diffraction (XRD)

Powder diffractometry was performed on a STOE Stadi P powder diffractometer. Measurements were performed in transmission geometry with Ge(111)-monochromatized MoK-L_3 radiation

(λ = 0.7093 Å) within a range of 2ϑ = 2–70°, a step size of 0.015° and a Mythen 1 K detector. The *Topas 4.2* software was used for the Rietveld refinement.[7]

3.5 Capillary Electrophoresis (CE)

Capillary electrophoresis was performed on a 1600 CE-System from *Agilent* with a capacitively coupled contactless conductivity detector from *Innovative Sensor Technologies GmbH* and a capillary (80 cm length, 50 µm inner diameter) from *Polymicro Technologies*. The evaluation was done using the software packages *Agilent CE ChemStation*, *OriginPro* and *MSExcel*. Before measurements the capillary was dynamically coated with hexadimethrine hydroxide in four steps. The capillary was washed with NaOH (0.1 M) for 10 minutes, milli-Q water for 5 minutes, hexadimethrine hydroxide for 20 minutes and the background electrolyte for 3 minutes. The optimised background electrolyte consists of an aqueous solution of 2-(*N*-morpholino)ethanesulfonic acid (13.1 mM), tris(hydroxymethyl)-aminomethane (46.5 mM) and arginine (56.4 mM). All measurements were conducted at 20 °C with a negatively polarisation at the inlet side (30 kV). A calibration was done using aqueous solutions of a salt mixture consisting of sodium fluoride, sodium sulfite, lithium sufate and sodium thiosulfate with different anion concentrations (1, 5, 10 and 15 mg/L) and potassium iodide (8 mg/L) as internal standard. The obtained calibration line and respective linear equation were used for the quantification of the anions.

The following standard CE protocol was used for each measurement:

→ the capillary was washed with the background electrolyte for 3 minutes.

→ the sample solution was pressure injected for 10 seconds (30 mbar).

→ the capillary was washed with milli-Q water for 10 minutes.

To confirm the applicability of this method potassium salts of fluoride (entry 1), sulfate (entry 2), sulfite (entry 3), thiosulfate (entry 4) and salt mixtures consisting of potassium fluoride/sulfite (entry 5), fluoride/thiosulfate (entry 6) and fluoride/sulfite/thiosulfate (entry 7) were analyzed (Table S3). For each sample, a solution of the salt or the salt mixtures with an anion concentration of 15 mg/L was prepared. Each sample solution was measured two times following the standard CE protocol. For the potassium salts of fluoride, sulfate and thiosulfate and salt mixtures containing these salts, the expected anions fluoride, sulfate and thiosulfate were detected.

However, the method has two major limitations:

1) The experimentally measured concentrations differ significantly from the theoretical values (e.g. Entry 1: $c_{exp}(F^-)$ = 12.9 to $c_{theo}(F^-)$ = 15 mg/L). In addition, standard deviations of up to 1.9 mg/L were observed (Table S3, right column).
2) Sulfite is partly converted into sulfate during the CE measurements. This was confirmed by analyzing sulfite salts by CE, and additionally by ^{33}S NMR spectroscopy (see chapter 3.1) and IR spectroscopy (see chapter 3.3). CE analysis of sulfite salts and sulfite salt mixtures (Entry 3, 5, 7) indicated the presence of sulfate in large quantities based on the initial amount of sulfite (Entry 3: 10%, Entry 5: 23%, Entry 7: 68%). By contrast, no sulfate was detected upon analysis of potassium sulfite by the other two methods.

Considering these experimental observations the CE analysis can only be used as a qualitative method for the identification of the fluoride, sulfite and thiosulfate anions.

Table S3: Results of the CE analysis of different potassium salts and salt mixtures.

Entry	Salt	c_{theo} *of anion* (mg/L)	Anions detected	c_{exp} *of anion* (mg/L)		Average (mg/L)	Standard deviation (mg/L)
				Run 1	Run 2		
1.	KF	15	F^-	12.92	12.80	12.86	0.08

2.	K_2SO_4	15	SO_4^{2-}	13.53	13.63	13.58	0.07
3.	K_2SO_3	15	SO_3^{2-}	14.7	15.0	14.9	0.2
			SO_4^{2-}	1.8	1.3	1.6	0.4
4.	$K_2S_2O_3$	15	$S_2O_3^{2-}$	13.17	13.06	13.11	0.08
5.	KF/K_2SO_3	15	F^-	11.8	12.0	11.9	0.1
			SO_3^{2-}	9.6	9.2	9.4	0.2
			SO_4^{2-}	2.6	2.9	2.8	0.2
6.	$KF/K_2S_2O_3$	15	F^-	19.5	16.8	18.1	1.9
			$S_2O_3^{2-}$	16.9	14.5	15.7	1.7
7.	$KF/K_2SO_3/K_2S_2O_3$	15	F^-	17.6	16.2	16.9	1.0
			SO_3^{2-}	6.8	4.5	5.6	1.6
			SO_4^{2-}	11.4	12.4	11.9	0.7
			$S_2O_3^{2-}$	13.4	12.2	12.8	0.8

3.6 pH Determination

pH determination was carried out using an *inoLab® pH 7110* meter. Prior to measurements, a two-point calibration was done using two technical buffers (buffer solution pH 7.00 and buffer solution pH 4.00 from Carl Roth).

3.7 Ultraviolet-visible (UV-vis) Spectroscopy

UV-vis absorption spectroscopy was performed on a *PerkinElmer* LAMBDA XLS+ spectrophotometer using standard quartz UV-vis cuvettes (d = 1 cm). The absorption was measured from 200 – 900 nm.

3.8 Electron Paramagnetic Resonance (EPR) Spectroscopy

EPR spectroscopy was performed on a *Bruker* Magnettech ESR5000 X-band spectrometer equipped with a temperature control unit and a high intensity mercury-xenon lamp (Hamamatsu 200W Mercury Xenon 365nm wide band L9566-06A) with a spectral distribution of 240 to 550 nm (Figure S6). Measurements were carried out in 3 mm o.d. fused silica tubes.

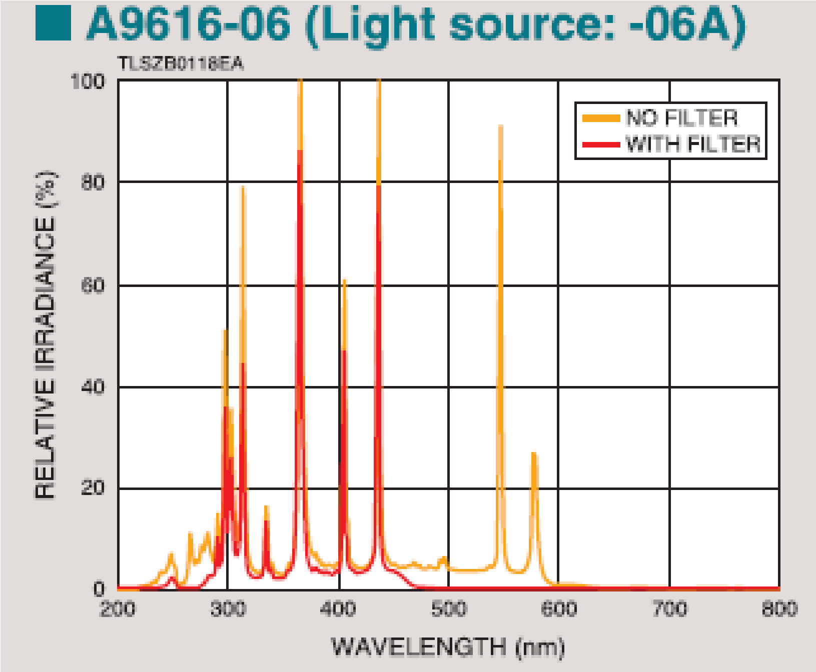

Figure S6: Emission spectra of the mercury-xenon lamp of the Bruker Magnettech ESR5000 (240-550 nm; no filter).[8]

3.9 Monitoring of the SF_6 Uptake: Pressure Control Unit (PCU)

Monitoring the SF_6 uptake was accomplished by a self-built electrical pressure control unit (PCU) that is shown in Figure S7. Data readout and pressure control is done using an Arduino Nano E/A-Board with an ATmega328 microcontroller. The pressure regulation unit consists of an inlet tube with 3 bar SF_6 pressure from a storage cylinder, which is attached to a magnetic valve. The outlet of the magnetic valve is connected to a piezo-resistive silicon pressure sensor and to the reaction vessel using a T-piece adapter. When the pressure in the reaction vessel drops below a given threshold, the magnetic valve opens for a fixed time interval of 17 ms (Irradiation Setup 2 and 3) or 60 ms (Irradiation Setup 4). The number of cycles (opening and closing of valve) thus corresponds to a defined SF_6 uptake by the reaction mixture. A calibration experiment was performed according to **GP2** (see Chapter 4.2) using 1.0 g KOH and 15 mL iPrOH. Analysis of the reaction products (see Chapter 6.1 for details) reveals an overall decomposition of 2.3 mmol SF_6 which corresponds to a uptake of 0.05 mmol SF_6 per cycle, at a given SF_6 storage cylinder pressure of 3 bar and reaction vessel pressure of 2 bar (Equation 1).

$$n(\mathrm{SF_6\ per\ cycle}) = \frac{n(\mathrm{SF_6\ uptake})}{\mathrm{valve\ cycles\ by\ PCU}} \qquad (1.)$$

$$n(\mathrm{SF_6\ per\ cycle}) = \frac{2.3\ \mathrm{mmol}}{45} = 0.05\ \mathrm{mmol}$$

The generated data (saved as a .csv file) includes the time, the number of valve cycles by the PCU, and the pressure in the reaction vessel. The data was imported to *OriginPro* and the valve cycles by PCU were plotted against the reaction time leading to the SF_6 uptake time plot shown in Figure S8.

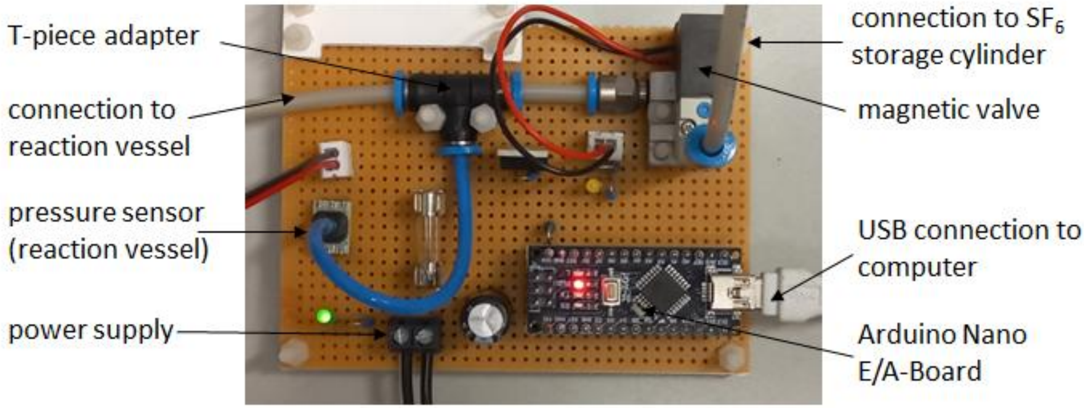

Figure S7: The self-built pressure control unit (PCU) used for monitoring of the SF_6 uptake.

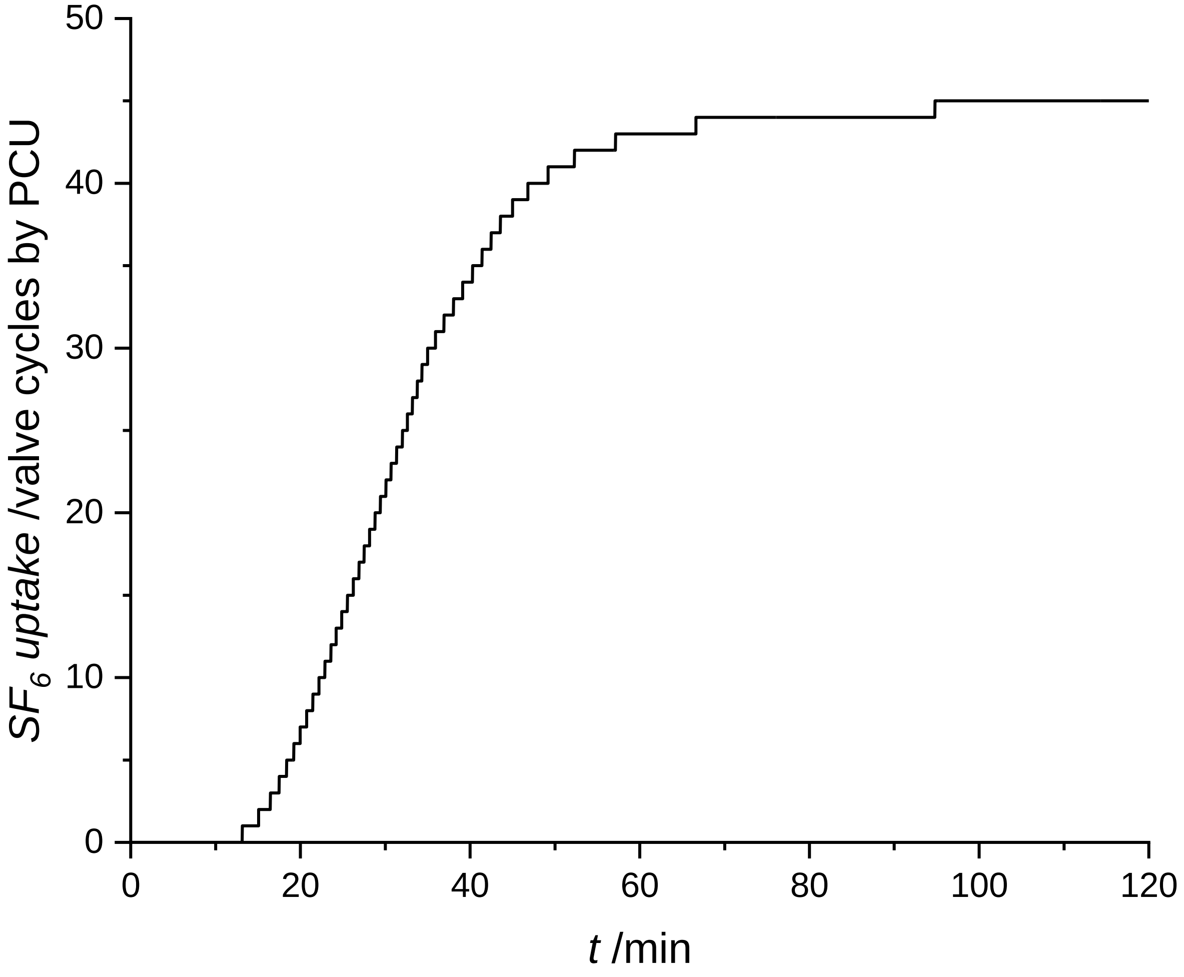

Figure S8: SF_6 uptake time plot obtained by monitoring the KOH/iPrOH system (**GP2**) with the pressure control unit (PCU).

Validation of the PCU. To verify that the measured switching cycles correlate with the uptake of SF_6, pure iPrOH and a mixture of iPrOH/KOH (following **GP2** in Chapter 4.2) were each irradiated with light at 280 nm for 30 min and the pressure change in the reaction vessel was monitored (Figure S9). The graph in Figure S9 shows that in the absence of potassium hydroxide, the pressure in the quartz tube rises slowly. This increase in pressure is attributed to the warming of the solvent by the irradiation, which is accompanied by a lower solubility of SF_6 in isopropanol. In the presence of potassium hydroxide, a similar behaviour is observed at the beginning of the reaction, but after around 7 minutes the pressure decreases due to SF_6 degradation. The pressure profile shows that the warming of the reaction mixture during irradiation increases the SF_6 gas pressure to a certain extent, but this is not the cause of the observed delayed switching cycles of the PCU.

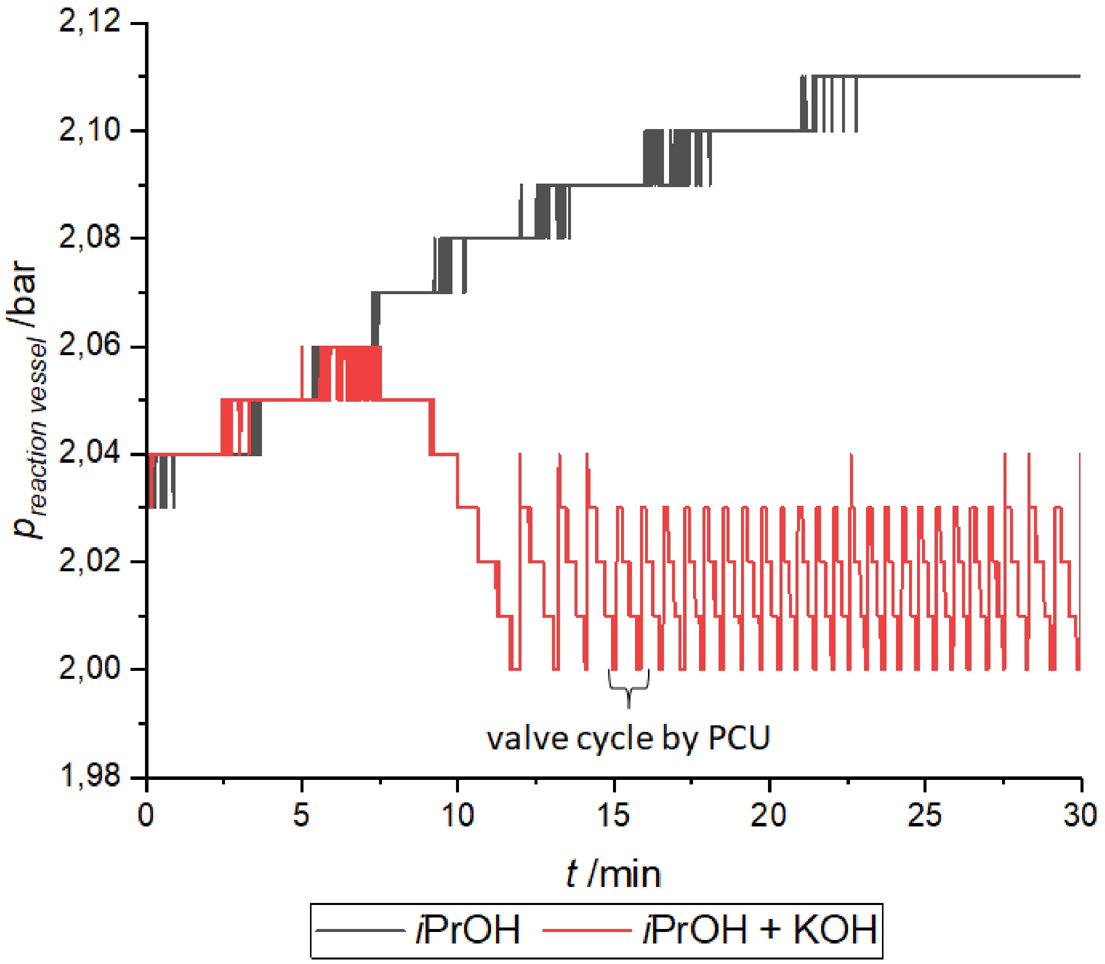

Figure S9: SF_6 pressure profile in the reaction vessel obtained by irradiation of iPrOH (black) and iPrOH/KOH (red) for 30 minutes with light at 280 nm. The threshold for the switching cycles was set at 2.0 bar.

4 Experimental Setup and Procedure of the Photochemical Reactions

The photochemical reactions were carried out with different setups depending on the reaction scale in order to minimize the release of SF_6 and to be able to monitor the progress of the reaction.

- **Irradiation Setup 1**: A liquid sample of **1 mL** in a quartz glass NMR tube (diameter 5 mm) was irradiated from the side using LED-280-A cooled with a fan (Figure S10).
- **Irradiation Setup 2**: A liquid sample of **15 mL** in a quartz tube (diameter 17 mm) was irradiated from the side using LED-280-A cooled with a fan (Figure S11).
- **Irradiation Setup 3**: A liquid sample of **80 mL** in borosilicate glass tube (diameter 42 mm) equipped with a quartz glass sight glass at the top (diameter 40 mm) was irradiated from the top using LED-280-A cooled with a fan (Figure S12).
- **Irradiation Setup 4**: A liquid sample of **850 mL** in a borosilicate round bottom flask (1 L) was irradiated via an internal quartz tube containing the LED-280-B that is equipped with a water-cooling system (Figure S13).

4.1 General Procedure using Setup 1 (GP1)

A quartz glass NMR tube was charged with the base (0.46 mmol) dissolved in a solvent (1 ml). The solution was pressurised with 3 bar SF_6 and the tube was sealed with a PTFE valve. The gas-tight tube was shaken vigorously and then irradiated with light of a given wavelength for 1 hour. The resulting reaction mixture was transferred into a vial. Evaporation of the volatiles under reduced pressure gave a solid residue consisting of a mixture of inorganic salts (e.g. KF, K_2SO_3). The residue was dissolved in water and the content of KF was determined by quantitative ^{19}F NMR spectroscopy using potassium triflate (10.0 mg, 0.05 mmol) as internal standard. Assignment of the ^{19}F NMR resonances: $\delta = -78.6$ (OTf^-), -119.1 (F^-) ppm.

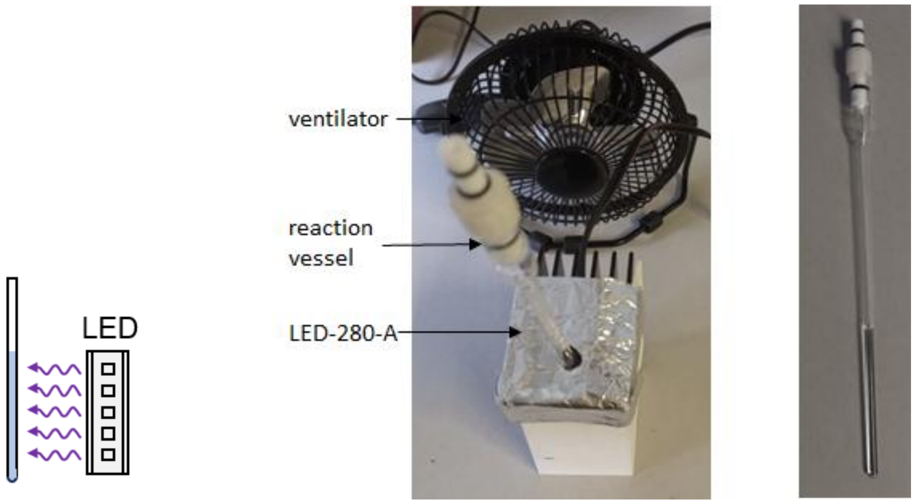

Figure S10: Schematic illustration (left) and picture (middle) of Irradiation Setup 1 and the 1 mL quartz glass NMR tube (right).

4.2 General Procedure using Setup 2 (GP2)

A quartz tube was charged with a solution of potassium hydroxide in a solvent (15 ml). The solution was degassed by four freeze-pump-thaw cycles and the reaction tube was pressurized with SF_6. After the solution was saturated with SF_6 gas (see Figure S14), the reaction mixture was irradiated for 2 h with light at 280 nm using LED-280-A and the uptake of SF_6 was monitored using the PCU (see Chapter 3.9). The resulting reaction mixture was transferred into a 100 mL round bottom flask using small amounts of water to ensure complete transfer. The volatiles were evaporated under reduced pressure to afford a mixture of inorganic salts (e.g. KF, K_2SO_3). The residue was dissolved in water until a volume of 5 mL was obtained. An aliquot of 1 ml of this aqueous solution was analysed by

quantitative ^{19}F NMR spectroscopy using potassium triflate (10.0 mg, 0.053 mmol) as internal standard. Assignment of the ^{19}F NMR resonances: δ = −78.6 (OTf^-), −119.1 (F^-) ppm.

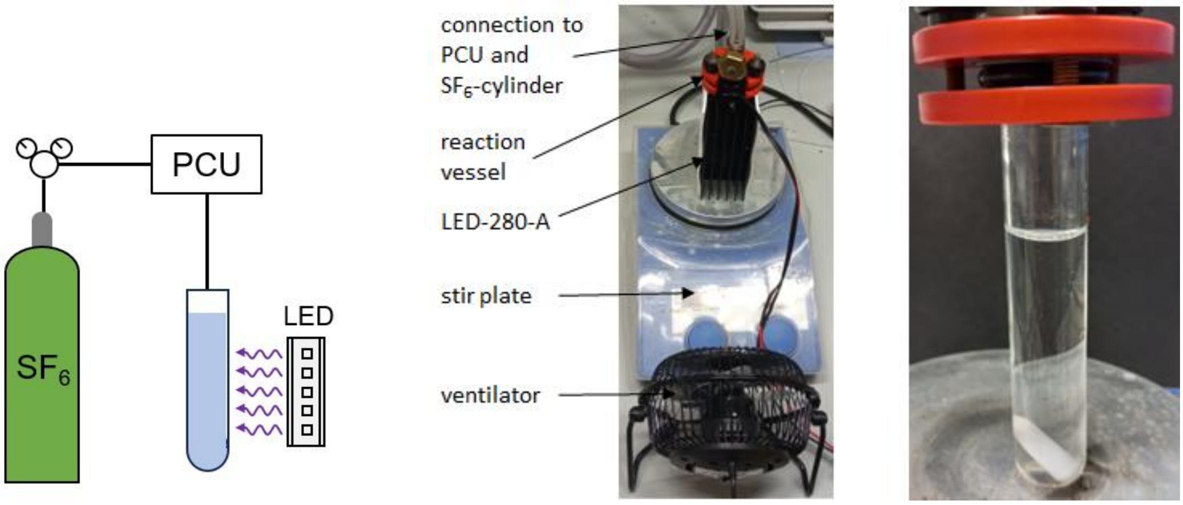

Figure S11: Schematic illustration (left) and picture (middle) of Irradiation Setup 2 and the 15 ml quartz tube (right).

4.3 General Procedure using Setup 3 (GP3)

A borosilicate glass tube equipped with a quartz sight glass at the top was charged with a solution of potassium hydroxide in a solvent (80 ml). The reaction vessel was pressurized with SF_6 (1 bar). After saturation with SF_6 gas was indicated by the PCU, the reaction mixture was irradiated with light at 280 nm using LED-280-A for the given time period. The uptake of SF_6 was monitored using the PCU (see Chapter 3.9). The resulting reaction mixture was transferred into a 250 mL round bottom flask using small amounts of water to ensure complete transfer. The volatiles were evaporated under reduced pressure to afford a mixture of inorganic salts (e.g. KF, K_2SO_3). The residue was dissolved in water until a volume of 40 mL was obtained. An aliquot of 1 ml of this aqueous solution was analysed by quantitative ^{19}F NMR spectroscopy using potassium triflate (10.0 mg, 0.053 mmol) as internal standard. Assignment of the ^{19}F NMR resonances: δ = −78.6 (OTf^-), −119.1 (F^-) ppm.

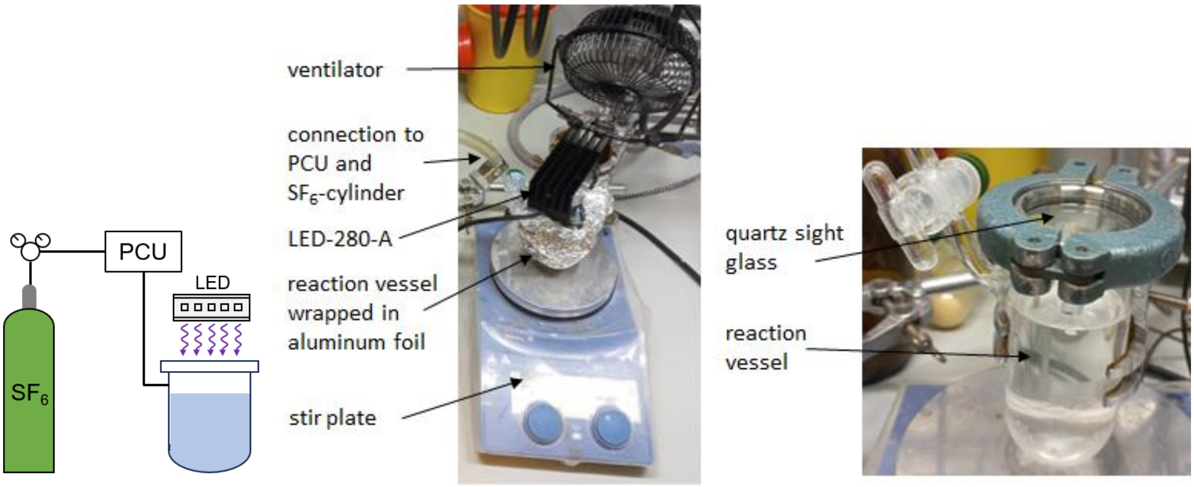

Figure S12: Schematic illustration (left) and picture (middle) of Irradiation Setup 3 and the 80 mL borosilicate tube with quartz glass sight glass (right).

4.4 General Procedure using Setup 4 (GP4)

A borosilicate round bottom flask with an internal quartz tube (diameter 34 mm) was charged with a solution of potassium hydroxide (60 g) in a solvent (850 ml). The reaction vessel was pressurized with SF_6 (2 bar). After saturation with SF_6 gas was indicated by the PCU, the reaction mixture was

irradiated with light at 280 nm using LED-280-B for the given time period. The uptake of SF_6 was monitored using the PCU (see Chapter 3.9). The organic and inorganic compounds were separated and were analyzed by several methods.

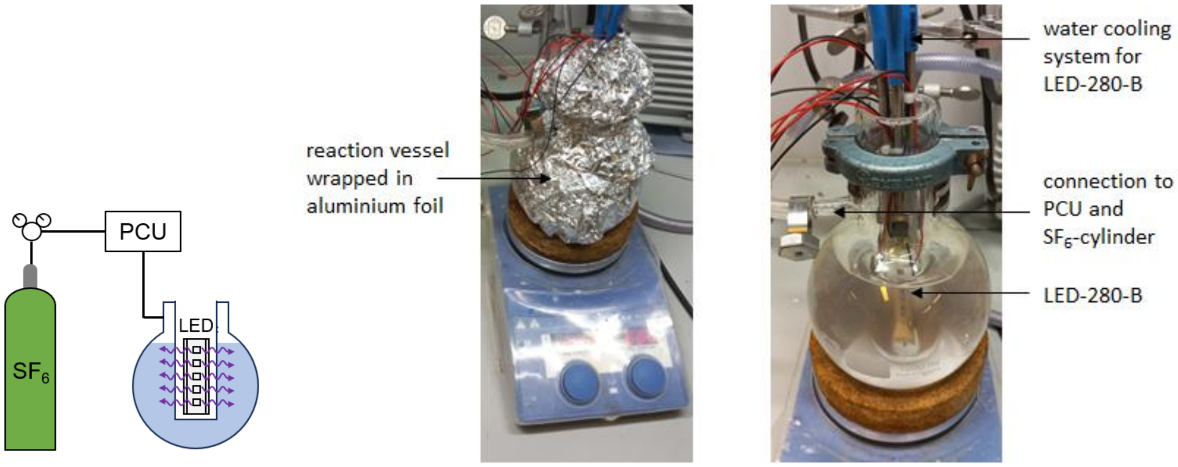

Figure S13: Schematic illustration (left) and picture (middle) of Irradiation Setup 4 and the 850 mL borosilicate round bottom flask with internal quartz tube (right).

5 Pre-Experiments and Screening of Reaction Conditions

5.1 Determination of the Solubility of SF_6 in Different Solvents

5.1.1 Quantitative Determination by ^{19}F NMR Spectroscopy

A sealed glass capillary containing a 1:2 mixture of 1,2-difluorobenzene and toluene was used as internal reference. As a reference for the ^{19}F content of the capillary (Equation 2), a ^{19}F qNMR spectrum was recorded of a solution of potassium triflate (40.0 mg, 0.213 mmol) in water (1 ml) in an NMR tube containing the capillary. The same capillary and NMR tube was then used for the quantification of the SF_6 solubility in different solvents. The NMR tube containing the capillary was charged with a solvent (1 ml). The solvent was degassed by 3 freeze-pump-thaw cycles and the NMR tube was pressurized with SF_6 (2 bar). Analysis of the sample by ^{19}F qNMR spectroscopy gave the concentration of dissolved SF_6 in the respective solvent at 2 bar SF_6 pressure according to Equation 3 (Table S4).

$$c(F_{capillary}) = c(F_{KOTf}) \cdot 3 \cdot \frac{Peak\ (F_{capillary})}{Peak\ (F_{KOTf})} \tag{2.}$$

$$c(F_{capillary}) = 213\ \frac{\text{mmol}}{\text{L}} \cdot 3 \cdot \frac{100}{91.13} = 700 \frac{\text{mmol}}{\text{L}}$$

$$c_{solvent}(SF_6) = c(F_{capillary}) \cdot \frac{Peak\ (SF_6)}{Peak\ (F_{capillary})} \cdot \frac{1}{6} \tag{3.}$$

$$c_{isopropanol}(SF_6) = 700 \frac{\text{mmol}}{\text{L}} \cdot \frac{88.57}{100} \cdot \frac{1}{6} = 103 \frac{\text{mmol}}{\text{L}}$$

Table S4: Solubility of SF_6 in different solvents at 2 bar SF_6 pressure and 25 °C.

Solvent	$c(SF_6)$ (mmol/L)
Water	1
Toluene	62
Ethanol (absolute)	68
Tetrahydrofuran	82
Isopropanol (HPLC grade) + KOH (30 mg)	82
Isopropanol (HPLC grade)	103
n-Hexane	111

5.1.2 Qualitative Comparison Using the PCU

A quartz tube (diameter 17 mm) containing a solvent (15 ml) under air atmosphere (1 atm.) was pressurized with 2 bar SF_6 and the SF_6 uptake was monitored using the PCU for 2 hours (see Chapter 3.9). The pressure increase from 1 bar to 2 bar is initially equalised by approx. 26 rapid switching cycles of the PCU. The solvent then absorbs the gas atmosphere in the sealed vessel until it is saturated (Table S5, Figure S14). In the case of isopropanol, this takes about 80 minutes. The addition of potassium hydroxide (1.0 g, 16.8 mmol) to iPrOH or EtOH increases the polarity of the solution and thus significantly decreases the solubility of SF_6.

Table S5: Qualitative comparison of the SF_6 uptake by different solvents (15 ml) at 21 °C when starting at 1 bar atmospheric pressure and pressurizing the vessel with 2 bar SF_6.

Solvent	Valve cycles by PCU
Water	29
Ethanol (abs.)	50
Ethanol (abs.) + KOH	45

Isopropanol (HPLC grade)	55
Isopropanol (HPLC grade) + KOH	51
n-Butanol	48

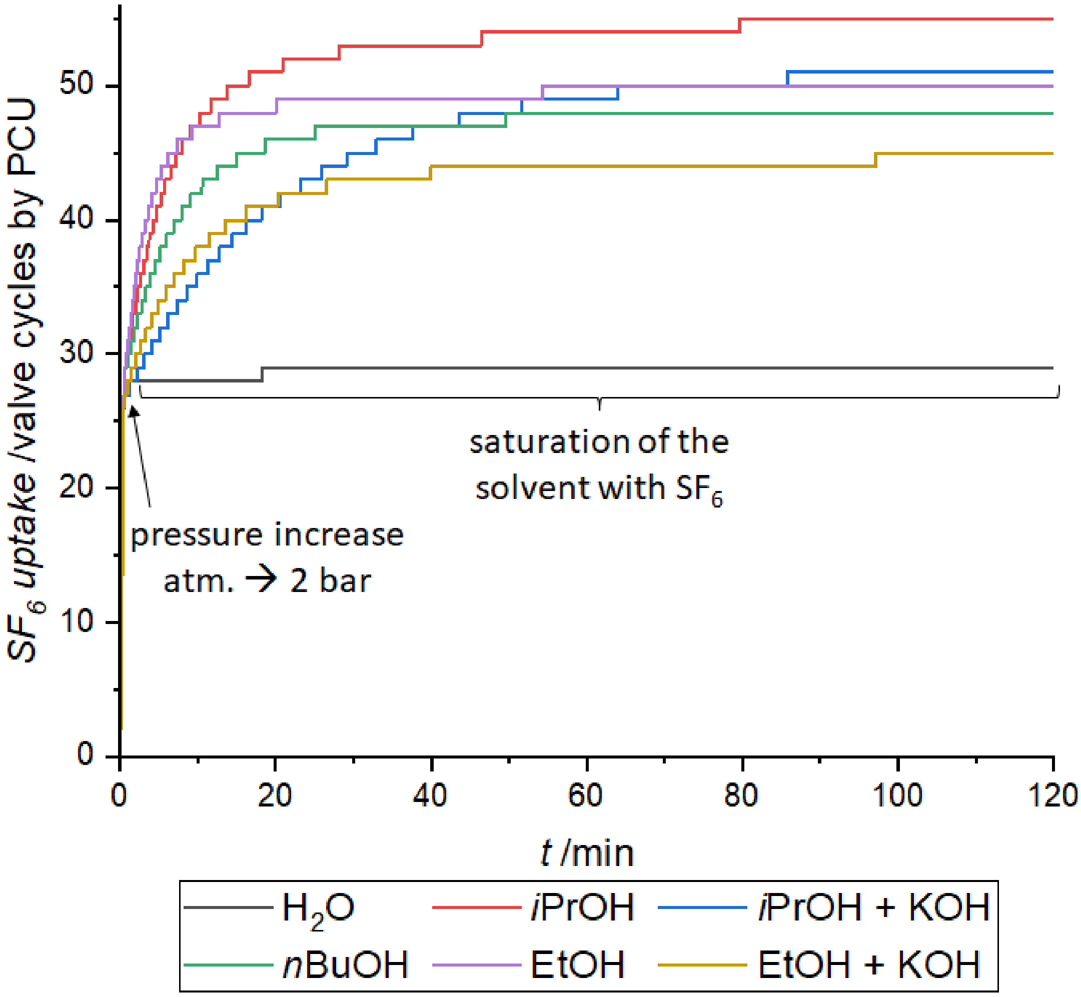

Figure S14: SF_6 uptake time plot obtained by the PCU showing the SF_6 uptake by different solvents (15 ml) at 21 °C when starting at 1 bar atmospheric pressure and pressurizing the vessel with 2 bar SF_6.

5.2 Base Screening using Isopropanol as Solvent

Screening of suitable bases was carried out using isopropanol (HPLC grade) as the solvent following **GP1** (base: 0.46 mmol; isopropanol (HPLC grade): 1 ml, SF_6 pressure: 3 bar, irradiation time: 1 h; see Chapter 4.1). Alkali metal hydroxides LiOH (11.1 mg, 0.46 mmol), NaOH (18.6 mg, 0.46 mmol), KOH (30.0 mg, 0.46 mmol) and $Ca(OH)_2$ (34.1 mg, 0.46 mmol) were employed. In the case of LiOH, NaOH and $Ca(OH)_2$ a suspension was irradiated due to the low solubility of the hydroxide salts in isopropanol. The resulting reaction mixtures were analysed by ^{19}F qNMR spectroscopy as described in **GP1** (Table S6). In the case of $Ca(OH)_2$, a suspension was obtained due to the low solubility in water. This suspension was acidified with hydrochloric acid (0.2 ml, 12 mol/L) to generate a solution for analysis by ^{19}F NMR spectroscopy.

Table S6: Estimated yields based on fluoride formation (determined by ^{19}F qNMR spectroscopy) assuming MOH conversion according to the equation: 8 MOH + SF_6 + iPrOH → 6 MF + M_2SO_3 + acetone + 5 H_2O.

Base	Yield (%)
LiOH*	4
NaOH*	42

KOH	>99
$Ca(OH)_2$*	0

*Irradiation of a suspension

5.3 Wavelength Screening using the KOH/iPrOH System

Screening of the irradiation wavelength was carried out using the KOH/iPrOH system following **GP1** (KOH: 30.0 mg, 0.46 mmol; isopropanol (HPLC grade): 1 ml, SF_6 pressure: 3 bar, irradiation time: 1 h; see Chapter 4.1). The quartz NMR tube containing the solution of KOH in iPrOH was irradiated with light at a given wavelength for 1 hour. The resulting reaction mixture was analysed by ^{19}F NMR spectroscopy (Figure S15), whereby the fluoride resonance was only detected using light sources at 310 nm, 280 nm and 250-550 nm. Determination of the reaction yield as described in **GP1** revealed complete conversion with light at 280 nm or at 240-550 nm (Table S7).

Table S7: Estimated yields based on fluoride formation (determined by ^{19}F qNMR spectroscopy) assuming KOH conversion according to the equation: 8 KOH + SF_6 + iPrOH → 6 KF + K_2SO_3 + acetone + 5 H_2O.

Wavelength (nm)	Yield (%)
585	0
405	0
365	0
310	0.05
280	>99
250-550	99

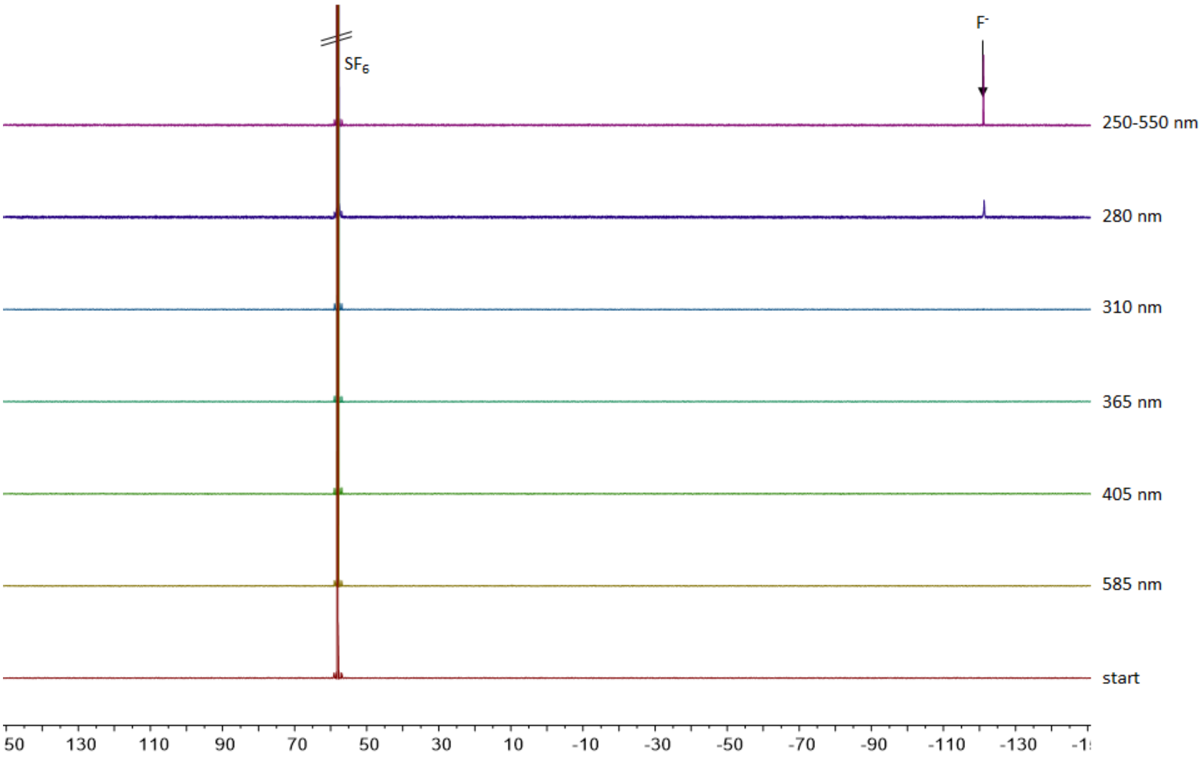

Figure S15: Stacked ^{19}F NMR spectra of reaction mixtures after irradiation with different light sources (LED lamps: 585 nm, 405 nm, 365 nm, 310 nm, 280 nm; mercury-xenon lamp: 250-550 nm).

5.4 Solvent Screening using KOH as Base

Solvent screening was carried out using KOH as the base following **GP1** (KOH: 30.0 mg, 0.46 mmol; solvent: 1 ml, SF_6 pressure: 3 bar, irradiation time: 1 h; see Chapter 4.1) and **GP2** (KOH: 1.0 g, 16.8 mmol; solvent: 15 ml, SF_6 pressure: 2 bar, irradiation time: 2 h; see Chapter 4.2). The results are shown in Table S8 and Figure S16.

Table S8: Estimated yields based on fluoride formation (determined by ^{19}F qNMR spectroscopy) assuming KOH conversion according to the equation: 8 KOH + SF_6 + alcohol → 6 KF + K_2SO_3 + oxidized alcohol + 5 H_2O.

Solvent	Irradiation Setup 1 (**GP1**) Yield (%)	Irradiation Setup 2 (**GP2**) Yield (%)
water	0.1	2
methanol	2	6
ethanol (abs.)	4	1
ethanol (tech.)	>99[c]	29[c]
n-propanol	15	8
isopropanol (tech.)	>99	>99
isopropanol (HPLC grade)	>99	>99
n-butanol	12	4
sec-butanol	>99	>99
2-methyl-2-pentanol	7[a]	6[b]
n-hexanol	2	-
ethylene glycol	0.4	-
1-methoxypropan-2-ol	75	-
2-propoxyethanol	2	-

[a)]Addition of water (0.05 ml) was necessary to dissolve KOH
[b)]0.34 g of KOH was dissolved in 5 ml of 2-methyl-2-pentanol and 0.2 ml H_2O
[c)]technical grade ethanol contains about 1%V/V of isopropanol and butanone. The presence of the latter is the reason for the increased yield compared to absolute ethanol (see Chapter 8.1)

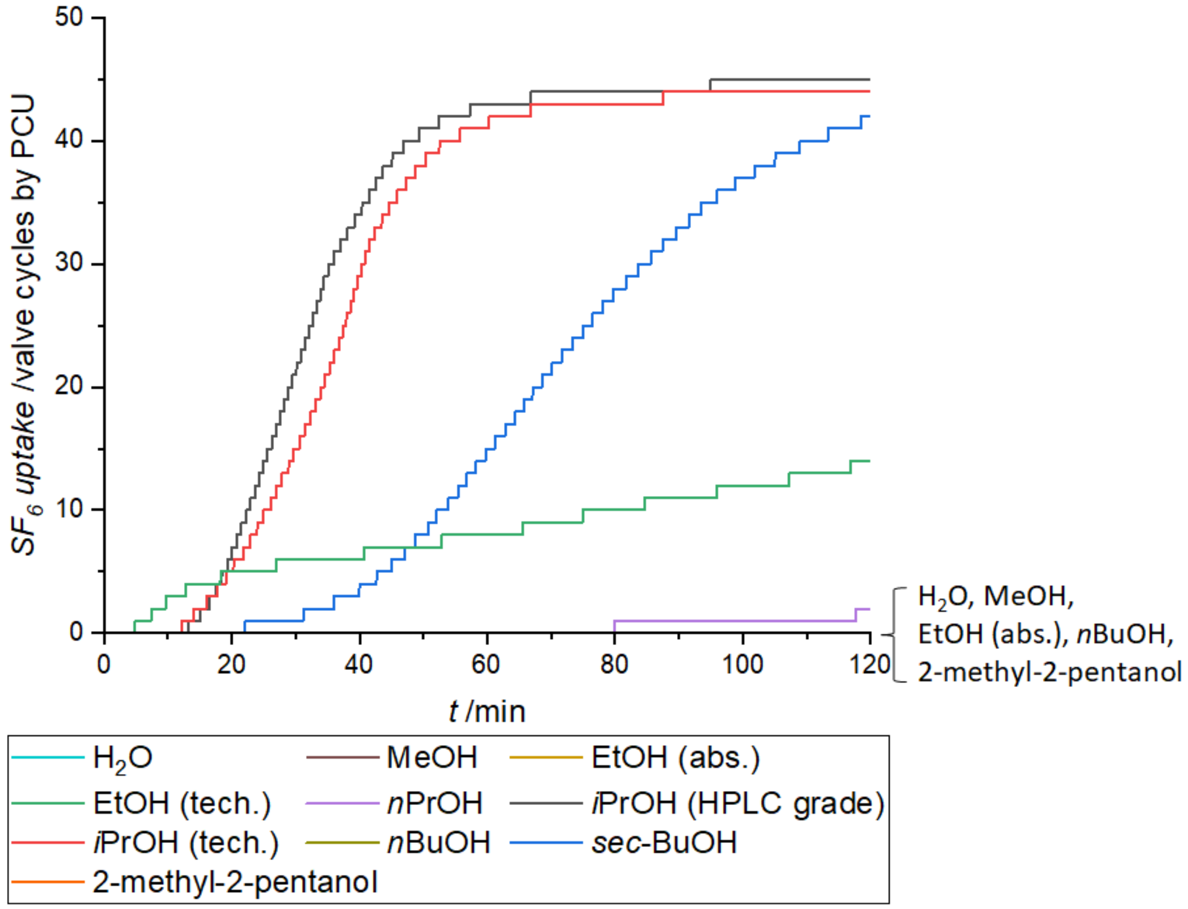

Figure S16: SF_6 uptake time plot obtained by monitoring the KOH/solvent systems (**GP2**) by the PCU. Irradiation of the saturated solution starts at *t* = 0 min.

6 Photochemical Degradation of SF_6 in the KOH/iPrOH System

6.1 Analysis of the Reaction Products

Following **GP2** (see Chapter 4.2), a solution of potassium hydroxide (1.0 g, 16.8 mmol) in HPLC grade isopropanol (15 ml) was degassed and then saturated with 2 bar SF_6. Irradiation of the solution with light at 280 nm led to the formation of a white precipitate (Figure S17). The reaction progress was monitoring using the PCU (Figure S18). Irradiation was stopped after 2 hours and the volatiles were separated by distillation *in vacuo* at ambient temperature for 4 hours. The collected volatile components (11.9 g) and the remaining solid components (1.02 g) were each analysed using several characterization methods (vide infra).

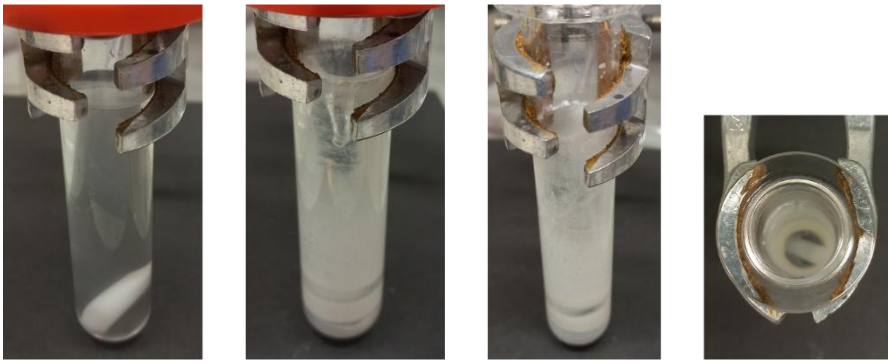

Figure S17: Initial reaction mixture (left), after irradiation for 2 h (second left), after removal of all volatile components (second right) and top-down view of the solid components (right).

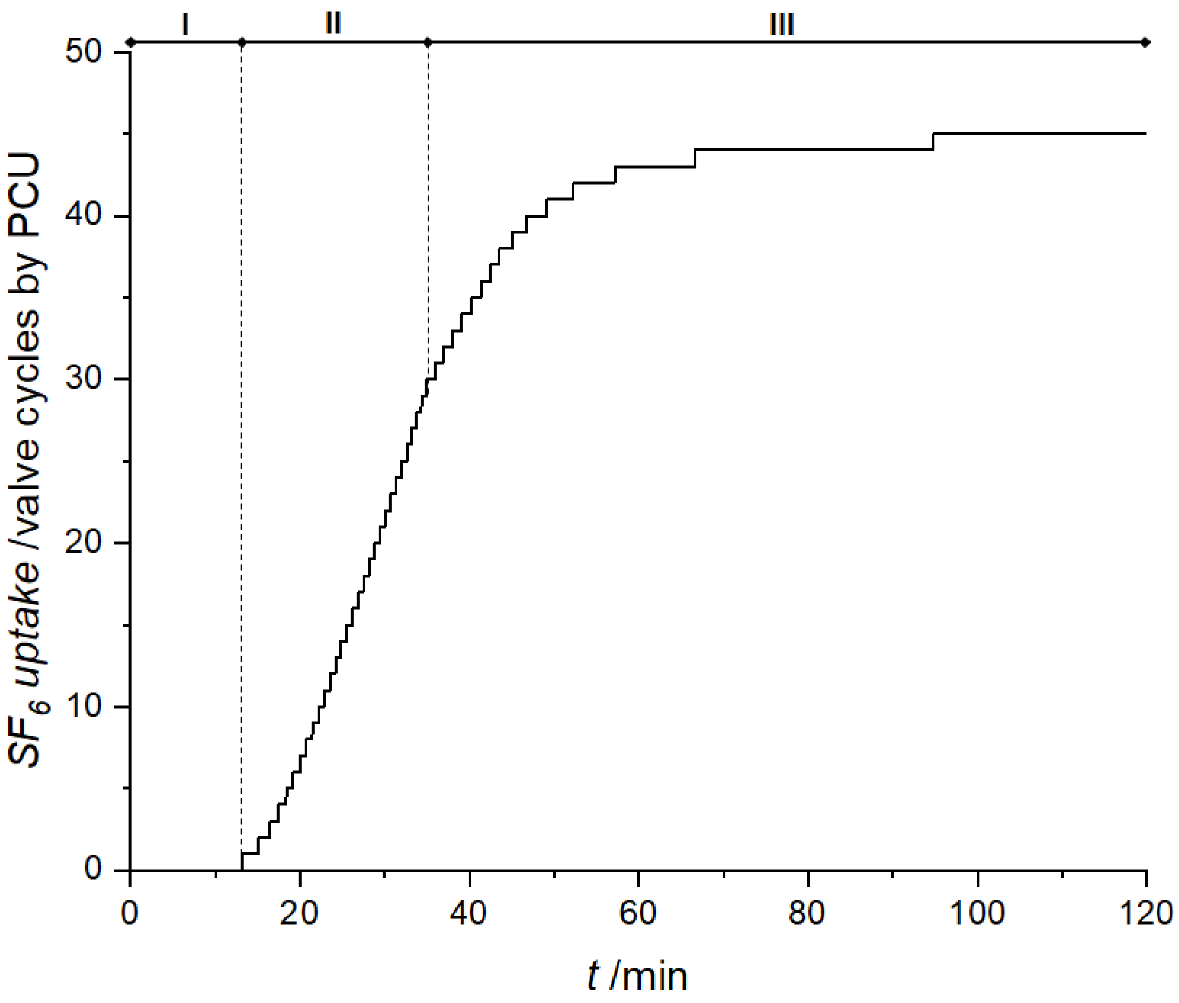

Figure S18: SF_6 uptake time plot of the KOH/iPrOH system (**GP2**) obtained by the PCU. Phase I (acetone build-up), Phase II (diffusion-controlled photocatalysis), Phase III (saturation of the solution phase).

6.1.1 Characterization of the Volatile Components

Analysis by NMR spectroscopy: An aliquot of 0.1 mL of the volatiles was dissolved in $CDCl_3$ (1 mL) and the solution was analysed by NMR spectroscopy: No F-containing compound was detected by ^{19}F NMR spectroscopy apart from traces of SF_6 (Figure S19); ^{1}H and ^{13}C NMR spectroscopy showed the resonances of isopropanol, acetone, water and diisopropyl sulfite (Figure S20; Figure S21). The total amount of these compounds was determined by integration of ^{1}H NMR resonances using pyrazine (13.9 mg, 0.17 mmol) as internal standard: acetone (2.36 mmol); diisopropyl sulfite (0.66 mmol).

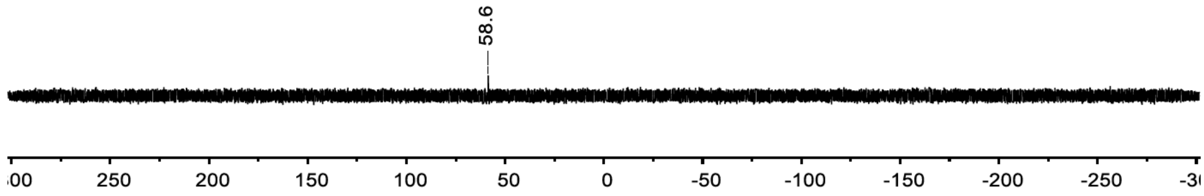

Figure S19: $^{19}F\{^{1}H\}$ NMR spectrum (377 MHz) of the collected volatiles in $CDCl_3$. The resonance at 58.6 ppm is due to traces of SF_6.

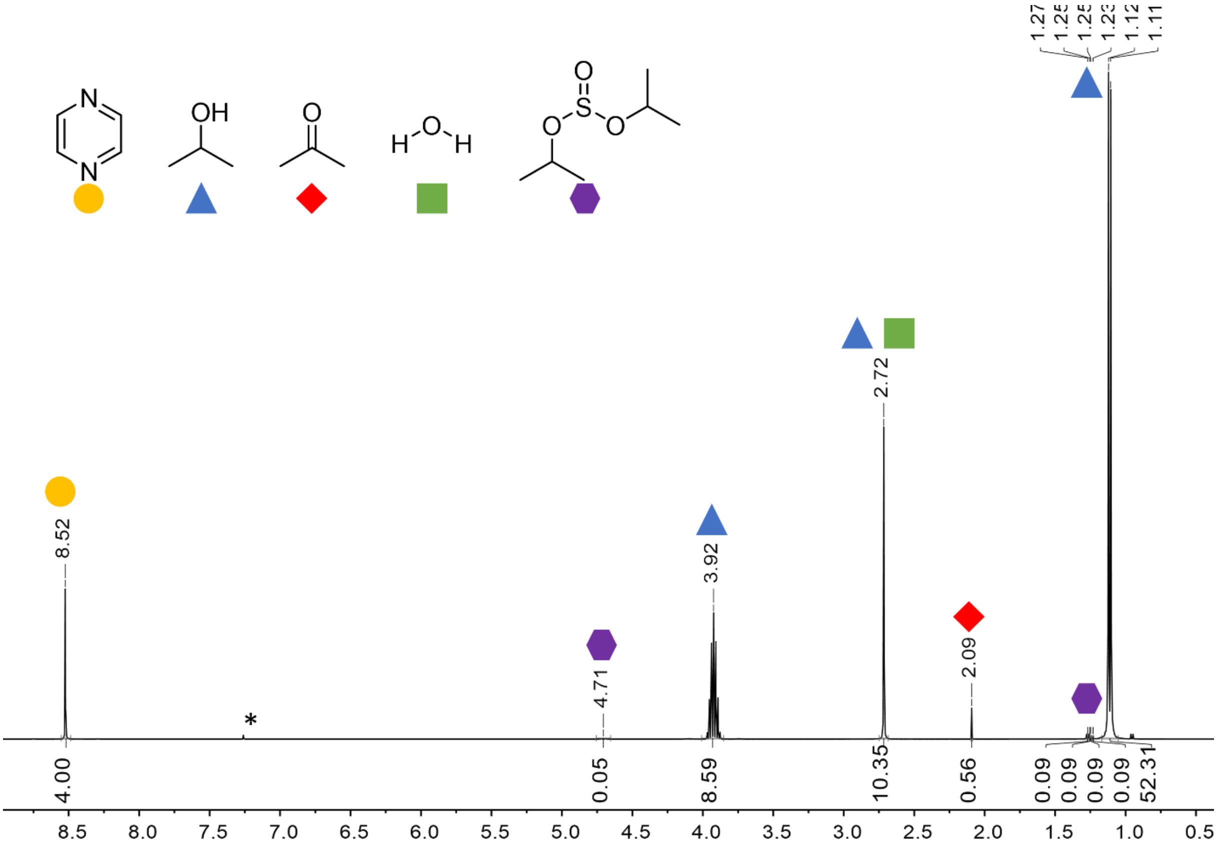

Figure S20: ^{1}H NMR spectrum (400 MHz) of the collected volatiles in $CDCl_3$ (Pyrazine was added as internal standard, *$CHCl_3$). δ = 8.52 (s, $C_4H_4N_2$), 4.71 (sept., $^{3}J_{HH}$ = 6.2 Hz, OS{OC*H*Me$_2$}$_2$), 3.92 (sept., $^{3}J_{HH}$ = 6.1 Hz, HOC*H*Me$_2$), 2.72 (s, *H*OiPr, H_2O), 2.09 (s, OC{CH_3}$_2$), 1.26 (d, $^{3}J_{HH}$ = 6.2 Hz, OS{OCH[CH_3]$_2$}$_2$), 1.23 (d, $^{3}J_{HH}$ = 6.2 Hz, OS{OCH[CH_3]$_2$}$_2$), 1.11 (d, $^{3}J_{HH}$ = 6.1 Hz, HOCH{CH_3}$_2$) ppm.

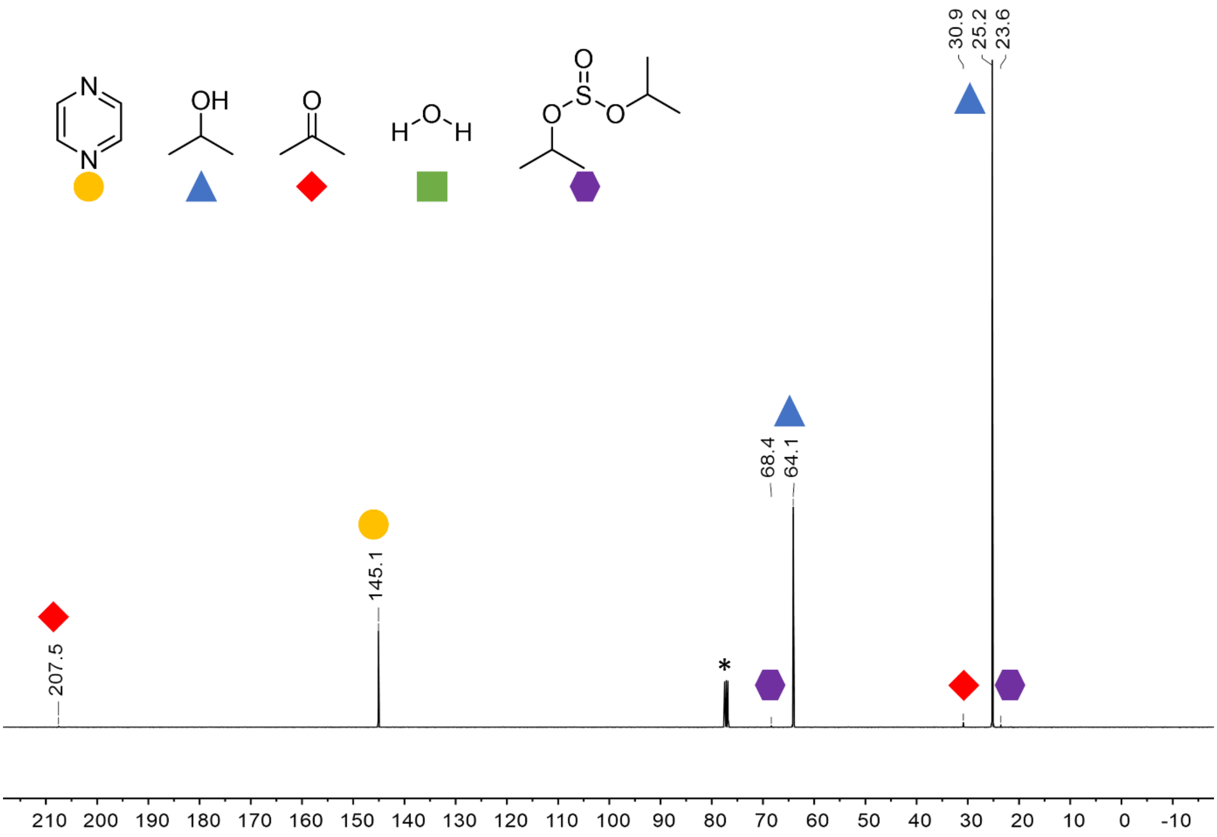

Figure S21: $^{13}C\{^1H\}$ NMR spectrum (101 MHz) of the collected volatiles in $CDCl_3$ (Pyrazine was added as internal standard, *$CDCl_3$). δ = 207.5 (s, O*C*Me_2), 68.4 (s, OS{O*C*HMe_2}$_2$), 64.1 (s, HO*C*HMe_2), 30.9 (s, OC{*C*H_3}$_2$), 25.2 (s, HOCH{*C*H_3}$_2$), 23.6 (s, OS{OCH[*C*H_3]$_2$}$_2$) ppm.

Analysis by GC-MS: One drop each of the volatiles was dissolved in ethyl acetate (1.5 ml) and decalin (1.5 ml) and the solutions were analysed following the established analytical procedure (see Chapter 3.2). The formation of diisopropyl sulfite was confirmed by GC-MS analysis. Additionally, hexylene glycol was detected (Note that the amount is below the detection limit of ^1H NMR spectroscopy). The formation of hexylene glycol can be explained by the reduction of diacetone alcohol which is formed by base catalysed aldol condensation of acetone.

6.1.2 Characterization of the Solid Components

The mixture of inorganic salts was analysed by ^{19}F qNMR, ^{33}S NMR and IR spectroscopy, as well as powder diffraction study (XRD) and capillary electrophoresis (CE).

Analysis by NMR spectroscopy: The mixture of inorganic salts (98.0 mg) was dissolved in water (1 ml) and analysed by NMR spectroscopy. No resonances of C-containing compounds were detected by $^{13}C\{^1H\}$ NMR spectroscopy (Figure S22). No resonances of S-containing compounds were detected by ^{33}S NMR spectroscopy, indicating that no sulfate is formed during the reaction, as discussed in Chapter 3.1 (Figure S23). The amount of fluoride formed in the reaction was determined using ^{19}F qNMR spectroscopy and potassium triflate (10.8 mg, 0.06 mmol) as internal standard (Figure S24): fluoride (13.68 mmol).

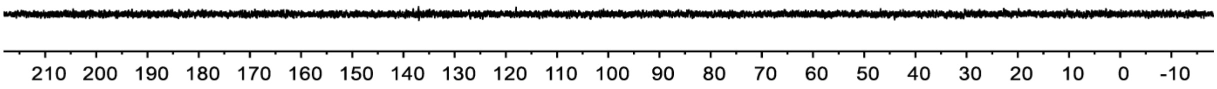

Figure S22: $^{13}C\{^1H\}$ NMR (101 MHz) spectrum of the aqueous solution of inorganic salts.

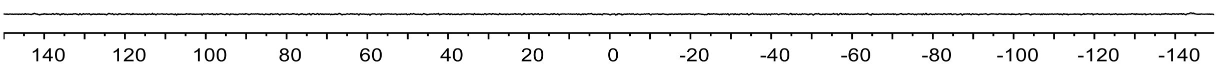

Figure S23: ^{33}S NMR (23 MHz) spectrum of the aqueous solution of inorganic salts.

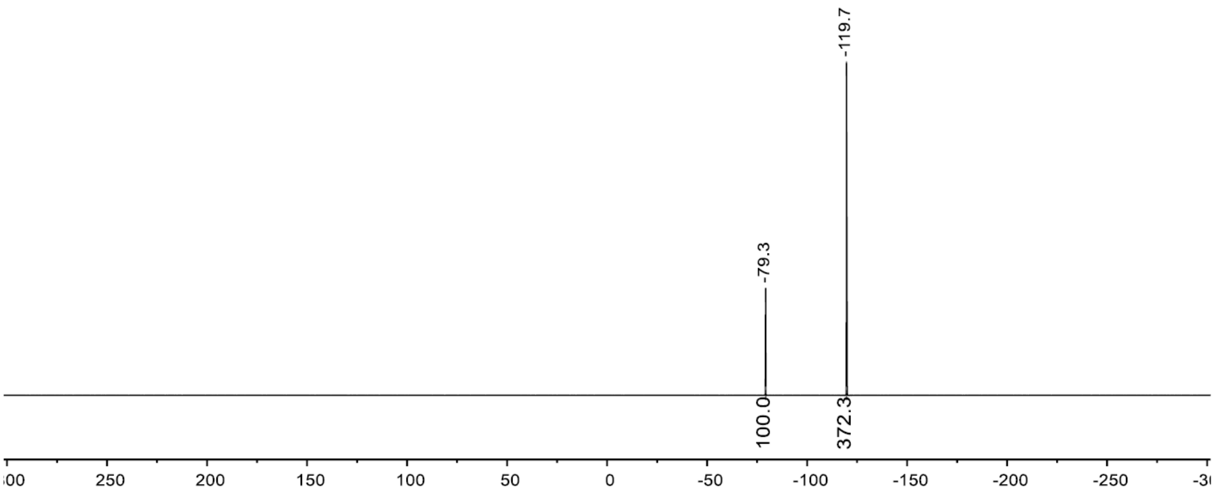

Figure S24: ^{19}F qNMR spectrum (377 MHz) of the aqueous solution of inorganic salts. δ = −78.6 (OTf^-), −119.1 (F^-) ppm.

Analysis by IR spectroscopy: The mixture of inorganic salt was analysed by ATR-IR spectroscopy as described in Chapter 3.3. The resulting IR spectrum shows the characteristic signals of potassium sulfite at 956, 627 and 478 cm^{-1} (Figure S25). Sulfate or thiosulfate were not detected.

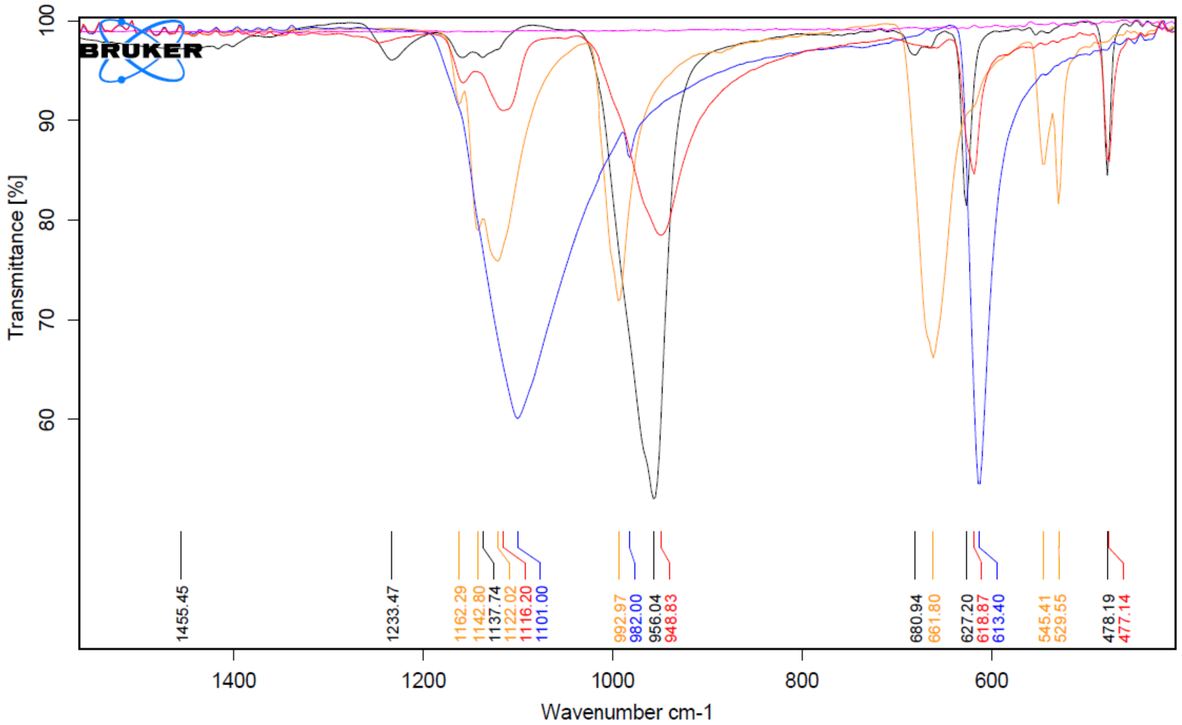

Figure S25: Superimposed IR spectra of the mixture of inorganic salts (black), potassium fluoride (pink), potassium sulfate (blue), potassium sulfite (red), and potassium thiosulfate (orange).

Analysis by powder diffraction (XRD): The mixture of inorganic salts was filled into a capillary under inert gas conditions and subjected to an XRD study (see Chapter 3.4). The resulting powder diffractogram shows the reflexes of potassium fluoride and potassium sulfite (Figure S26). Using

Rietveld refinement, the amount of potassium fluoride (13.17 mmol) and potassium sulfite (1.61 mmol) were determined according to equation 4. Potassium sulfate or potassium thiosulfate were not detected by the XRD study.

$$n(salt) = \frac{m(overall) \cdot m\%(salt)}{M(salt)} \quad (4.)$$

$$n(KF) = \frac{1020\ \text{mg} \cdot 0.75}{58.10 \frac{\text{mg}}{\text{mmol}}} = 13.17\ \text{mmol}$$

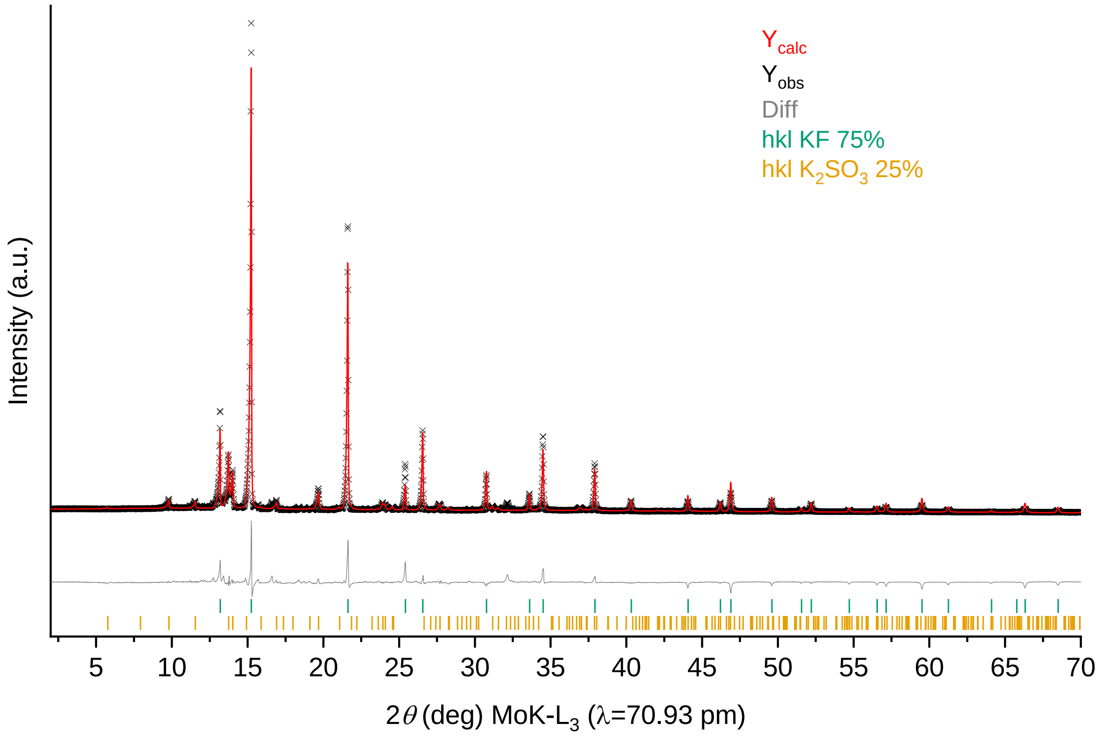

Figure S26: Rietveld refinement of the mixture of inorganic salts.

Analysis by capillary electrophoresis (CE): The mixture of inorganic salts (10.0 mg) was dissolved in water (0.2 L) to afford an aqueous solution with a mass concentration of 50 mg/L, which was analysed by CE as described in Chapter 3.5. Fluoride, sulfate, sulfite and thiosulfate ions were detected by CE (Table S9). As validated by independent experiments (see Chapter 3.5), sulfate is generated from sulfite during CE. Therefore, CE analysis can only be used to detect sulfite, fluoride and thiosulfate anions. Due to the large standard deviation, this method is only used to qualitatively confirm the formation of the anions.

Table S9: Results from the analysis of the mixture of inorganic salts by capillary electrophoresis.

Anion	c_{exp} (mg/L)			Average (mg/L)	Standard deviation (mg/L)	Total amount formed in the reaction (mmol)
	1	2	3			
F^-	11.1	11.6	17.2	13.3	3.4	14.3 ±3.6
SO_3^{2-}	3.4	3.6	3.5	3.5	0.1	0.89 ±0.03
SO_4^{2-}	2.2	2.0	4.0	2.7	0.9	0.6 ±0.2
$S_2O_3^{2-}$	0.3	0.3	0.4	0.3	0.1	0.05 ±0.02

$$n(\text{Ion}) = \frac{\beta(\text{Ion}) \cdot V(\text{dilution})}{M(\text{Ion})} \cdot \frac{m(\text{overall})}{m(\text{CE})} \qquad (5.)$$

$$n(\text{F}^-) = \frac{13.3\ \frac{\text{mg}}{\text{L}} \cdot 0.2\ \text{L}}{19.0\ \frac{\text{mg}}{\text{mmol}}} \cdot \frac{1020\ \text{mg}}{10.0\ \text{mg}} = 14.3\ \text{mmol}$$

<u>Analysis by pH determination:</u> The mixture of inorganic salts (100.0 mg) was dissolved in water (40 ml) and the solution was analysed using a pH meter showing a value of pH = 8.0. The results of the powder diffraction analysis show that 100 mg of the mixture of inorganic salts consists of 75 mg potassium fluoride and 25 mg potassium sulfite. Using the pK_B values of potassium fluoride (10.86) and potassium sulfite (6.80) as well as the equation 6 for weak bases the pH value of the aqueous solutions of potassium fluoride (75.0 mg, 1.29 mmol, 32.25 mmol/L) and potassium sulfite (25.0 mg, 0.16 mmol, 4.00 mmol/L) can be calculated. The measured pH value of the mixture of inorganic salts (8.0) is in between the calculated pH values of potassium fluoride (7.82) and potassium sulfite (9.40). These results show that potassium hydroxide was completely consumed.

$$pH = 14 - \frac{1}{2} \cdot \left(pK_B - \log\left(c_0(B)\right)\right) \qquad (6.)$$

$$pH(\text{KF}) = 14.00 - \frac{1}{2} \cdot \left(10.86 - \log\left(0.03225 \frac{\text{mol}}{\text{L}}\right)\right) = 7.82$$

$$pH(\text{K}_2\text{SO}_3) = 14.00 - \frac{1}{2} \cdot \left(6.80 - \log\left(0.004 \frac{\text{mol}}{\text{L}}\right)\right) = 9.40$$

6.1.3 Determination of the Reaction Yield

The results from the analysis of the solid and volatile products (Chapter 6.1.1 and 6.1.2) are summarized in Table S10. The analyses show that the following compounds are formed in the photochemical reaction: KF, K_2SO_3, diisopropyl sulfite, acetone. Note that CE indicated the formation of small amounts of $K_2S_2O_3$, but it was not detected by XRD. Similarly, traces of hexylene glycol were detected by GC-MS, but the amount is below the detection limit of ^{1}H NMR spectroscopy. Therefore, both compounds are not considered in the overall reaction equation.

Table S10: Overview of the analysis of the volatile and solid components of the product mixture through the different analytical methods shown in chapter 6.1.1 and 6.1.2. (- = not detectable by the method, ✓ = detected, but not quantified)

Molecule	^{1}H NMR (mmol)	GC-MS	^{19}F qNMR (mmol)	^{33}S NMR	IR	CE (mmol)	XRD (mmol)
F^-	-	-	13.68	-	-	✓	13.17
SO_3^{2-}	-	-	-	-	✓	✓	1.61
Diisopropyl sulfite	0.66	✓	-	-	-	-	-
Acetone	2.36	-	-	-	-	-	-

Based on the analytical data, equations 7 and 8 need to be considered to describe the photochemical SF_6 degradation reaction:

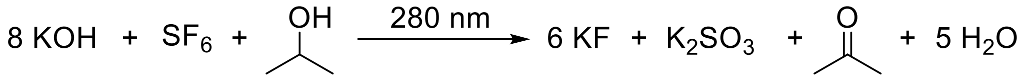

(7.)

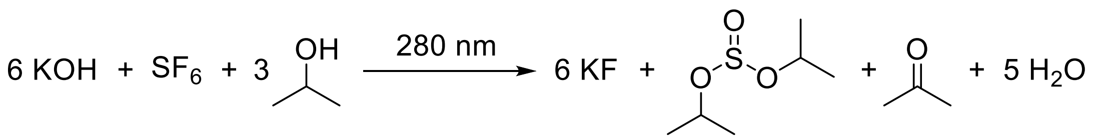

(8.)

The contribution of the individual reactions to the overall reaction equation was estimated from the quantities of the sulfur-containing products sulphite and diisopropyl sulfite (equation 9). Note that the total amount of sulfur atoms formed (2.27 mmol) should give a total amount of fluoride of 2.27 mmol x 6 = 13.68 mmol, which is consistent with the experimentally determined value (Table S10).

$$n(\mathrm{S}_{total}) = n(\mathrm{SO_3}^{2-}) + n((\mathrm{iPrO})_2\mathrm{SO}) \qquad (9.)$$

$$n(\mathrm{S}_{total}) = 1.61\ \mathrm{mmol} + 0.66\ \mathrm{mmol} = 2.27\ \mathrm{mmol}$$

Afterwards the ratio of sulfite and diisopropyl sulfite of the total sulfur amount was calculated (equation 10). This ratio equates to the weight of the reaction equation on the overall reaction equation.

$$w_i = \frac{n(\mathrm{S}_{compound})}{n(\mathrm{S}_{total})} \qquad (10.)$$

$$w_7 = \frac{1.61\ \mathrm{mmol}}{2.27\ \mathrm{mmol}} = 0.71$$

Table S11: Weighting of the reaction equations 7 – 8.

Equation *i*	weighting factor w_i
7	0.71
8	0.29

Calculation of the weighting factors according to equation 10 (Table S11) allowed to merge the individual reaction equations 7 and 8 into the overall reaction equation 11:

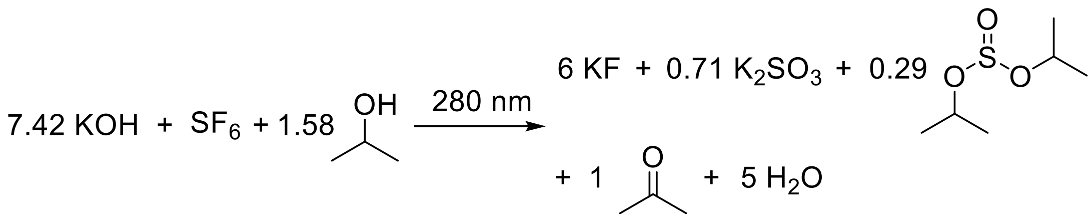

(11.)

According to reaction equation 11, the initial amount of KOH (16.79 mmol) should produce the following products: 13.58 mmol KF, 1.61 mmol K_2SO_3, 0.66 mmol $(iPrO)_2SO$, 2.26 mmol acetone and 11.30 mmol H_2O, which is consistent with the experimental values (Table S10) and a quantitative yield.

It is important to note that this weighting only applies to the SF_6 degradation using the KOH/iPrOH system and irradiation setup 2, because the degree of diisopropyl sulfite to potassium sulfite conversion should strongly depend on the reaction conditions (scale, additives, irradiation setup,…).

6.2 Parameters Influencing the Reaction Rate

6.2.1 Molecular Oxygen

To investigate the influence of oxygen on the KOH/iPrOH system, the reaction was carried out using different gas atmospheres following **GP2** (KOH: 1.0 g, 16.8 mmol; isopropanol (HPLC grade): 15 ml; SF_6 pressure: 2 bar; irradiation time: 2 h; see chapter 4.2). Full conversion of potassium hydroxide was observed for each reaction. However, the reaction is delayed in the presence of air and the rate of the reaction is slower in the presence of an argon atmospheres (Table S12; Figure S27).

Table S12: Yields based on fluoride formation (determined by ^{19}F qNMR spectroscopy) assuming KOH conversion according to equation 11: 7.42 KOH + SF_6 + 1.58 iPrOH → 6 KF + 0.71 K_2SO_3 + 0.29 $(iPrO)_2SO$ + 1 acetone + 5 H_2O.

Starting atmosphere	Maximum degradation rate (g/h)	Yield (%)
SF_6	0.7	>99
Air	0.5	94
Argon	0.4	98

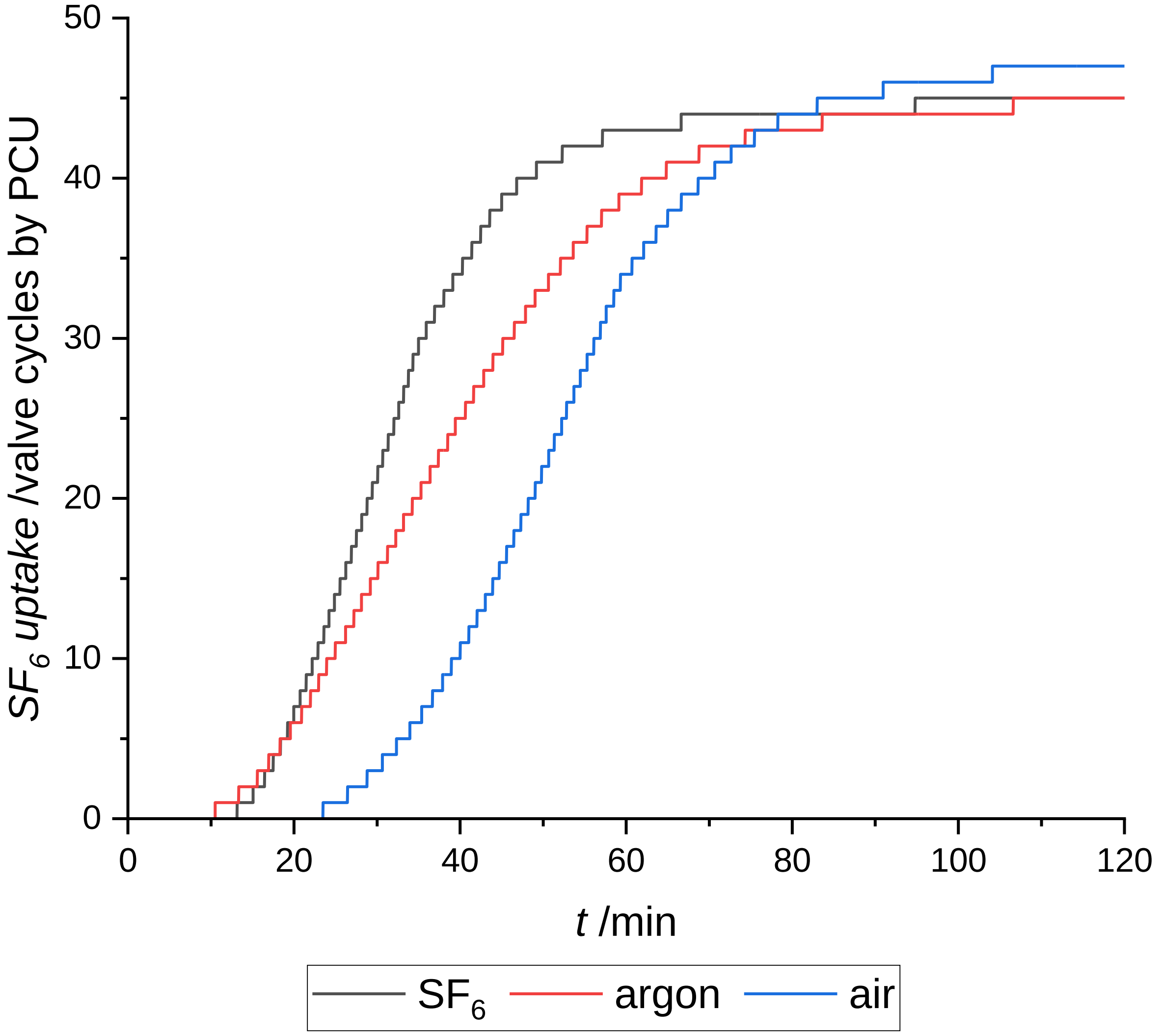

Figure S27: SF_6 uptake time plot obtained by the PCU for the KOH/iPrOH system (**GP2**) with different initial gas atmospheres showing the influence of the initial gaseous components on the SF_6 uptake.

6.2.2 Pressure of the SF_6 Gas

To investigate the influence of the SF_6 concentration on the KOH/iPrOH system, the reaction was carried out using different SF_6 pressures following **GP2** (KOH: 1.0 g, 16.8 mmol; isopropanol (HPLC grade): 15 ml; irradiation time: 2 h; see chapter 4.2). Full conversion of the potassium hydroxide was observed for each reaction. Note that the amount of SF_6 added to the reaction chamber in each cycle of the magnetic valve depends on the pressure difference between the SF_6 gas cylinder and the reaction vessel leading to a lower number of cycles with greater pressure difference (Table S13; Figure S28).

Table S13: Yields based on fluoride formation (determined by ^{19}F qNMR spectroscopy) assuming KOH conversion according to equation 11: 7.42 KOH + SF_6 + 1.58 iPrOH → 6 KF + 0.71 K_2SO_3 + 0.29 $(iPrO)_2SO$ + 1 acetone + 5 H_2O.

SF_6 pressure (bar)	Maximum degradation rate (g/h)	Yield (%)
2.0	0.7	>99
1.5	0.6	>99
1.0	0.4	97

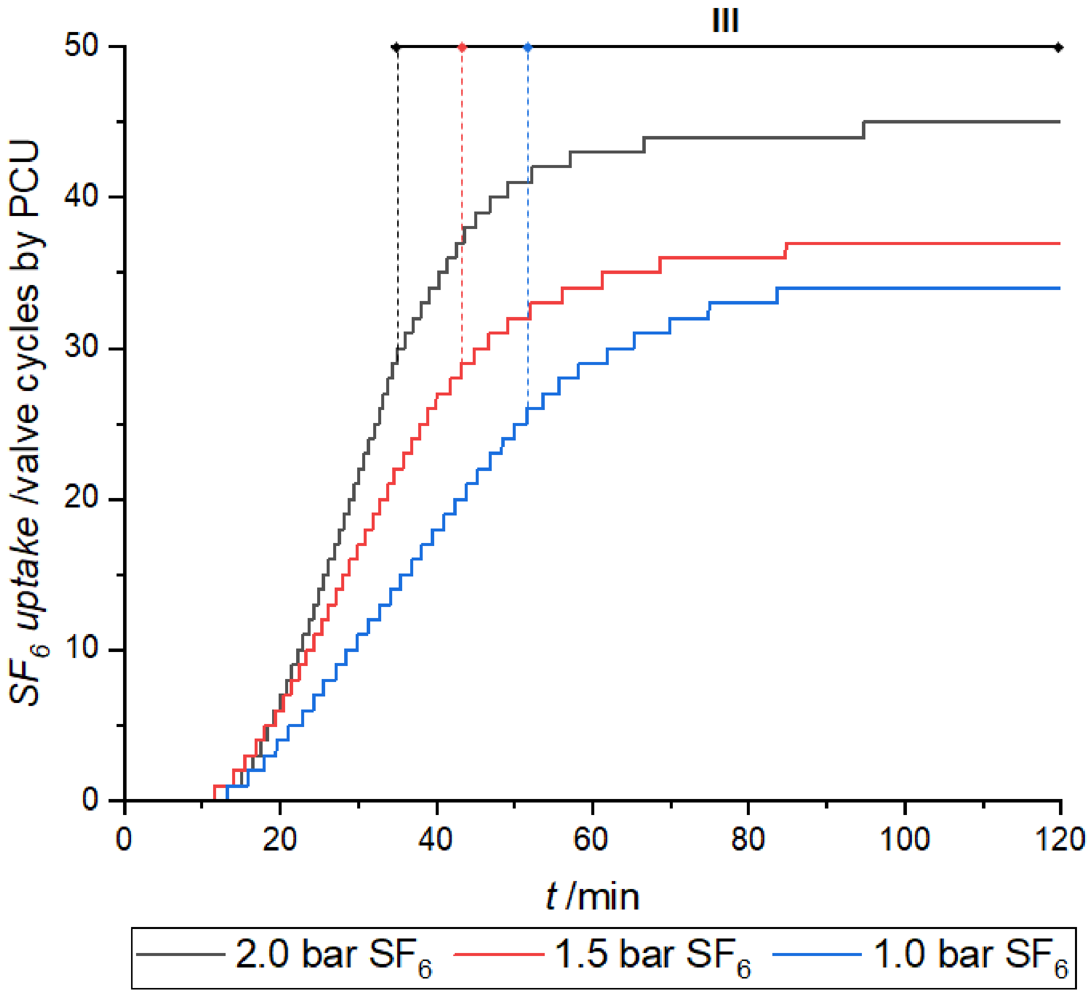

Figure S28: SF_6 uptake time plot obtained by the PCU for the KOH/iPrOH system (**GP2**) with different SF_6 gas pressure. Note that the amount of SF_6 added into the reaction vessel per cycle is higher at lower SF_6 pressure.

6.2.3 Concentration of KOH

To investigate the influence of the base concentration the KOH/iPrOH system, the reaction was carried out with different potassium hydroxide concentrations following **GP2** (isopropanol (HPLC grade): 15 ml; SF_6 pressure: 2 bar; irradiation time: 2 h; see chapter 4.2). Full conversion of potassium hydroxide was observed for each reaction (Table S14; Figure S29). The SF_6 uptake starts earlier with low KOH concentration (0.5 g KOH) and is slower in the beginning with higher KOH concentration (2 g KOH), presumably as a result of the decreasing solubility of SF_6 in more polar solutions. However, the maximum SF_6 uptake rate is comparable in all reactions.

Table S14: Yields based on fluoride formation (determined by ^{19}F qNMR spectroscopy) assuming KOH conversion according to equation 11: 7.42 KOH + SF_6 + 1.58 iPrOH → 6 KF + 0.71 K_2SO_3 + 0.29 $(iPrO)_2SO$ + 1 acetone + 5 H_2O.

Potassium hydroxide (g)	Maximum degradation rate (g/h)	Yield (%)

1.5	0.7	96
1.0	0.7	>99
0.5	0.7	97

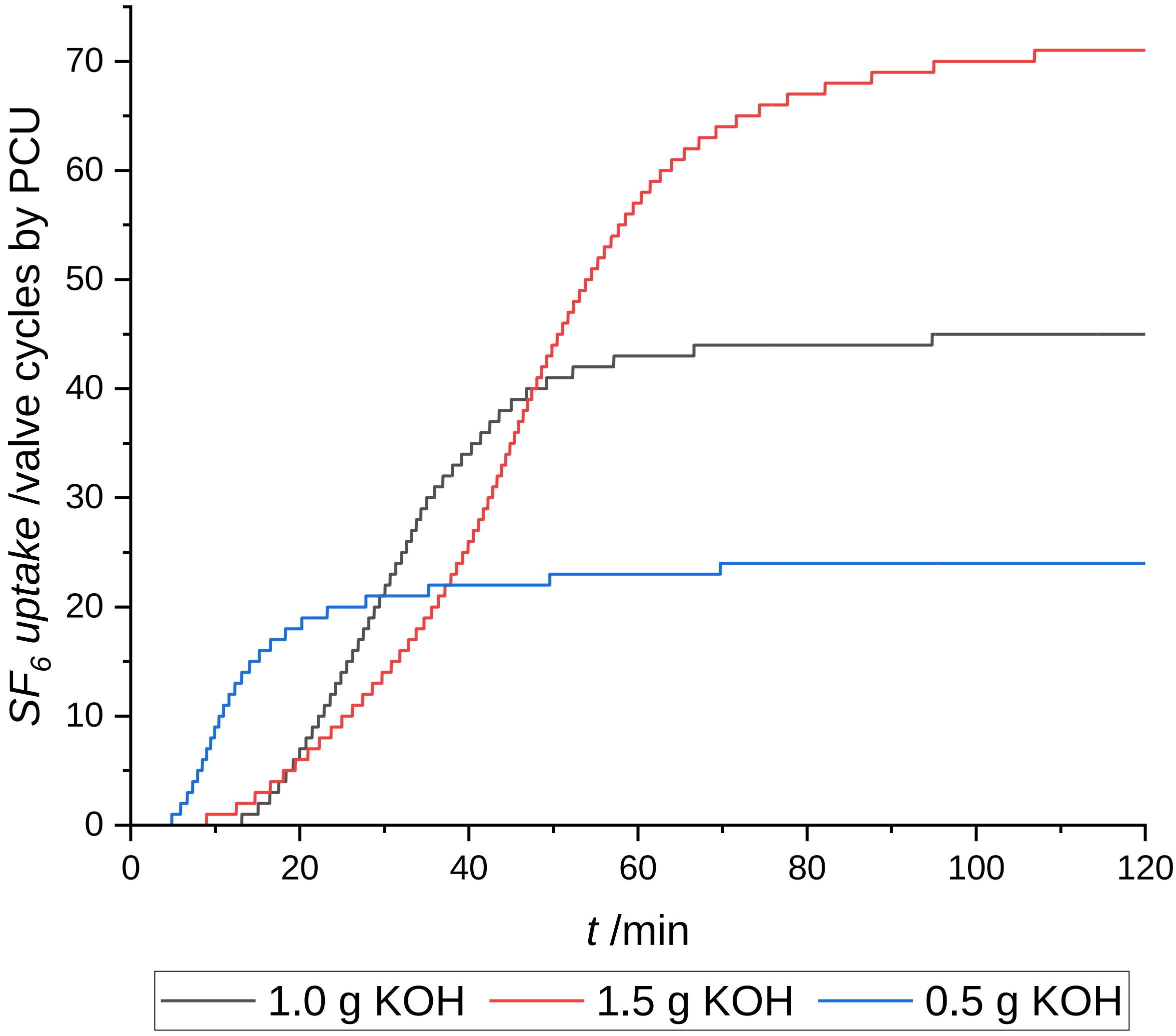

Figure S29: SF_6 uptake time plot for the KOH/iPrOH system (**GP2**) obtained by the PCU showing the influence of the potassium hydroxide concentration on the reaction rate.

6.2.4 Irradiation Power

To investigate the effect of irradiation power, varying numbers of LED lamps (see Figure S30) were used for the KOH/iPrOH system following **GP2** (KOH: 1.0 g, 16.8 mmol; isopropanol (HPLC grade): 15 ml; SF_6 pressure: 2 bar; irradiation time: 2 h; see chapter 4.2). Complete conversion of potassium hydroxide within 2 hours was achieved with 3, 5, 10, and 20 LEDs, whereas irradiation with a single LED resulted in only 67% conversion over the same period (Table S15, Figure S31). The SF_6 degradation rate increased with the number of LEDs, suggesting that the reaction rate is photon-limited. However, no significant rate enhancement was observed when using 20 LEDs compared to 10 LEDs, indicating that the reaction becomes mass transport-limited at higher irradiation powers. It is worth noting that the reaction vessel experienced noticeable warming when using 10 LEDs, and especially 20 LEDs, which influenced the solubility of SF_6 and consequently affected the total number of valve cycles performed by the PCU.

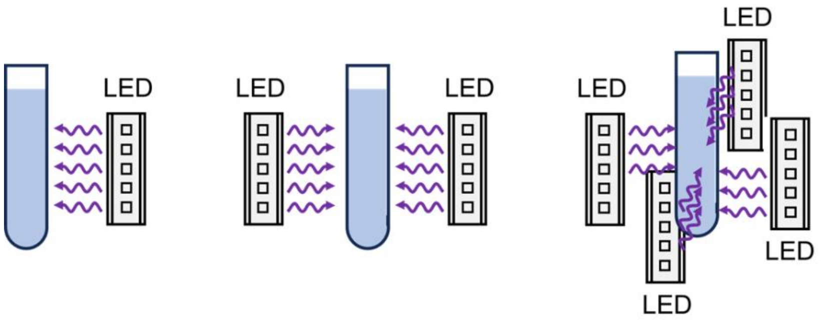

Figure S30: Schematic representation of the irradiation experiments (setup 2) using varying numbers of LEDs. Left: 1, 3, 5 LEDs (a single LED array was powered, with some of its fife LEDs covered); middle: 10 LEDs (two LED arrays positioned at a 180° angle to each other); right: 20 LEDs (four LED arrays positioned at 90° angles to each other).

Table S15: Yields based on fluoride formation (determined by ^{19}F qNMR spectroscopy) assuming KOH conversion according to equation 11: 7.42 KOH + SF_6 + 1.58 iPrOH → 6 KF + 0.71 K_2SO_3 + 0.29 $(iPrO)_2SO$ + 1 acetone + 5 H_2O.

LEDs	Maximum degradation rate (g/h)	Yield (%)
20	1.5	>99
10	1.5	>99
5	0.7	>99
3	0.5	95
1	0.2	67

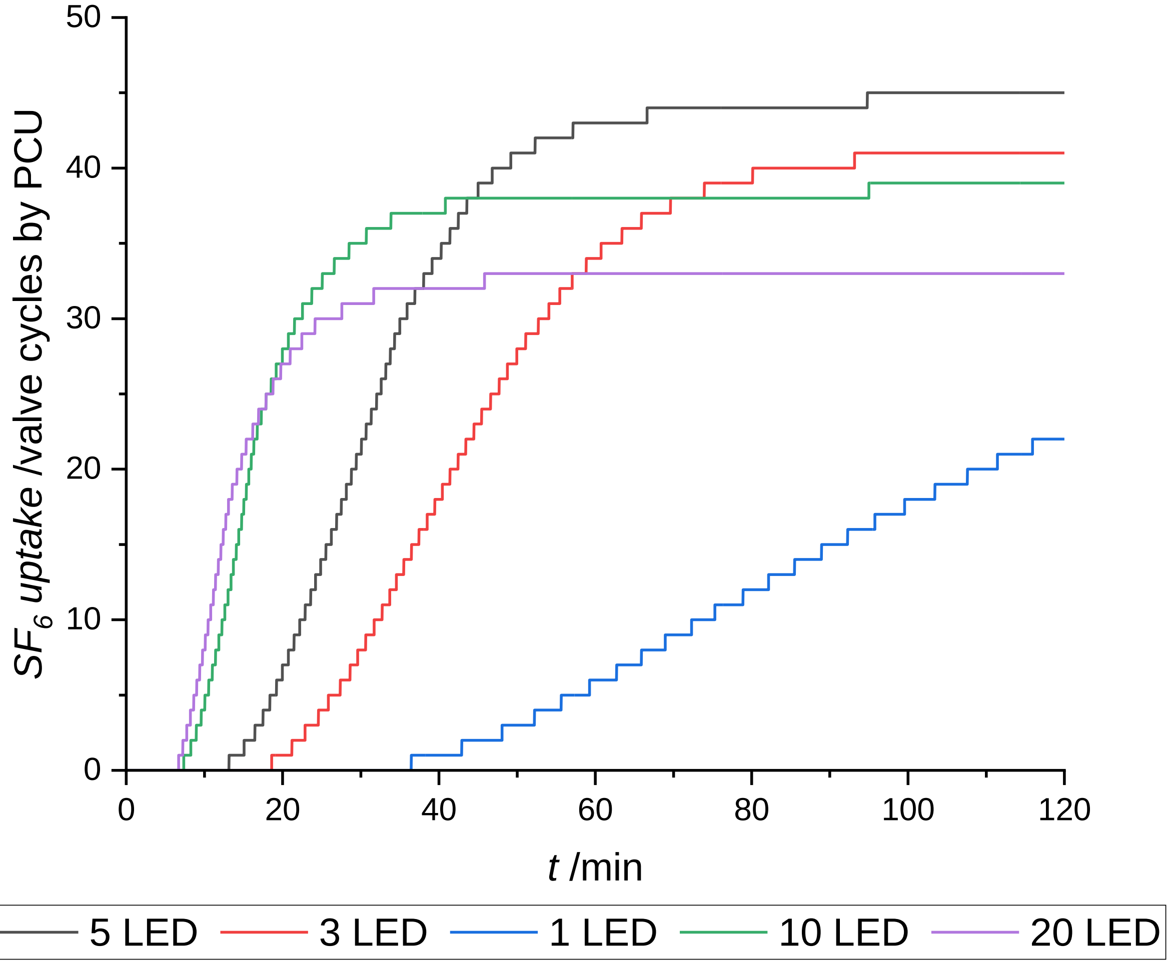

Figure S31: SF_6 uptake time plot for the KOH/iPrOH system (**GP2**) obtained by the PCU showing the influence of the light intensity on the reaction rate.

6.2.5 Irradiation time

To investigate the influence of the irradiation time on the KOH/iPrOH system, three experiments were carried out with varying irradiation times following **GP2** (KOH: 1.0 g, 16.8 mmol; isopropanol (HPLC grade): 15 ml; SF_6 pressure: 2 bar; see chapter 4.2). The experiment with 30 minutes of irradiation shows a similar SF_6 uptake profile like the standard experiment (irradiation for 120 minutes) and similar reaction yield (Table S16; Figure S32). By contrast, incomplete conversion of KOH is observed when the irradiation is stopped after 20 minutes.

Table S16: Yields based on fluoride formation (determined by ^{19}F qNMR spectroscopy) assuming KOH conversion according to equation 11: 7.42 KOH + SF_6 + 1.58 iPrOH → 6 KF + 0.71 K_2SO_3 + 0.29 $(iPrO)_2SO$ + 1 acetone + 5 H_2O.

Irradiation time (min)	Maximum degradation rate (g/h)	Yield (%)
120 min	0.7	>99
30 min	0.5	93
20 min	0.5	56

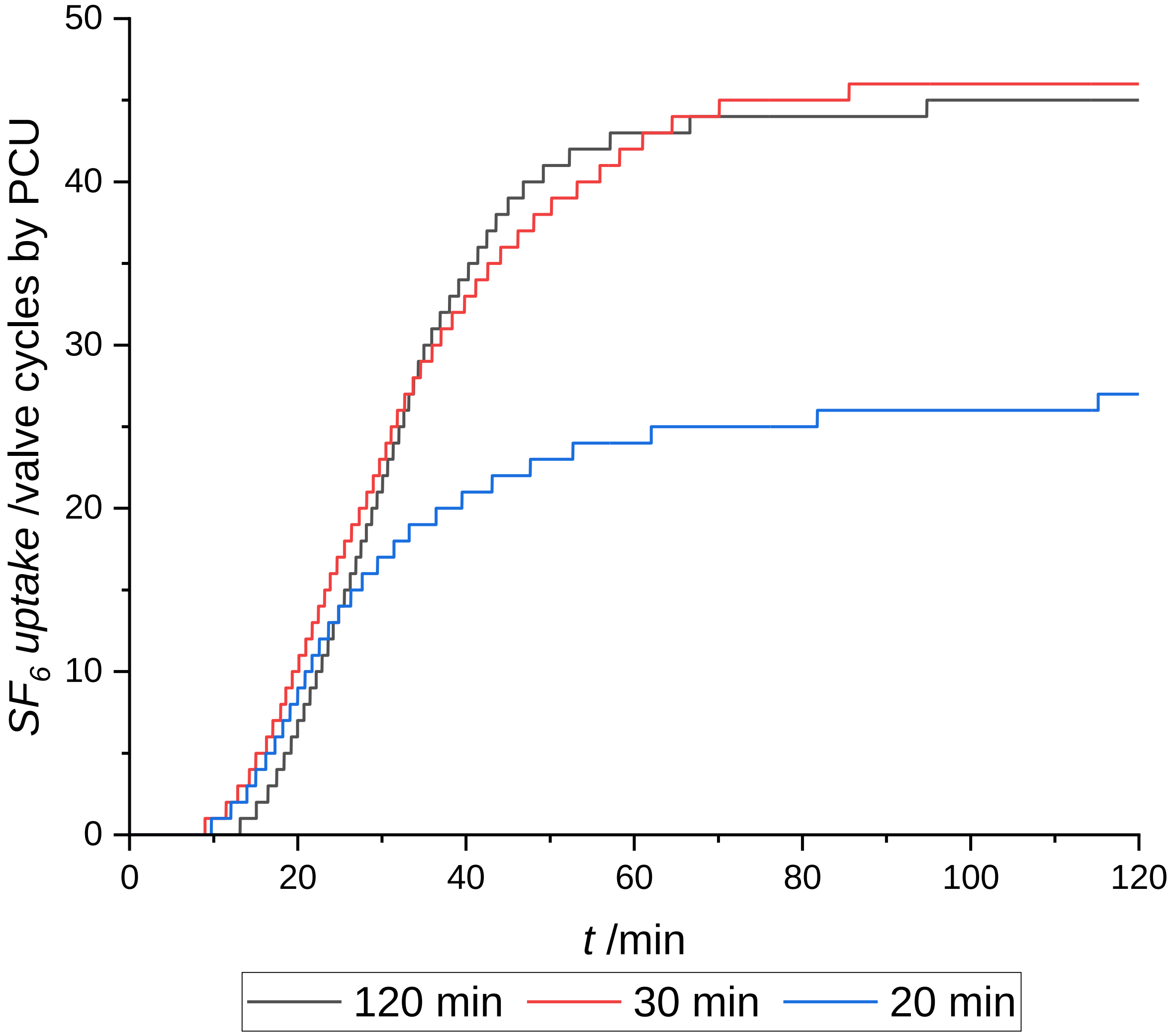

Figure S32: SF_6 uptake time plot for the KOH/iPrOH system (**GP2**) obtained by the PCU showing the influence of the irradiation time on the reaction rate.

6.2.6 Influence of phase mixing on the reaction rate

The decomposition reaction of SF_6 with the KOH/iPrOH system was carried out without stirring to investigate the influence of the diffusion of the reactants on the reaction rate. Prior to the start of the irradiation the reaction mixture was saturated with SF_6 following **GP2** (KOH: 1.0 g, 16.8 mmol;

isopropanol (HPLC grade): 15 ml; SF_6 pressure: 2 bar; irradiation time: 2 h; see chapter 4.2). This experiment shows that the SF_6 uptake is significantly slower without stirring, and it is incomplete after 120 minutes (Table S17; Figure S33).

Table S17: Yields based on fluoride formation (determined by ^{19}F qNMR spectroscopy) assuming KOH conversion according to equation 11: 7.42 KOH + SF_6 + 1.58 iPrOH → 6 KF + 0.71 K_2SO_3 + 0.29 $(iPrO)_2SO$ + 1 acetone + 5 H_2O.

Condition	Maximum degradation rate (g/h)	Yield (%)
With stirring	0.7	>99
Without stirring	0.3	83

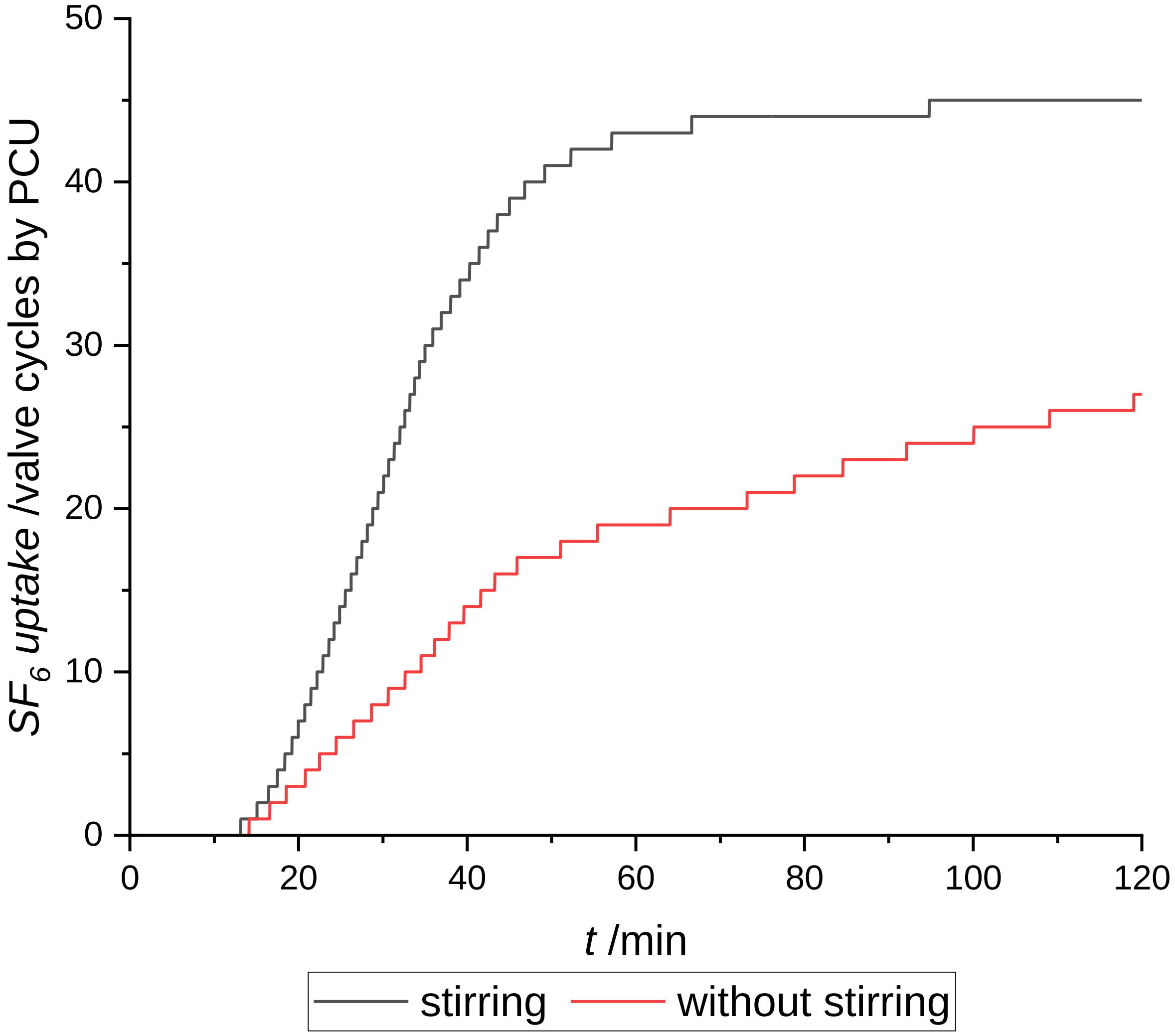

Figure S33: SF_6 uptake time plot for the KOH/iPrOH system (**GP2**) obtained by the PCU showing the influence of the diffusion of SF_6 through stirring on the reaction rate.

6.3 Scaleup of the KOH/iPrOH System

6.3.1 Irradiation Setup 3

For scaleup of the KOH/iPrOH system to a liquid volume of 80 mL, irradiation setup 3 was used with different potassium hydroxide concentrations following **GP3** (isopropanol (tech. grade): 80 ml; SF_6 pressure: 1 bar; see chapter 4.3).

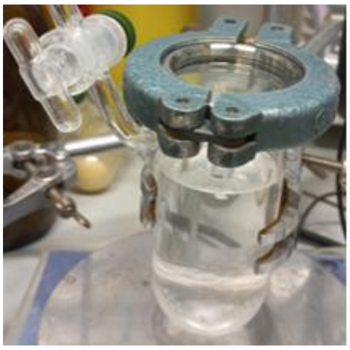

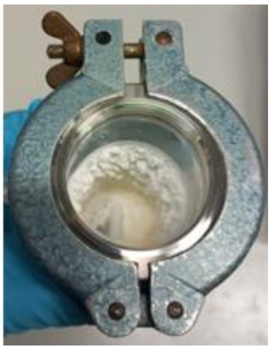

Figure S34: Picture of the reaction vessel (KOH = 10 g) (left) and after irradiation (67 h) and removal of the volatile compounds (right).

Table S18: Estimated yields based on fluoride formation (determined by ^{19}F qNMR spectroscopy) assuming KOH conversion according to the following reaction equation: 8 KOH + SF_6 + iPrOH → 6 KF + K_2SO_3 + acetone + 5 H_2O.

Potassium hydroxide (g)	Irradiation time (h)	Yield (%)
5.0	42	98
10.0	67	90
15.0*	150	85

*Due to the limited solubility of KOH in iPrOH, the initial reaction mixture was a suspension.

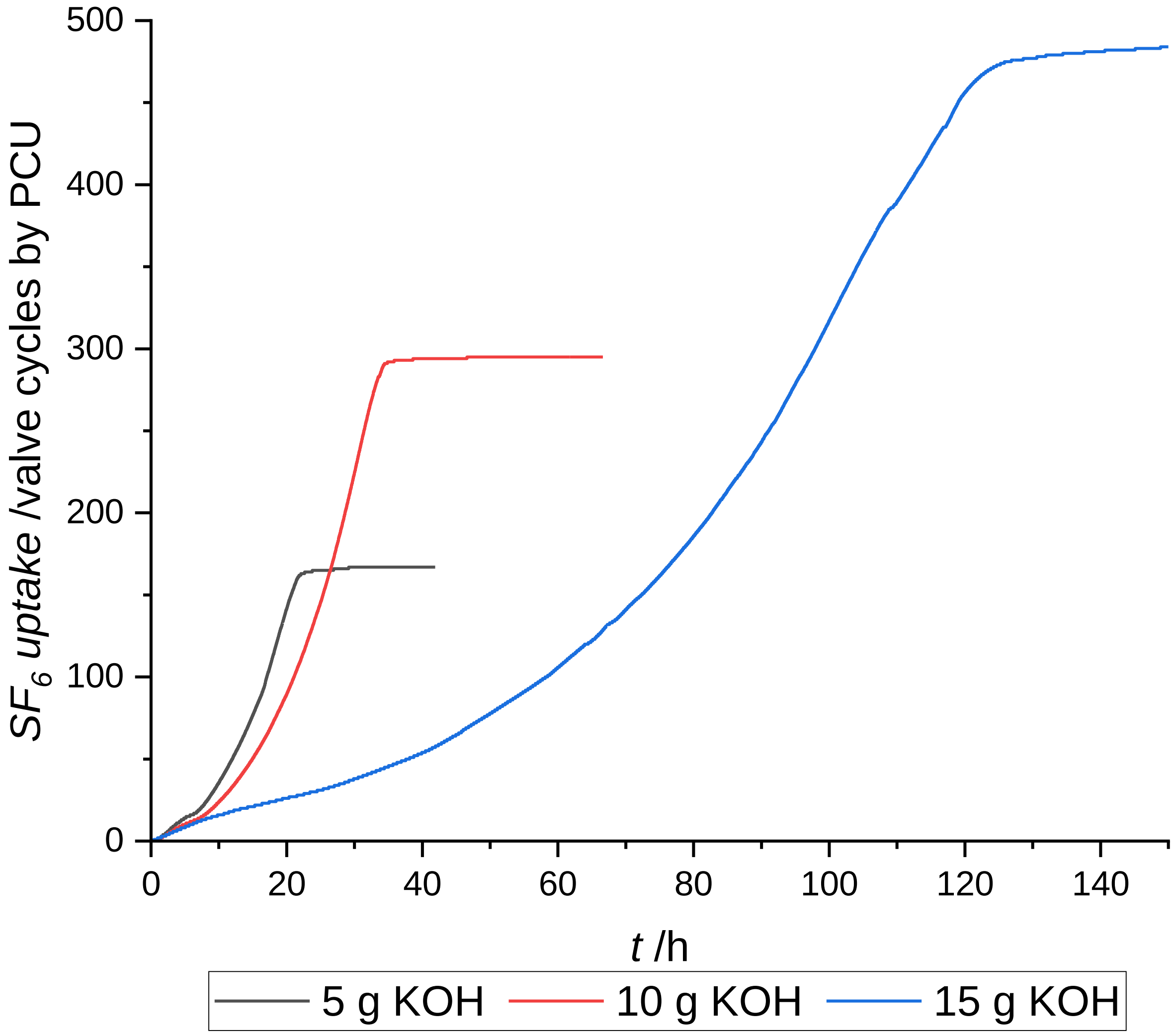

Figure S35: SF_6 uptake time plot for the KOH/iPrOH system (**GP3**) obtained by the PCU showing the influence of the potassium hydroxide concentration on the SF_6 decomposition rate. Note that in the case of the 15 g KOH experiment, the initial reaction mixture was a suspension due to the limited solubility.

6.3.2 Irradiation Setup 4

For scaleup of the KOH/iPrOH system to a liquid volume of 850 mL, irradiation setup 4 was used following **GP4** (KOH: 60 g, 1.0 mol; isopropanol (tech. grade): 850 ml; SF_6 pressure: 2 bar; irradiation time 24 h; see chapter 4.4). The SF_6 consumption was monitored using the PCU (Figure S37). After 24 hours (Figure S36), the liquid phase was separated from the precipitate by decantation and the solution was analysed by GC-MS and ^{1}H NMR as described in Chapter 6.1.1. The solid residue was transferred into a 250 mL round bottom flask using small amounts of water to ensure complete transfer. The volatiles were evaporated under reduced pressure to afford a mixture of inorganic salts (59.38 g), which was analysed by ^{19}F qNMR and capillary electrophorese using the procedure described in Chapter 6.1.2. The results obtained from the analysis of the liquid phase and the precipitate are summarized in Table S19.

Table S19: Overview of the analysis results of the products obtained from the SF_6 decomposition with the KOH/iPrOH system according to **GP4**. (- = not detectible with the method, X = not detected, ✓ = detected)

Component	GC-MS	^{1}H NMR (mmol)	^{19}F qNMR (mmol)	CE (mmol)
F^-	-	-	754	✓
SO_3^{2-}	-	-	-	✓
$S_2O_3^{2-}$	-	-	-	✓
Diisopropyl sulfite	✓	6	-	-
Acetone	-	128	-	-

Following the method described in chapter 6.1.3, the overall reaction equation 12 was used to determine the yield. Yield: 99%

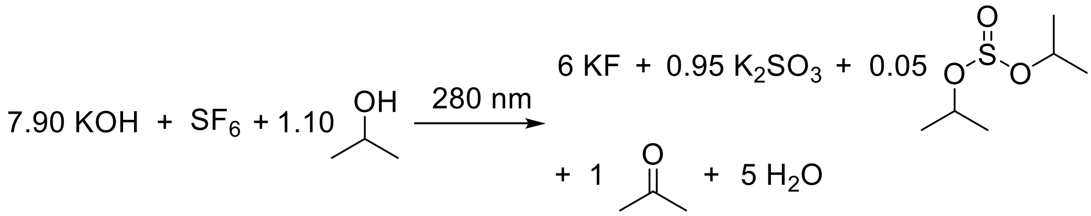

(12.)

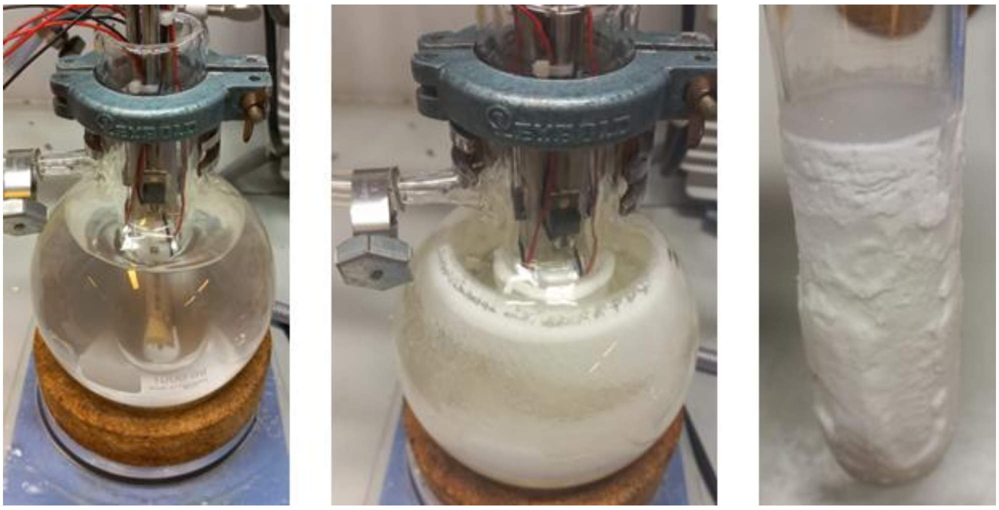

Figure S36: Initial reaction mixture (left), reaction mixture after irradiation for 24 h (central) and the inner quartz tube (right) after irradiation for 24 h.

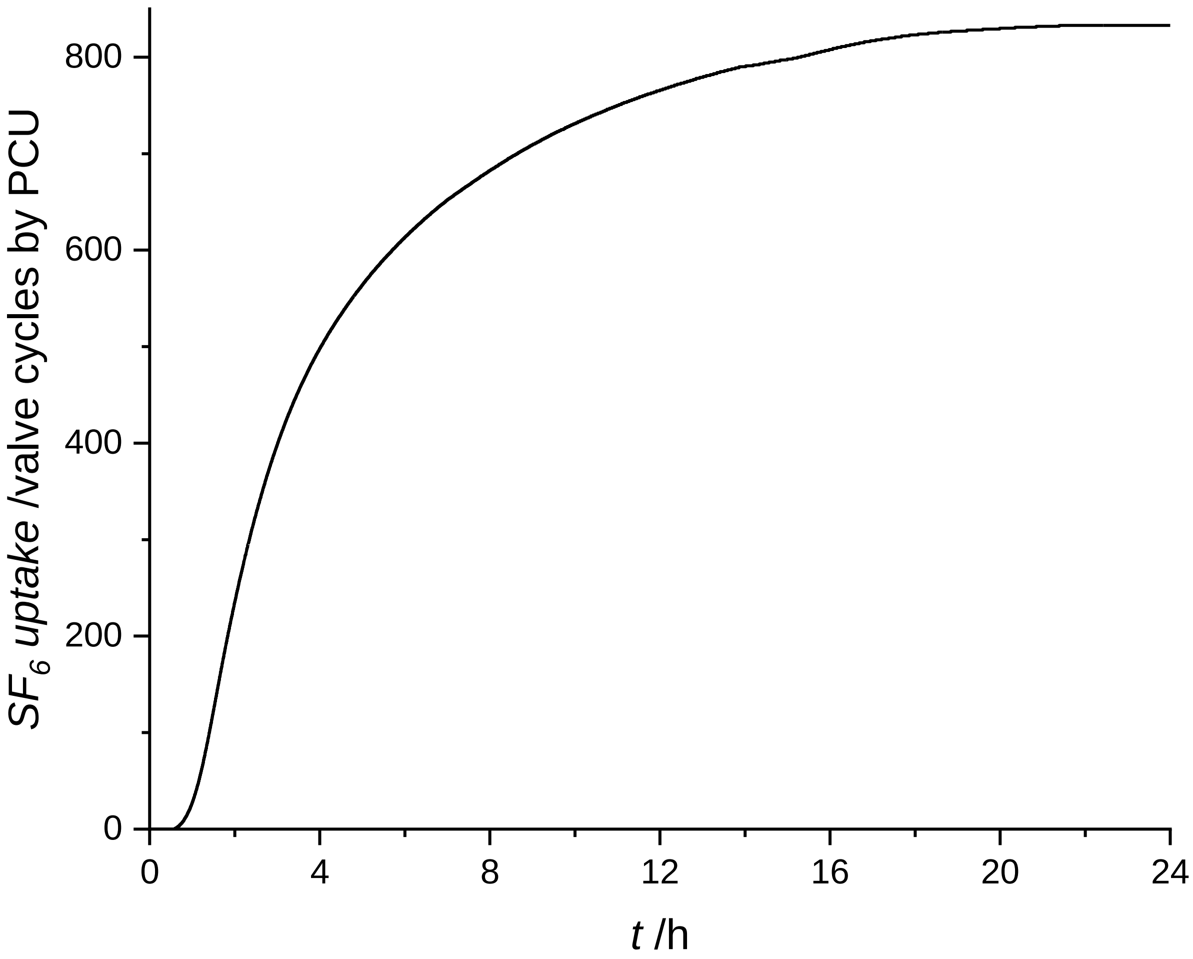

Figure S37: SF_6 uptake time plot for the KOH/iPrOH system (**GP4**) obtained by the PCU.

7 Photochemical Degradation of SF_6 in the biphasic KOH/H_2O/iPrOH System

7.1 Irradiation Setup 2

The experiment was performed using Irradiation Setup 2 following **GP2** using a mixture of water (2 ml) and HPLC grade isopropanol (13 ml) as solvent (KOH: 1.0 g, 16.8 mmol; SF_6 pressure: 2bar; irradiation time: 2 h; see chapter 4.2). The resulting biphasic system consists of an isopropanol layer on top of the concentrated aqueous solution of KOH. In contrast to the KOH/iPrOH system, no precipitate was formed during the reaction (Figure S38). The volatiles were distilled off at ambient temperature *in vacuo* for 4 hours and they were analysed by GC-MS and ^{1}H NMR spectroscopy. The remaining solid was analysed by ^{19}F qNMR, capillary electrophoresis and XRD. See Chapter 6.1.1 and 6.1.2 for a description of the characterization methods. Note that no thiosulfate was detected by CE.

Table S20: Overview of the analysis results of the products from the SF_6 decomposition reaction using the biphasic KOH/H_2O/iPrOH system according to **GP2**. (- = not detectible with the method, ✓ = detected, but not quantified)

Component	GC-MS	^{1}H NMR (mmol)	^{19}F qNMR (mmol)	CE (mmol)	XRD (mmol)
F^-	-	-	14.1	✓	13.6
SO_3^{2-}	-	-	-	✓	1.45
Diisopropyl sulfite	✓	0.84	-	-	-
Acetone	-	2.31	-	-	-

Following the method described in chapter 6.1.3, the experimental data in Table S20 and the overall reaction equation 13 were used to determine the yield. Yield: >99%.

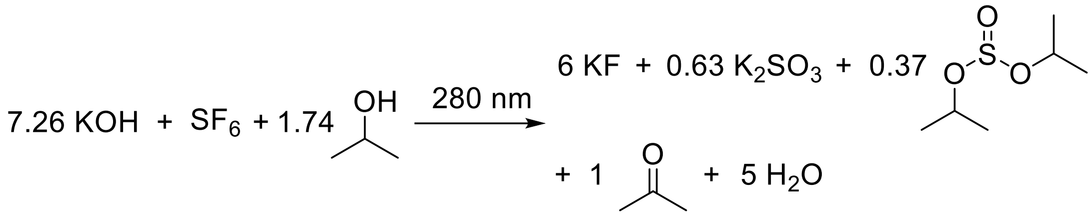

(13.)

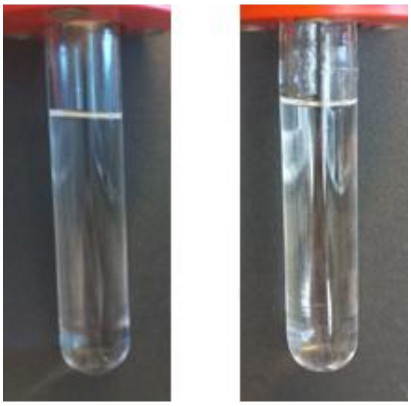

Figure S38: Initial biphasic KOH/H_2O/iPrOH system (left) and the reaction mixture after irradiation for 2 h (right).

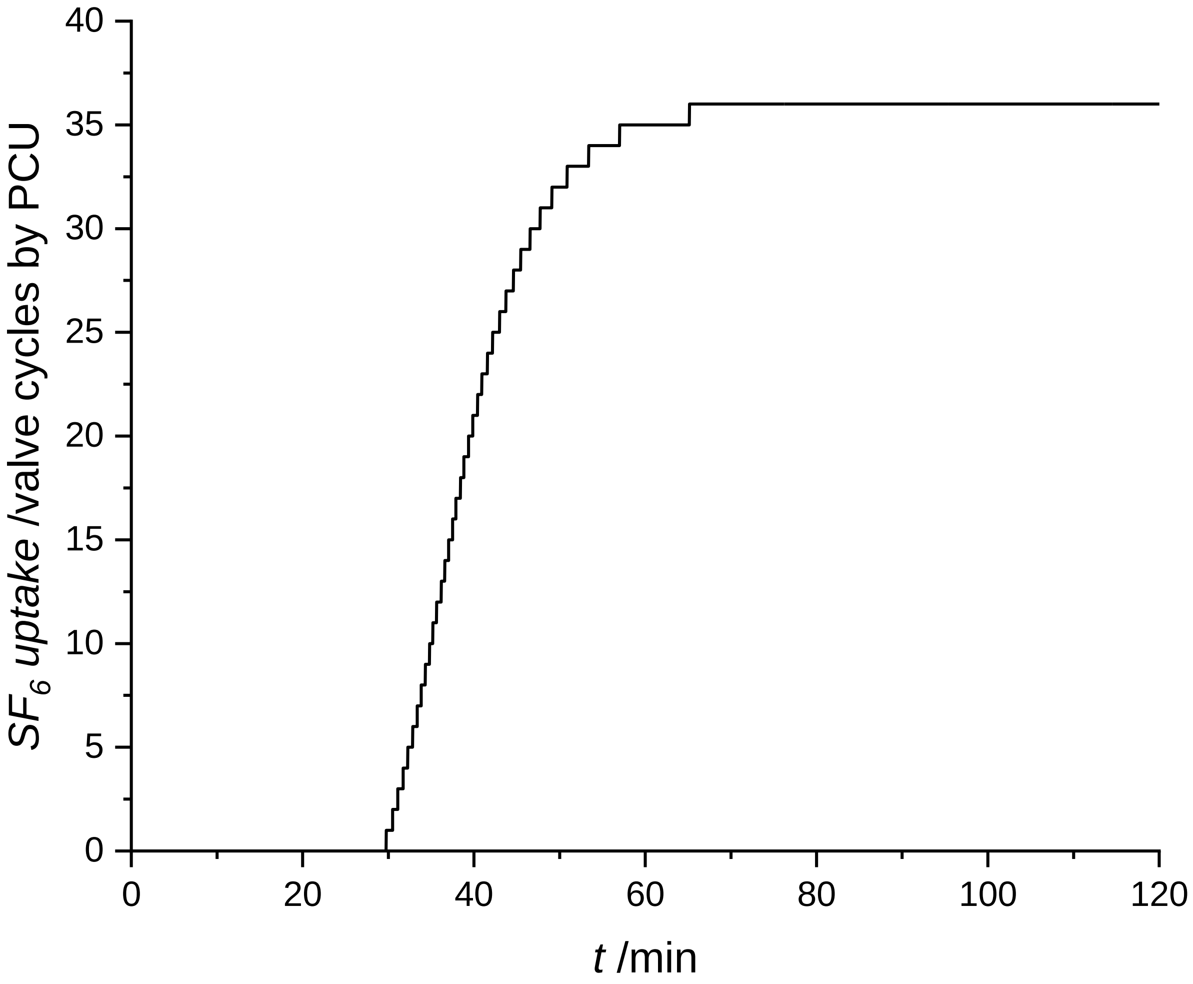

Figure S39: SF_6 uptake time plot for the biphasic KOH/H_2O/iPrOH system (**GP2**) obtained by the PCU.

7.2 Irradiation Setup 4

The experiment was performed using Irradiation Setup 4 following **GP4** using a mixture of water (100 ml) and technical grade isopropanol (750 ml) as solvent (KOH: 60 g, 1.0 mol; SF_6 pressure: 2 bar; irradiation time 12 h; see chapter 4.4). A biphasic system is formed consisting of an upper iPrOH layer and a lower aqueous KOH solution. Vigorous stirring affords an emulsion (Figure S40), which was irradiated for 12 hours. The resulting two phases were separated in a separation funnel. The iPrOH phase was analysed by GC-MS and ^{1}H NMR spectroscopy. An aliquot of 1 ml of the aqueous phase (134 g, 92.4 g/ml) was analysed by ^{19}F qNMR and capillary electrophoresis. See Chapter 6.1.1 and 6.1.2 for a description of the characterization methods.

Table S21: Overview of the analysis results of the products from the SF_6 decomposition reaction using the biphasic KOH/H_2O/iPrOH system according to **GP4**. (- = not detectible by the method, ✓ = detected, but not quantified)

Component	GC-MS	^{1}H NMR (mmol)	^{19}F qNMR (mmol)	CE (mmol)
F^-	-	-	781	✓
SO_3^{2-}	-	-	-	✓
Diisopropyl sulfite	✓	39	-	-
Acetone	-	130	-	-

Following the method described in chapter 6.1.3, , the experimental data in Table S21 and the overall reaction equation 14 was used to determine the yield. Yield: 96%.

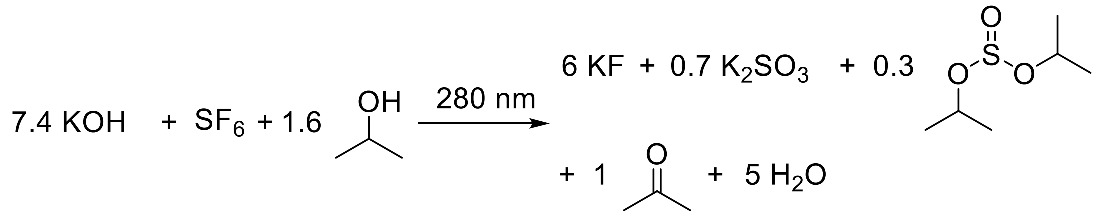

(14.)

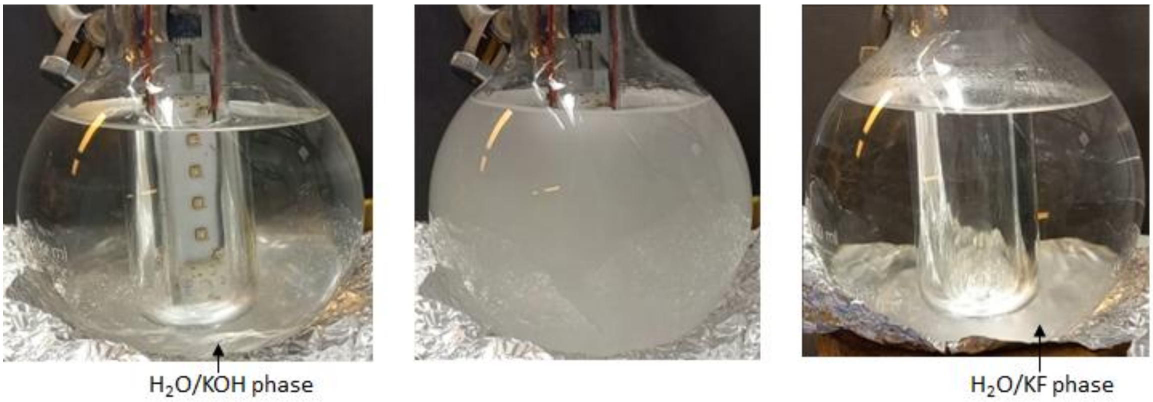

Figure S40: Initial biphasic KOH/H_2O/iPrOH system (left), generated emulsion upon stirring (center), biphasic reaction mixture after irradiation for 12 h (right).

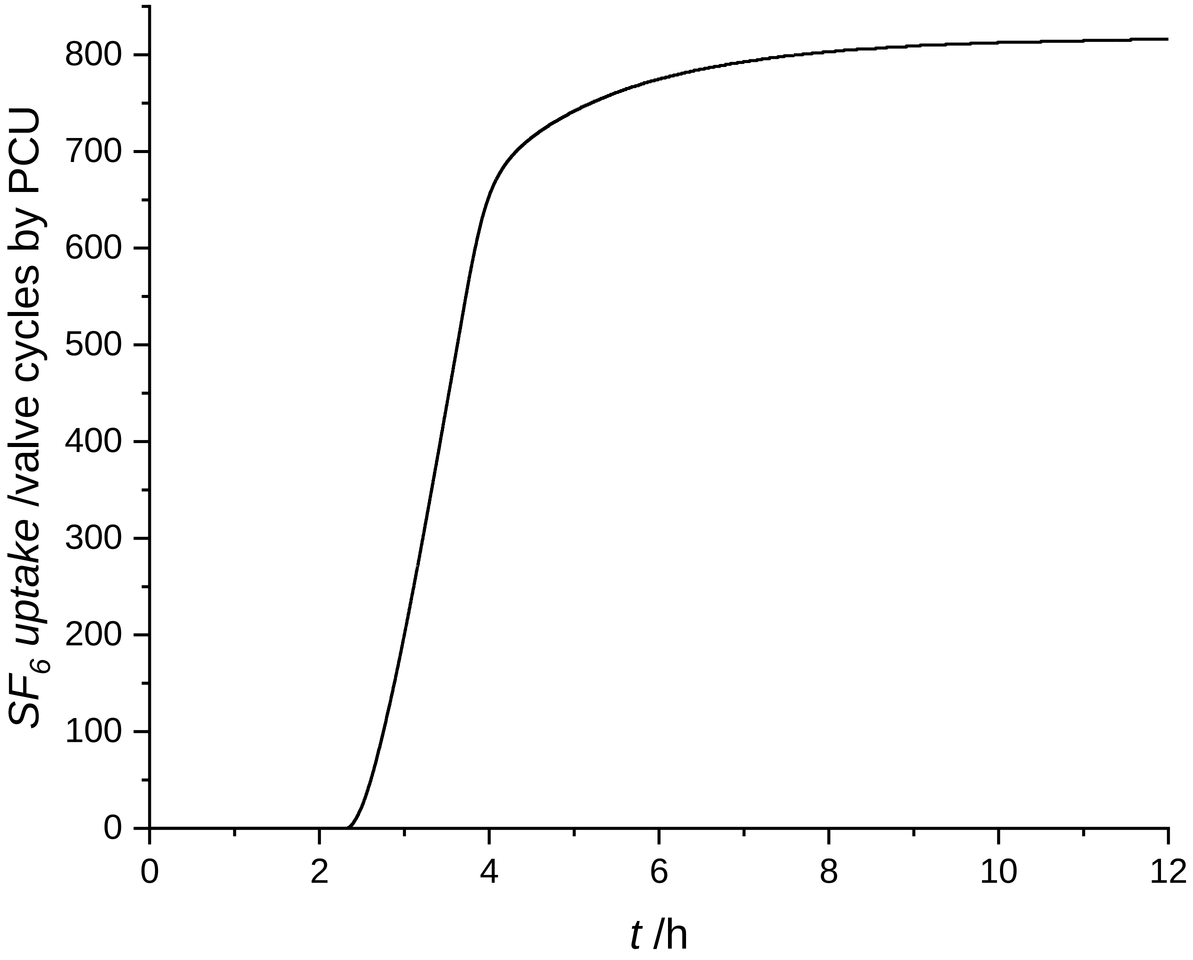

Figure S41: SF_6 uptake time plot for the biphasic KOH/H_2O/iPrOH system (**GP4**) obtained by the PCU.

8 Mechanistic Investigations

8.1 Influence of additives on the SF_6 degradation rate in the KOH/EtOH system

During the solvent screening (see Chapter 5.4) significantly different SF_6 degradation rates were observed for technical grade ethanol and HPLC grade ethanol. Technical ethanol contains about 1%V/V of isopropanol and of butanone. Therefore, isopropanol and butanone were used as additives in the photochemical degradation of SF_6 in HPLC grade ethanol (15 ml) following **GP2** (KOH: 1.0 g, 16.8 mmol; SF_6 pressure: 2 bar; irradiation time: 2 h; see chapter 4.2). Monitoring of the SF_6 uptake using the PCU (Figure S42) and the determination of the reaction yield (Table S22) reveals that isopropanol has little influence on the reaction rate, but the presence of butanone significantly accelerates the SF_6 degradation.

Table S22: Estimated yields based on fluoride formation (determined by ^{19}F qNMR spectroscopy) assuming KOH conversion according to the following reaction equation: 8 KOH + SF_6 + EtOH → 6 KF + K_2SO_3 + acetaldehyde + 5 H_2O.

Solvent	Additive	Yield (%)
ethanol (tech.)	-	29
ethanol (abs.)	-	1
ethanol (abs.)	isopropanol (0.75 ml)	4
ethanol (abs.)	butanone (0.75 ml)	51

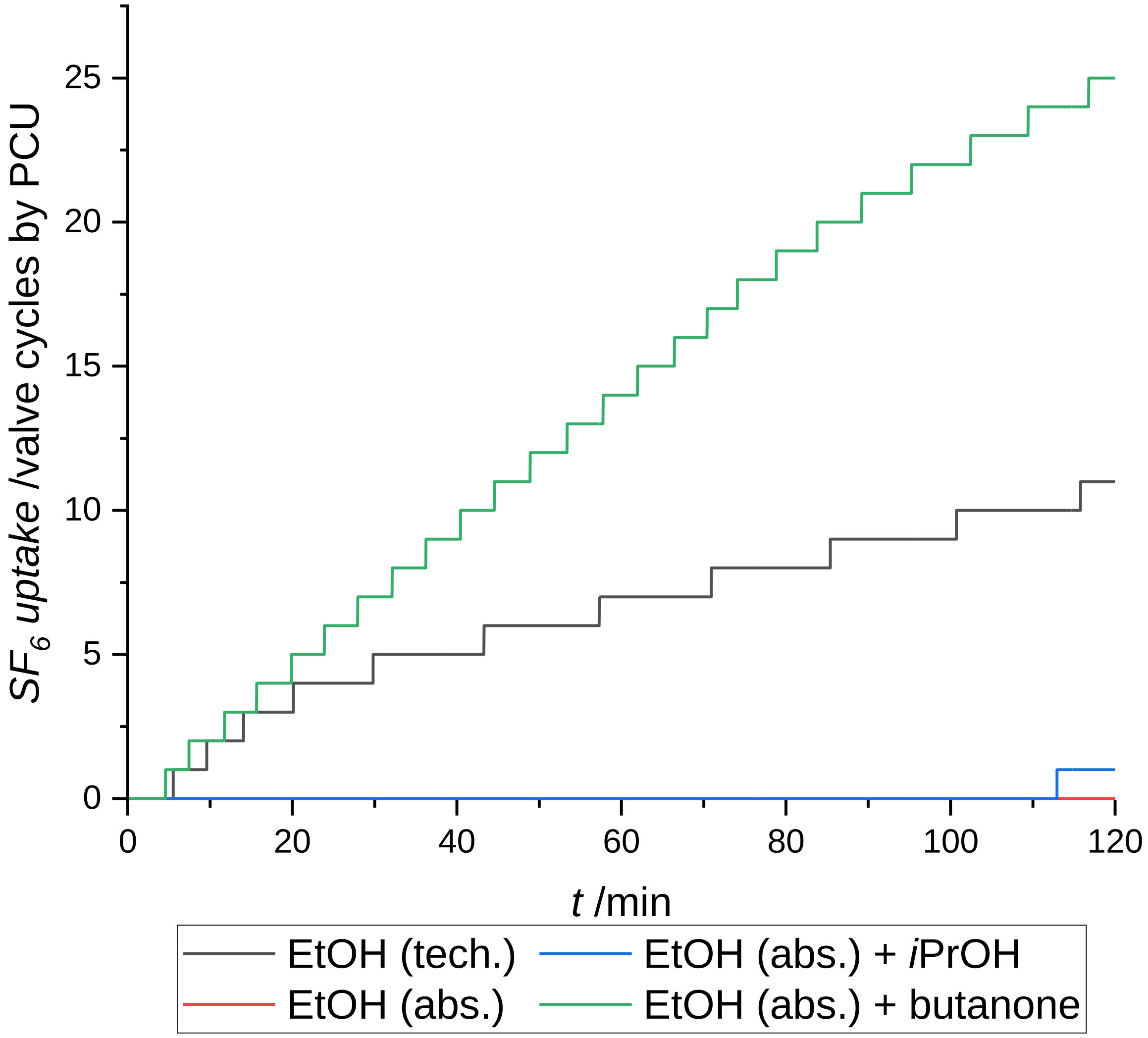

Figure S42: SF_6 uptake time plot for the KOH/EtOH system (**GP2**) obtained by the PCU showing the influence of additives on the SF_6 degradation rate.

8.2 Influence of acetone on the SF_6 degradation rate in the biphasic KOH/H_2O/iPrOH system

8.2.1 Irradiation Setup 2

The photochemical decomposition of SF_6 was carried out according to **GP2** using water (2 ml) and HPLC grade isopropanol (13 ml) as solvent (KOH: 1.0 g, 16.8 mmol; SF_6 pressure: 2 bar; irradiation time: 2 h; see chapter 4.2). The same reaction was carried out by adding sub-stoichiometric amounts of acetone (0.02 ml, 0.3 mmol) to the reaction mixture prior to irradiation. Both reactions were monitored using the PCU (Figure S43). In the case of acetone addition, exposure of the reaction mixture with light at 280 nm immediately led to the consumption of SF_6, while the reaction starts 26 minutes later without acetone. The yield is 98% based on the reaction equation 13 in Chapter 7.1.

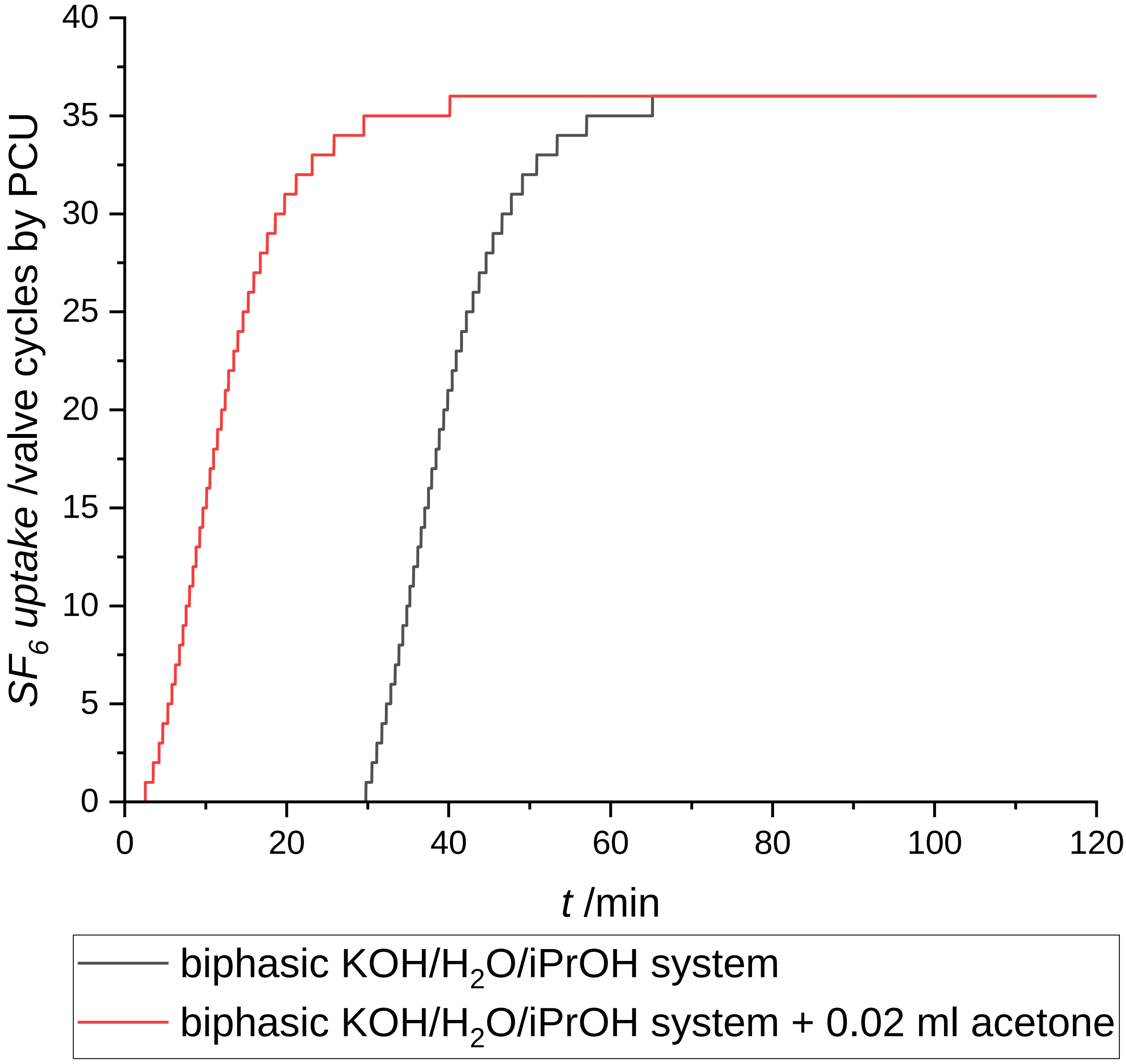

Figure S43: Uptake time plot obtained by the PCU (vide supra) of the biphasic KOH/H_2O/iPrOH system (**GP2**) with and without the addition of sub-stoichiometric amounts of acetone to the initial mixture.

8.2.2 Irradiation setup 4

The photochemical decomposition of SF_6 was carried out with the biphasic KOH/H_2O/iPrOH system according to **GP4** using water (100 ml) and technical grade isopropanol (750 ml) as solvent (KOH: 60 g, 1.0 mol; SF_6 pressure: 2 bar; irradiation time 12 h; see chapter 4.4). The same reaction was carried out by adding sub-stoichiometric amounts of acetone (0.1 ml, 1.3 mmol) to the reaction

mixture prior to irradiation. Both reactions were monitored using the PCU (Figure S44). Note that the reaction starts significantly earlier with acetone. However, compared to the 15 mL-scale reaction of Chapter 8.2.1, the initial reaction mixture was not degassed and contained air, which was shown to hamper the SF_6 degradation (see Chapter 6.2.1). In a second experiment sub-stoichiometric amounts of acetone (1 ml, 13 mmol) were added, and the reaction mixture was degassed by bubbling argon for 10 min prior to irradiation. An aliquot of 1 ml of the aqueous solution was analysed by quantitative ^{19}F NMR spectroscopy using potassium triflate (10.0 mg, 0.05 mmol) as internal standard. The yields are 98% and 99% for the first and second experiment, respectively, based on the reaction equation 14 in Chapter 7.2.

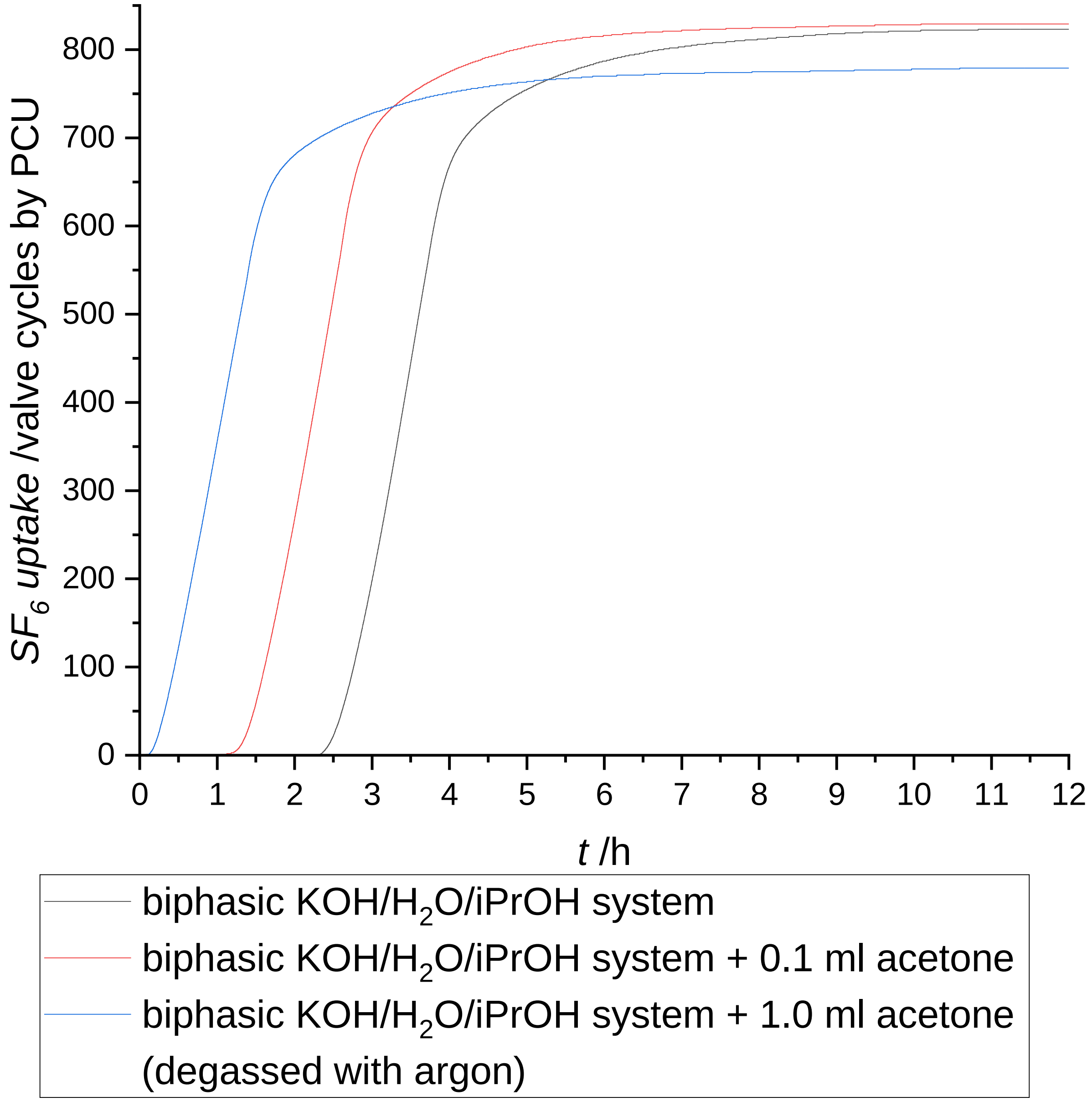

Figure S44: Uptake time plot obtained by the PCU (vide supra) of the biphasic $KOH/H_2O/iPrOH$ system (**GP4**) with and without the addition of sub-stoichiometric amounts of acetone to the initial mixture and degassing by argon. Note that the higher overall number of valve cycles when starting with an air atmosphere is due to the consumption of oxygen under the applied reaction conditions by the reaction mixture.

8.3 UV–vis absorption spectroscopy

A standard quartz UV-vis cuvette (d = 1 cm) was charged with a solution of potassium hydroxide (90 mg, 1.6 mmol) in an alcohol (3 mL), pressurized with SF_6 (3 bar) and irradiated with light at 280 nm for 1 hour using irradiation setup 2. UV-vis spectra were recorded of the alcohol, of the initial reaction mixture (alcohol+KOH+SF_6), and of the reaction mixture after irradiation. If solids were formed during the reaction, the suspension was first filtrated and then a UV-vis spectrum was recorded of the clear solution. The obtained solution after irradiation was additionally analyzed by GC-MS using the established procedure (see chapter 3.2), but apart from the solvent and the organosulfites in the case of isopropanol and sec-butanol no other organic compounds were detected.

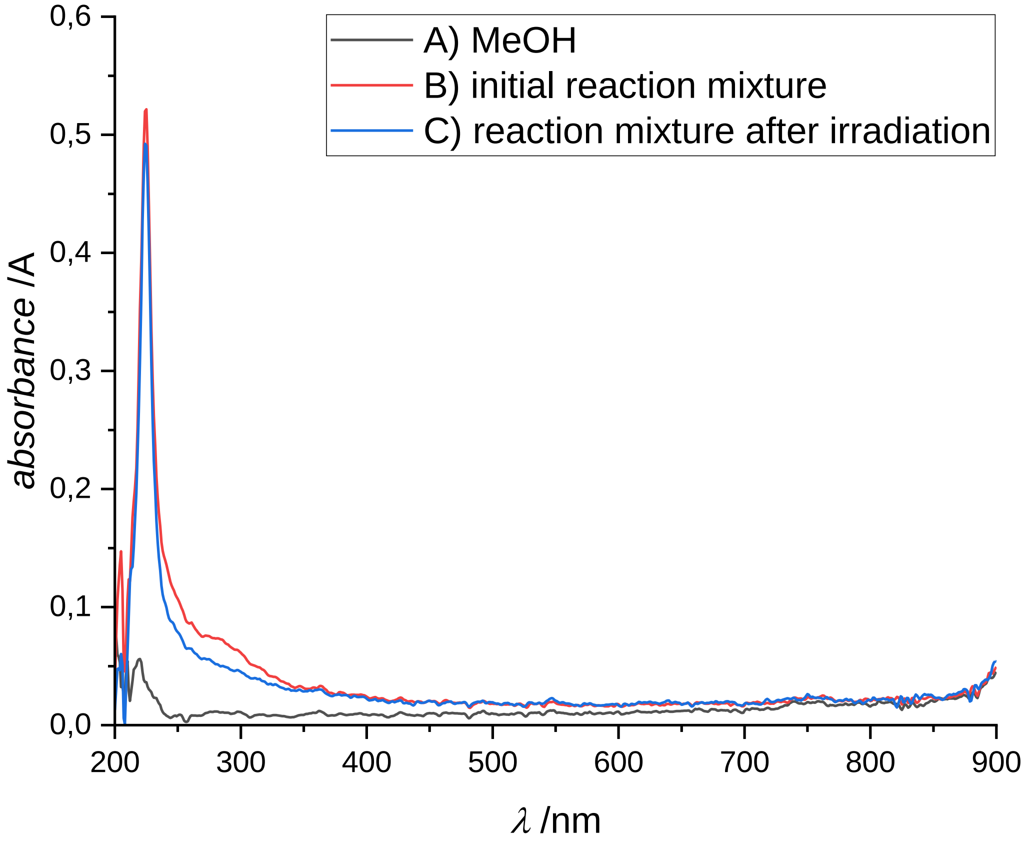

Figure S45: UV-vis spectra of the KOH/MeOH system. A) MeOH, B) The initial KOH/MeOH solution, C) The reaction mixture after irradiation with light at 280 nm for 1 hour.

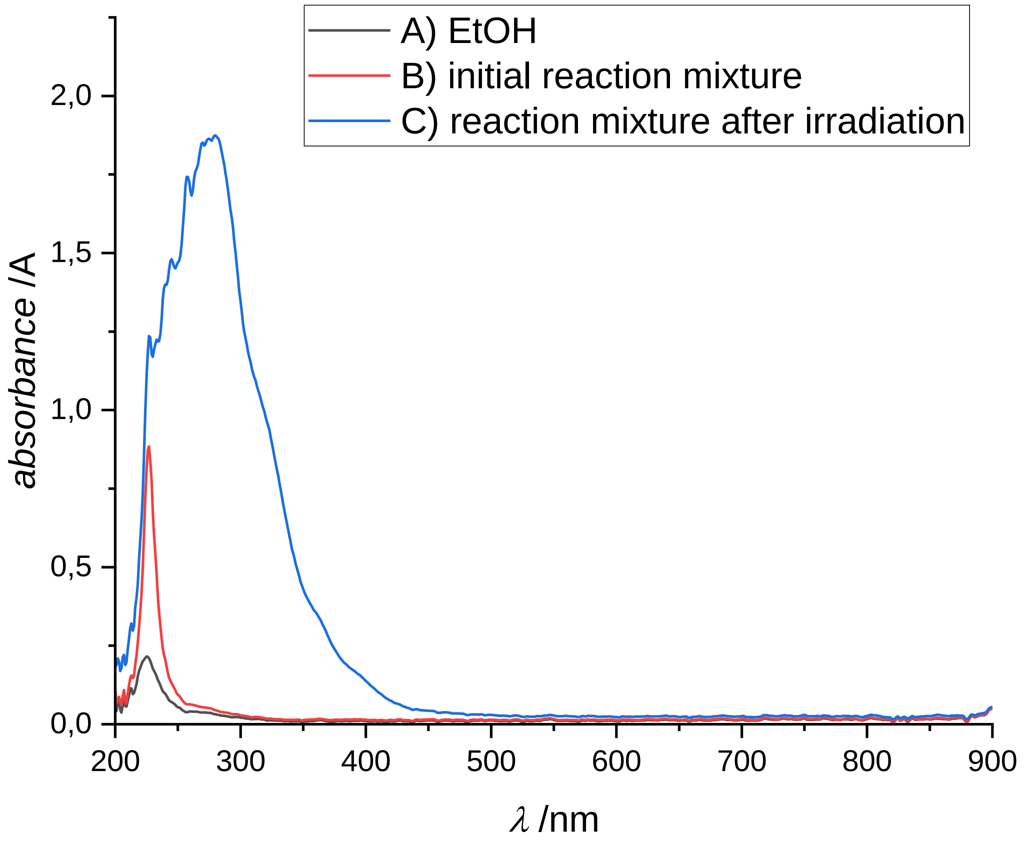

Figure S46: UV-vis spectra of the KOH/EtOH system. A) EtOH, B) The initial KOH/EtOH solution, C) The reaction mixture after irradiation with light at 280 nm for 1 hour.

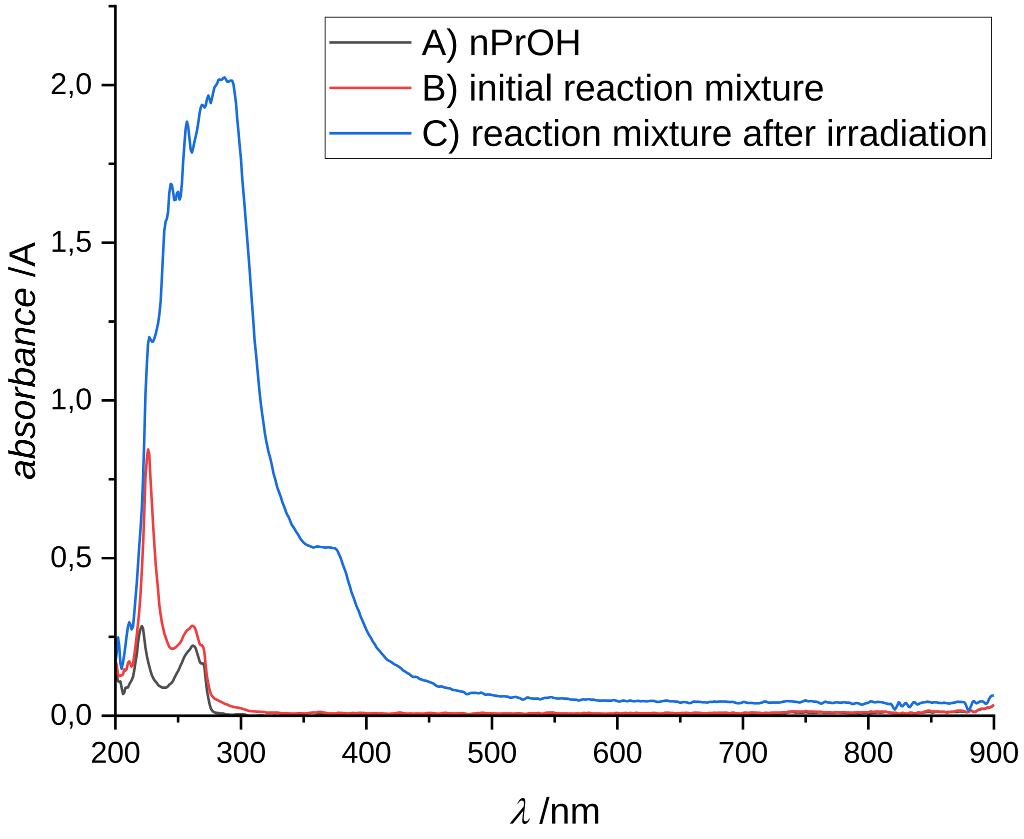

Figure S47: UV-vis spectra of the KOH/nPrOH system. A) nPrOH, B) The initial KOH/nPrOH solution, C) The reaction mixture after irradiation with light at 280 nm for 1 hour.

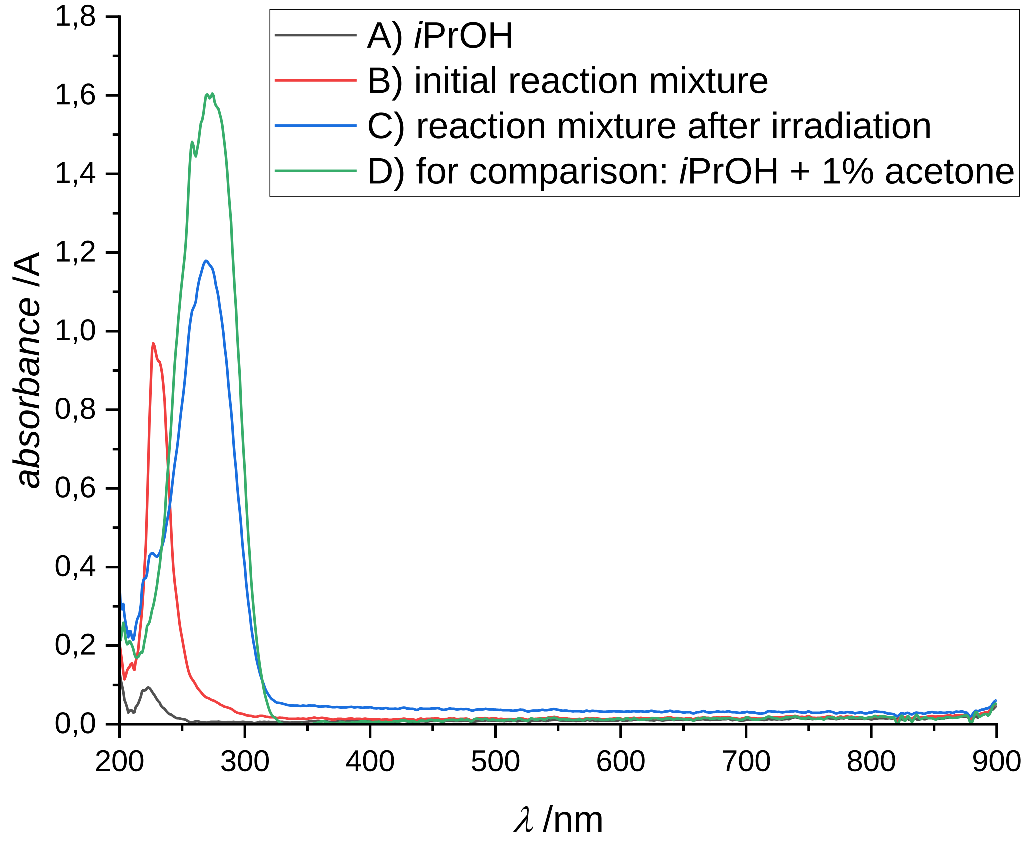

Figure S48: UV-vis spectra of the KOH/iPrOH system. A) HPLC grade iPrOH, B) The initial KOH/iPrOH solution, C) The reaction suspension after irradiation with light at 280 nm for 1 hour, D) HPLC grade iPrOH with 1% acetone.

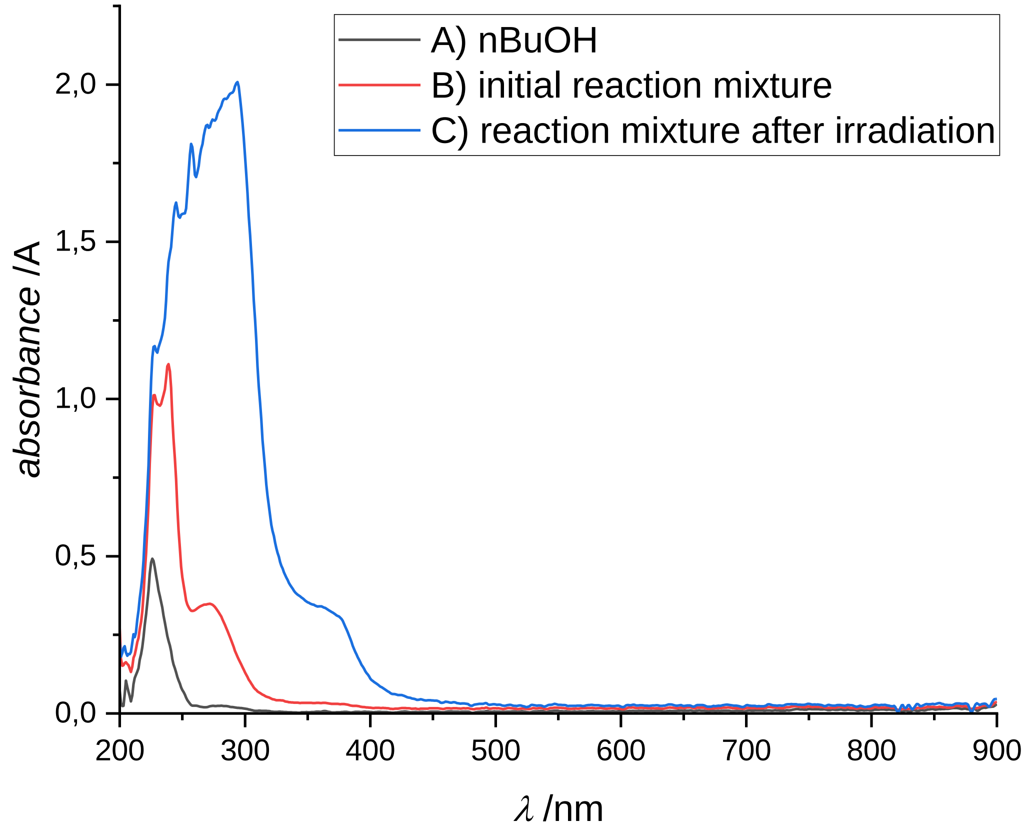

Figure S49: UV-vis spectra of the KOH/nBuOH system. A) nBuOH, B) The initial KOH/nBuOH solution, C) The reaction mixture after irradiation with light at 280 nm for 1 hour.

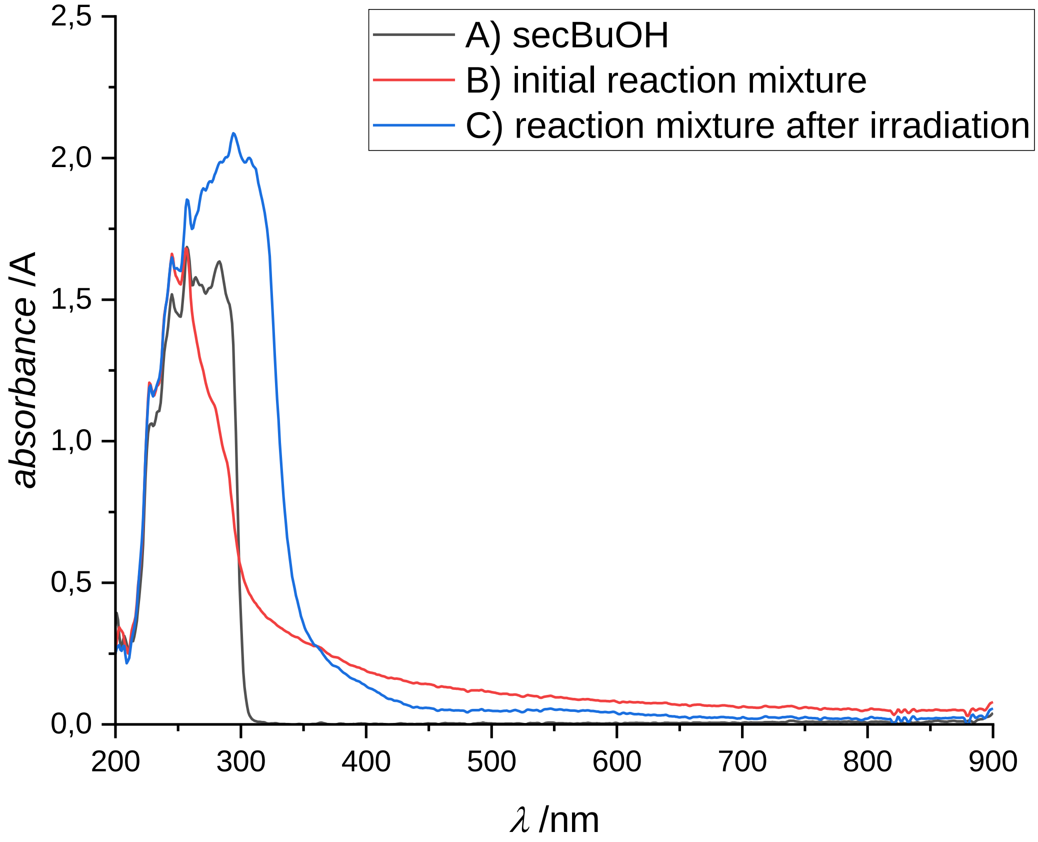

Figure S50: UV-vis spectra of the KOH/secBuOH system. A) secBuOH, B) The initial KOH/secBuOH solution, C) The reaction mixture after irradiation with light at 280 nm for 1 hour.

8.4 EPR spectroscopy

Description of the experiment: A standard quartz EPR tube was charged with different mixtures (see below). The EPR experiment was carried out at -50°C and by irradiation of the tube using a mercury xenon lamp (**250-550 nm**, The emission spectrum of the lamp is depicted in chapter 3.8). The following mixtures were analysed by EPR spectroscopy:

A) A degassed solution of potassium hydroxide (30 mg) and acetone (1 drop) in HPLC grade isopropanol (1 ml) under argon atmosphere.
B) The reaction mixture obtained after irradiation for 1 h at a wavelength of 280 nm using setup 1, starting with a degassed solution of potassium hydroxide (30 mg) in HPLC grade isopropanol (1 ml) under SF_6 atmosphere (3 bar).
C) A degassed solution of potassium hydroxide (30 mg) in HPLC grade isopropanol (1 ml) under SF_6 atmosphere (3 bar).
D) A degassed suspension of potassium sulfite (30 mg) in HPLC grade isopropanol (1 ml) under argon atmosphere.

Mixture A (Figure S51) shows the characteristic resonances of the ketyl radical anion, with g_{iso} = 2.0033 and a_{iso} = 54 MHz. In mixture B (Figure S52) the signal for the ketyl radical is observed, but at significantly lower intensity and broader peaks. We attribute this to the lack of KOH to deprotonate the radical, resulting in additional hyperfine coupling to the OH proton. Unfortunately, this could not be spectroscopically resolved in our experiments. In mixture C (Figure S53) no defined EPR Signal could by isolated at the given conditions (e.g. slow diffusion-controlled environment). The observed signal is considerably lower in intensity than mixtures A and B, indicating fast reaction once the radicals are formed. Mixture D (Figure S54) was measured to exclude the possibility of sulfite radicals being detected.

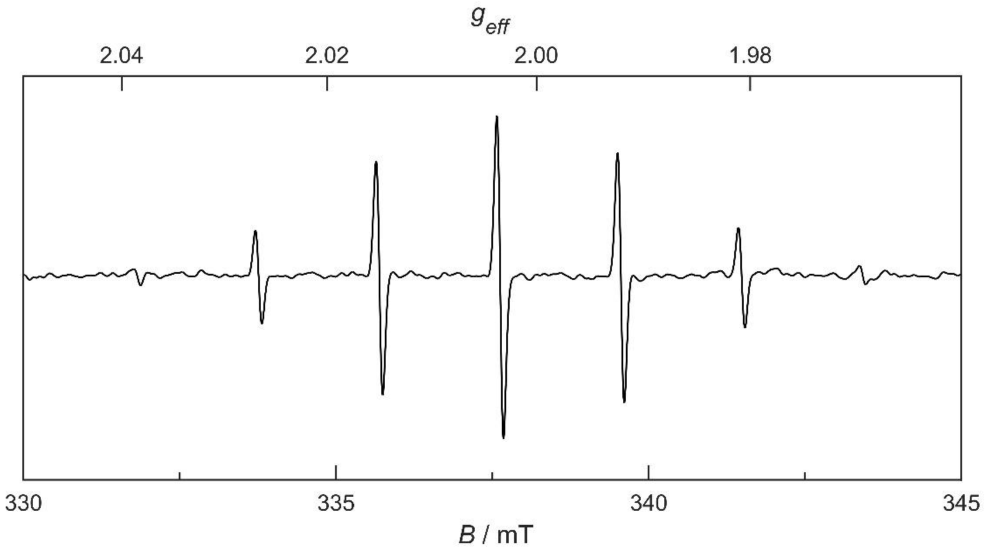

Figure S51: EPR spectrum of mixture A (potassium hydroxide and acetone in HPLC grade isopropanol).

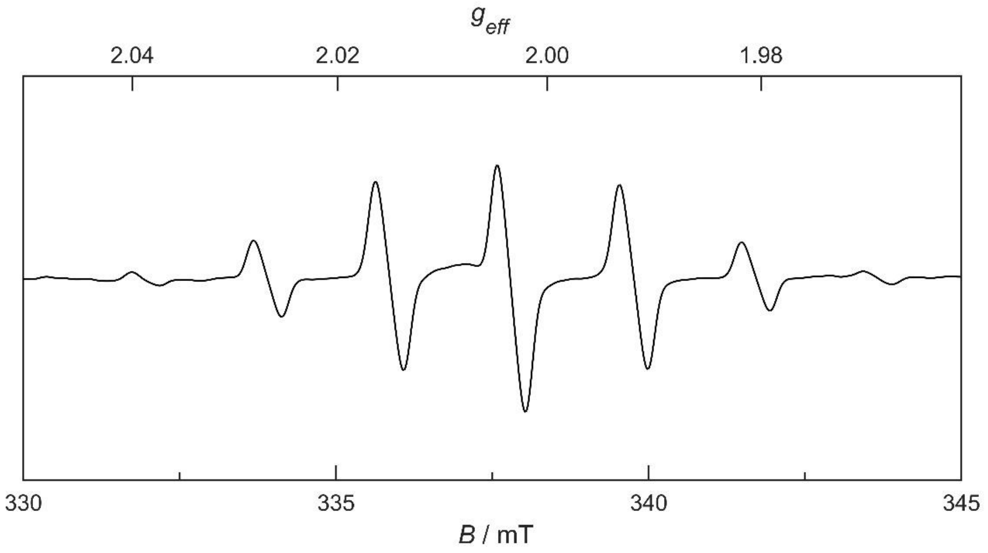

Figure S52: EPR spectrum of mixture B (reaction mixture after prior irradiation at 280 nm for 1 h).

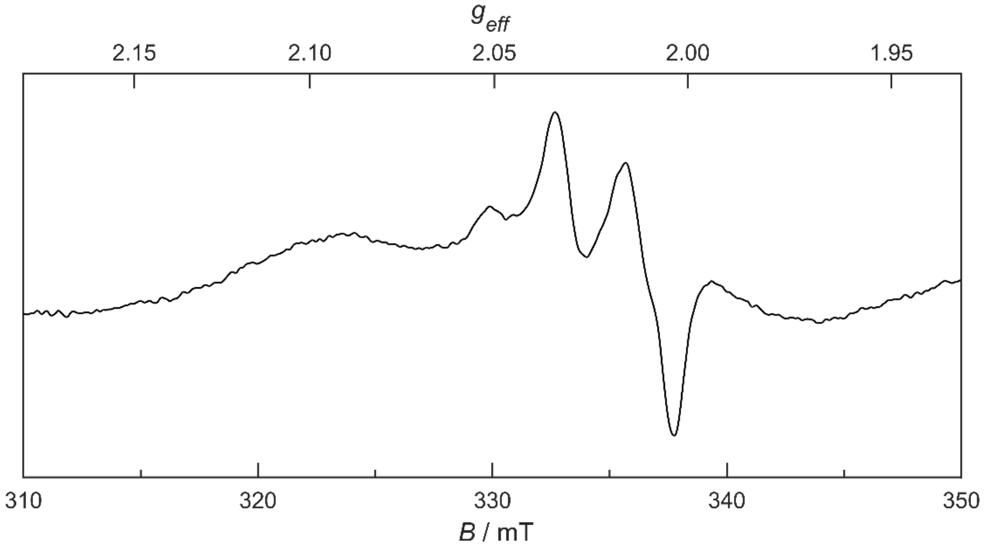

Figure S53: EPR spectrum of mixture C (reaction mixture during irradiation with the mercury xenon lamp).

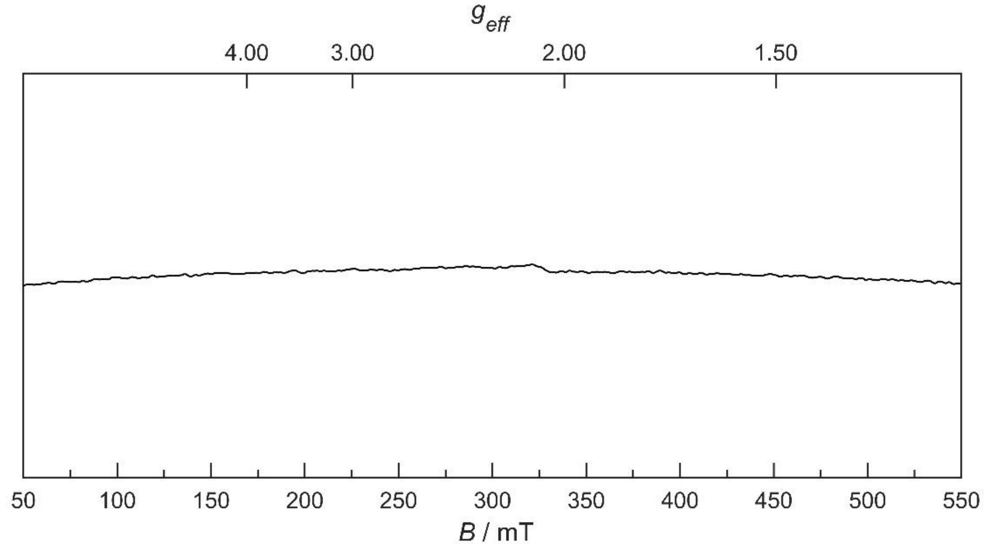

Figure S54: EPR spectrum of mixture D (potassium sulfite suspended in HPLC grade isopropanol).

8.5 Influence of diisopropyl sulfite

8.5.1 Hydrolysis of diisopropyl sulfite by excess of potassium hydroxide

To a potassium hydroxide (0.34 g, 5.71 mmol, 9.5 eq.) solution in HPLC grade isopropanol (5 ml) diisopropyl sulfite (0.1 ml, 0.60 mmol, 1.0 eq.) was added and stirred for 3 h. Immediately a white precipitate (K_2SO_3) was observed (Figure S55). At the start, after 1 h and 3 h a GC-MS sample was taken and measured (see chapter 3.2). After 1 h diisopropyl sulfite can barely be detected in small quantities and after 3 h no diisopropyl sulfite was detected in the GC-MS.

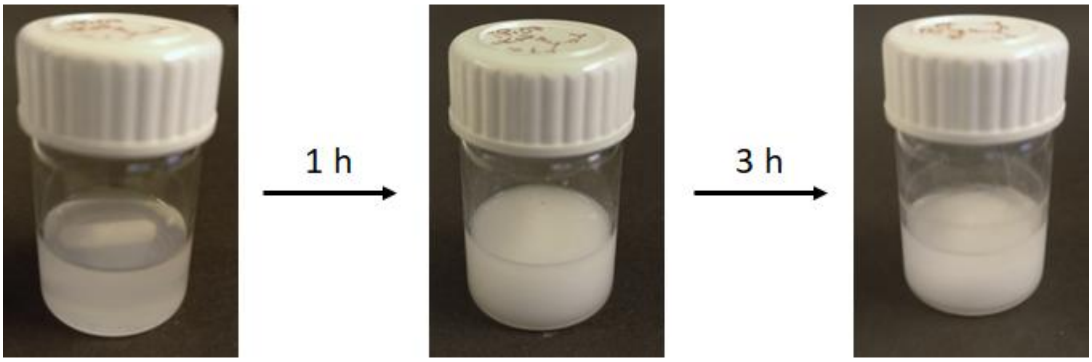

Figure S55: Hydrolysis of diisopropyl sulfite in excess of potassium hydroxide. Right after the start (left), after 1 h (middle) and after 3 h (right).

8.5.2 Influence of diisopropyl sulfite on the SF_6 degradation rate in the KOH/iPrOH system

The photochemical decomposition of SF_6 was carried out according to **GP2** using HPLC grade isopropanol (15 ml) as solvent (KOH: 1.0 g, 16.8 mmol; SF_6 pressure: 2 bar; irradiation time: 2 h; see chapter 4.2) and diisopropyl sulfite (0.3 ml, 1.8 mmol) was added to the reaction mixture prior to irradiation. A white precipitate was formed, due to the hydrolysis of diisopropylsulfite into potassium sulfite in the presence of potassium hydroxide. The reaction was monitored using the PCU (Figure S56). The yield is 98% based on the reaction equation 13 in Chapter7.1.

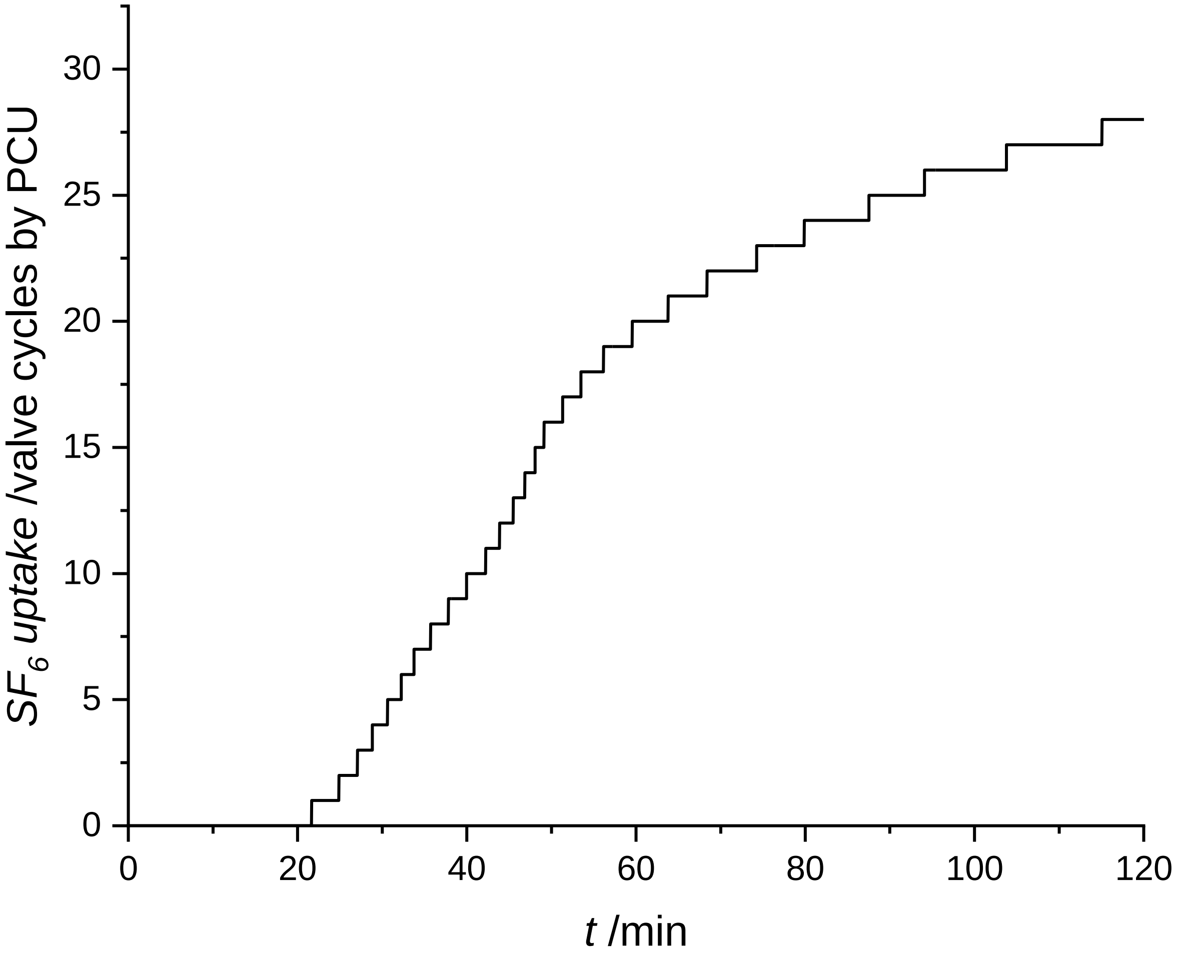

Figure S56: SF_6 uptake time plot for the KOH/iPrOH system (**GP2**) obtained by the PCU showing the influence of addition of diisopropyl sulfite on the reaction rate.

8.6 Investigating the stability of acetone in the KOH/iPrOH and the KOH/H_2O/iPrOH system

Potassium hydroxide (340 mg, 5.7 mmol) was dissolved in HPLC grade isopropanol (5 ml) (KOH/iPrOH system) or in HPLC grade isopropanol (4.4 ml) and water (0.6 ml) (KOH/H_2O/iPrOH system). Afterwards acetone (0.1 ml, 1.3 mmol) was added to both mixture and the mixtures were stirred for 24 h at room temperature. The KOH/iPrOH system changed colour from yellow (3 h) to red/brown (24 h) whereas the KOH/H_2O/iPrOH system remained colourless. After 24 h the organic phases of both mixtures were analysed by ^1H NMR (Figure S57), GC-MS and UV-vis (Figure S58) after filtrating over silica. In the case of the KOH/H_2O/iPrOH system no change in the ^1H NMR spectrum was observed, and acetone is still present. In the GC-MS no additional peaks were observed, and the UV-vis spectra is similar to the UV-vis spectrum of a 1% solution of acetone in isopropanol. In the case of the KOH/iPrOH system in the ^1H NMR spectrum the acetone is almost completely consumed, and many new signals have emerged. In the GC-MS no additional peaks were observed but the UV-vis shows a much broader and higher absorption than the UV vis spectrum of a 1% solution of acetone in isopropanol. The products were not further characterized.

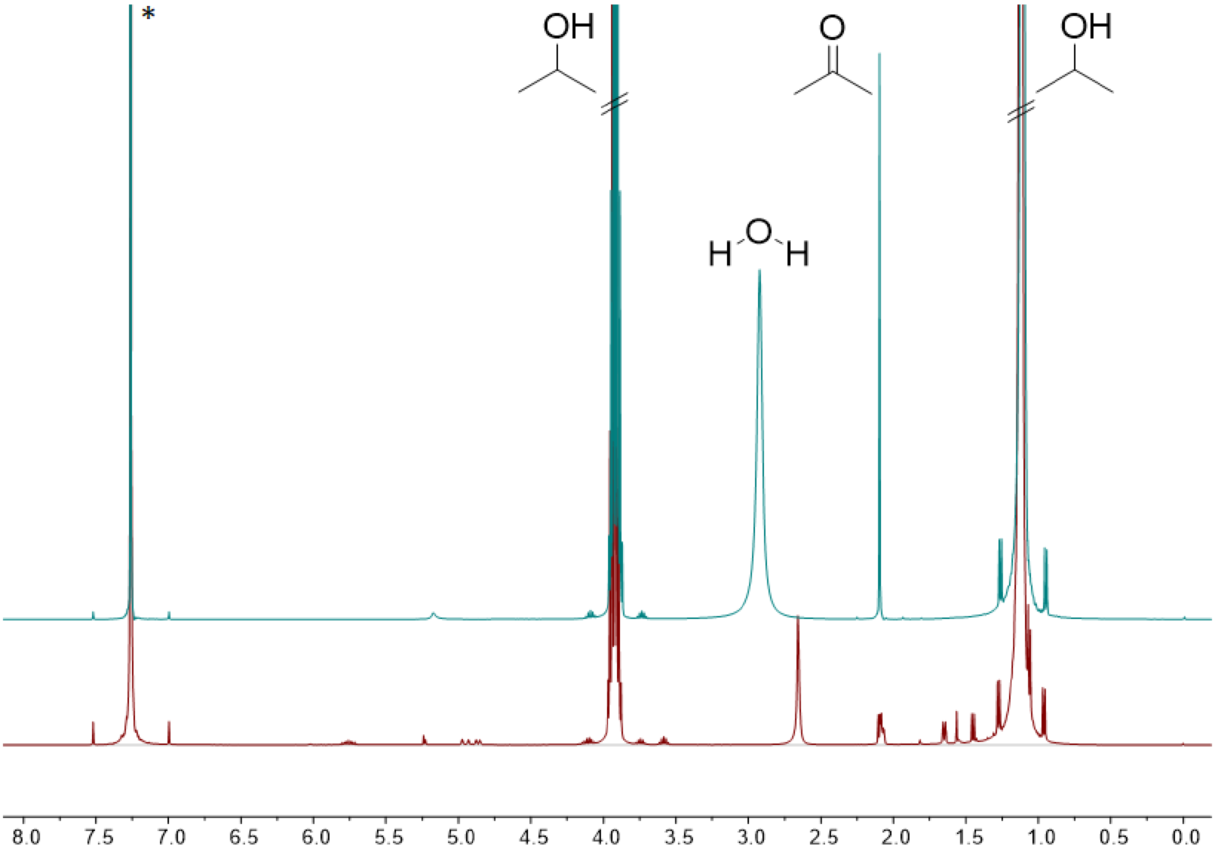

Figure S57: Stacked ^1H NMR spectra of the acetone base stability experiment in the KOH/iPrOH system (red) and the KOH/H_2O/iPrOH system (blue).*$CDCl_3$

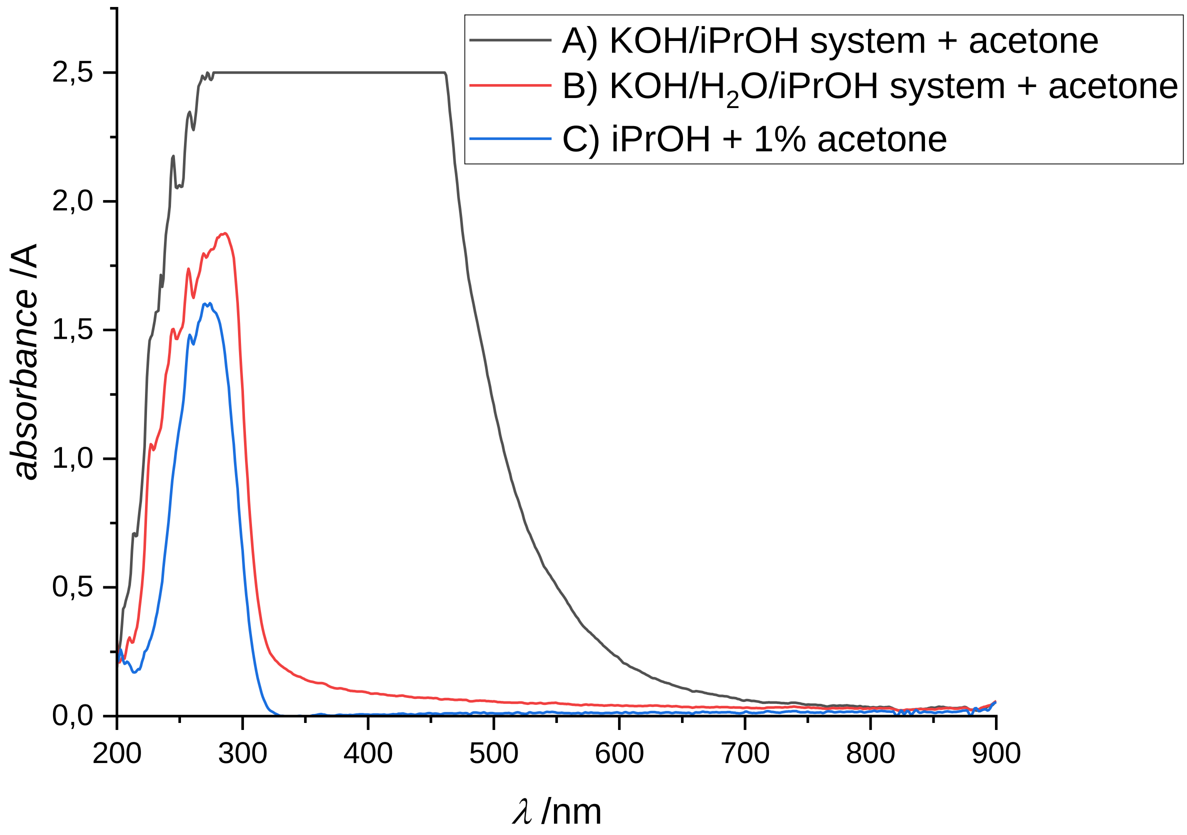

Figure S58: UV-vis spectra of the acetone base stability experiment. A) after 24 h in the KOH/iPrOH system, B) after 24 h in the KOH/H_2O/iPrOH system, C) HPLC grade isopropanol with 1% acetone.

8.7 The use of di-*tert*-butyl peroxide as radical initiator

A PTFE sealed pressure flask was charged with a solution of potassium hydroxide (1.0 g, 16.8 mmol) in HPLC grade isopropanol (15 ml) (**A**) or with a solution of potassium hydroxide (1.0 g, 16.8 mmol) and di-*tert*-butyl peroxide (DTBP) (0.1 ml, 0.54 mmol) in HPLC grade isopropanol (15 ml) (**B**). The solutions were degassed by three freeze-pump-thaw cycles and the pressure flask was pressurized with SF_6 (2 bar). Both reaction mixtures were heated up to 100°C and stirred for 24 h. Afterwards the volatiles were evaporated under reduced pressure to afford a mixture of inorganic salts (e.g. KF, K_2SO_3). The residue was dissolved in water until a volume of 5 mL was obtained. An aliquot of 1 ml of this aqueous solution was analysed by quantitative ^{19}F NMR spectroscopy using potassium triflate (10.0 mg, 0.053 mmol) as internal standard. Assignment of the ^{19}F NMR resonances: δ = −78.6 (OTf^-), −119.1 (F^-) ppm.

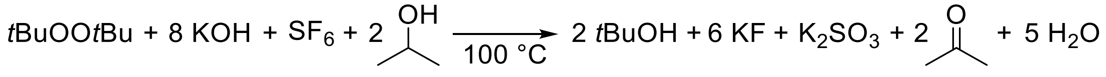

(15.)

Yield without DTBP (**A**): 0.2 mmol KF (2%).

Yield with DTBP (**B**): 7.8 mmol KF (62% based on KOH).

Theoretical maximum yield of KF (based on DTBP) for the stoichiometric reaction between DTBP and SF_6 according to the equation 15 above: 3.2 mmol KF.

8.8 Attempted generation of the dimethyl ketyl radical anion under anhydrous conditions and in-situ reaction with SF_6

An NMR tube was charged with potassium metal (21.0 mg, 0.53 mmol) and THF (1 mL). Upon adding anhydrous acetone (0.04 mL, 0.53 mmol, dried over 3 Å molecular sieves), gas evolution was observed at the metal surface, and the THF solution turned slightly turbid, likely due to trace water in the acetone or slow decomposition of acetone. After 10 minutes, with potassium metal still present, the NMR tube was pressurized with 3 bar SF_6. This led to a color change from yellow to orange, more solid formation and the complete consumption of potassium. In the ^{19}F NMR spectrum, the signal of dissolved SF_6 (δ = 57.3 ppm) was detected as only fluorine containing compound. The volatiles were evaporated, and the resulting solid was dissolved in water. Quantitative ^{19}F NMR spectroscopy, using potassium triflate (10.0 mg, 0.05 mmol) as an internal standard (^{19}F NMR resonances: δ = −78.6 (OTf^-), −119.1 (F^-) ppm) revealed the formation of fluoride: 3.5 mg (0.06 mmol).

In a control experiment, an NMR tube containing only potassium metal (21.0 mg, 0.53 mmol) and THF (1 mL) was pressurized with 3 bar SF_6. No visible reaction occurred after 1 day, and only SF_6 was detected in the ^{19}F NMR spectrum (δ = 57.3 ppm).

These results suggest that the in-situ generated species, likely the dimethyl ketyl radical anion, is capable of activating SF_6, whereas potassium metal alone does not react with SF_6 under the same conditions.

8.9 Estimation of the quantum efficiency

The quantum efficiency (Φ) of the SF_6 degradation process is calculated using the following equation:

$$\Phi = \frac{\dot{N}_{SF6}}{\dot{N}_{photons}} \qquad (16.)$$

Where $\dot{N}_{SF6}$ is the number of SF_6 molecules reacted per hour, and $\dot{N}_{photons}$ is the number of photons absorbed by the reaction mixture per hour.

Calculation of $\dot{N}_{SF6}$

The value of $\dot{N}_{SF6}$ is determined from the experimental degradation rate (*DR*, see Chapter 9) and the molar mass of SF_6 (*M*).

$$\dot{N}_{SF6} = \frac{DR}{M(SF_6)} \qquad (17.)$$

For the KOH/iPrOH system, with a degradation rate of 0.7 g/h and $M(SF_6)$ =146.06 g/mol:

$$\dot{N}_{SF6} = \frac{0.7\ \frac{\mathrm{g}}{\mathrm{h}}}{146.06\ \frac{\mathrm{g}}{\mathrm{mol}}} = 4.8\ \frac{\mathrm{mmol}}{\mathrm{h}} \qquad (18.)$$

For the KOH/H_2O/iPrOH system, with a degradation rate of 1.1 g/h:

$$\dot{N}_{SF6} = \frac{1.1\ \frac{\mathrm{g}}{\mathrm{h}}}{146.06\ \frac{\mathrm{g}}{\mathrm{mol}}} = 7.5\ \frac{\mathrm{mmol}}{\mathrm{h}} \qquad (19.)$$

Calculation of $\dot{N}_{photons}$

The number of photons absorbed per hour, $\dot{N}_{photons}$, is calculated using the energy per photon (E_{photon}), the irradiation time (*t*), the irradiation power (P_{irr}) of the LED, and the Avogadro constant (N_A):[9,10]

$$E_{photons} = \frac{h \cdot c}{\lambda} \tag{20.}$$

$$\dot{N}_{photons} = \frac{P_{irr}(LED) \cdot t}{E_{photons} \cdot N_A} = \frac{P_{irr}(LED) \cdot t \cdot \lambda}{h \cdot c \cdot N_A} \tag{21.}$$

Using the following values:

- P_{irr} = 220 mW = 0.22 J/s
- t = 1 h = 3600 s/h
- λ = 280 nm = $2.8 \cdot 10^{-7}$ m (wavelength)
- h = $6.626 \cdot 10^{-34}$ Js (Planck's constant)
- c = $3 \cdot 10^{8}$ m/s (speed of light)
- N_A = $6.022 \cdot 10^{23}$ mol^{-1}

We calculate:

$$\dot{N}_{photons} = \frac{0.22\ \frac{\mathrm{J}}{\mathrm{s}} \cdot 3600\ \frac{\mathrm{s}}{\mathrm{h}} \cdot 2.8 \cdot 10^{-7}\ \mathrm{m}}{6.626 \cdot 10^{-34}\ \mathrm{Js} \cdot 3 \cdot 10^{8}\ \frac{\mathrm{m}}{\mathrm{s}} \cdot 6.022 \cdot 10^{23}\ \mathrm{mol}^{-1}} = 1.8\ \frac{\mathrm{mmol}}{\mathrm{h}} \tag{22.}$$

It is important to note that $\dot{N}_{photons}$ represents an upper limit, as it assumes all emitted photons are absorbed by the reaction mixture without accounting for losses in the setup.

Calculation of Minimum Quantum Efficiency (Φ_{min})

With that, the minimum quantum efficiency of the KOH/iPrOH system is calculated as:

$$\Phi_{min} = \frac{\dot{N}_{SF6}}{\dot{N}_{photons}} = \frac{4.8\ \frac{\mathrm{mmol}}{\mathrm{h}}}{1.8\ \frac{\mathrm{mmol}}{\mathrm{h}}} = 2.7 \tag{23.}$$

The minimum quantum efficiency of the KOH/H_2O/iPrOH system is calculated as:

$$\Phi_{min} = \frac{\dot{N}_{SF6}}{\dot{N}_{photons}} = \frac{7.5\ \frac{\mathrm{mmol}}{\mathrm{h}}}{1.8\ \frac{\mathrm{mmol}}{\mathrm{h}}} = 4.2 \tag{24.}$$

The calculated minimum quantum efficiencies for the KOH/iPrOH and KOH/H_2O/iPrOH systems in irradiation setup 2 are summarized in Table S23:

Table S23: Calculated minimum quantum efficiency for the KOH/iPrOH and KOH/H_2O/iPrOH system in Irradiation Setup 2.

System	KOH/iPrOH	KOH/H_2O/iPrOH
$\dot{N}_{photons}$	1.8 mmol/h	1.8 mmol/h
$\dot{N}_{SF6}$	4.8 mmol/h	7.5 mmol/h
$\boldsymbol{\Phi_{min}}$	**2.7**	**4.2**

Interpretation of Quantum Efficiency

A quantum efficiency greater than 1 indicates that SF_6 degradation is not solely driven by the autocatalysis cycle (where 1 photon degrades 1 SF_6 molecule). Instead, it suggests that the ketyl radical anion is regenerated through a redox catalysis cycle, allowing 1 photon to degrade more than 2 SF_6 molecules.

Mechanisms and Theoretical Quantum Efficiency:

1. Autocatalysis (equation 25):

- The ketyl radical anion reduces SF_6 to $SF_6^{\cdot-}$ radical anion, which is further reduced by another ketyl radical anion to form SF_5^- and F^-.
- 1 photon degrades 1 SF_6 molecule
- Theoretical quantum efficiency: $\Phi_t = 1$

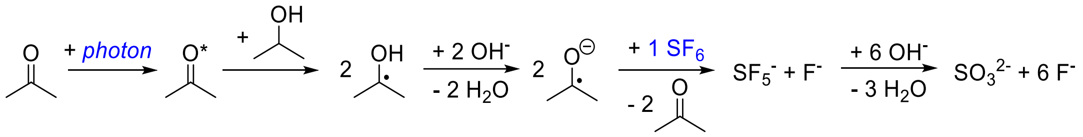

(25.)

2. Autocatalysis + Redoxcatalysis (equation 26):

- The ketyl radical anion reduces SF_6 to $SF_6^{\cdot-}$ radical anion, which undergoes hydrogen atom transfer to form SF_5^- and HF.
- 1 photon degrades 2 SF_6 molecule
- Theoretical quantum efficiency: $\Phi_t = 2$

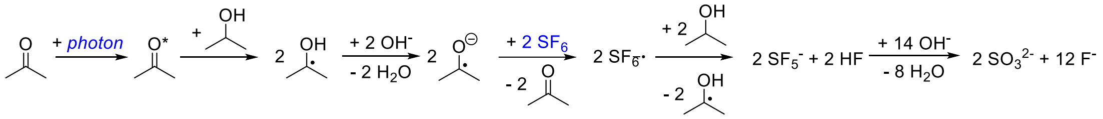

(26.)

3. Autocatalysis + Redoxcatalysis Chain Reaction (equation 27):

- The ketyl radical anion reduces SF_6 to $SF_6^{\cdot-}$ radical anion, which undergoes hydrogen atom transfer to form SF_5^- and HF. The ketyl radical formed in this reaction regenerates the ketyl radical anion, enabling a chain reaction.
- 1 photon degrades >2 SF_6 molecules
- Theoretical quantum efficiency: $\Phi_t > 2$

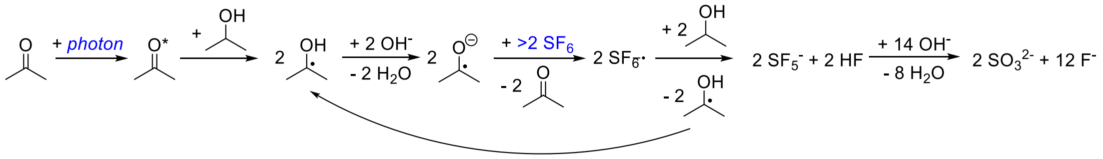

(27.)

This analysis highlights the role of redoxcatalysis in enhancing the reaction beyond the limitations of simple autocatalysis.

8.10 Pulsed Irradiation to Assess the Contribution of the Dark Reaction

To determine whether, and to what extent, the dark reaction (redox catalysis) contributes to the SF_6 degradation rate, two experiments were conducted following **GP2** (KOH: 1.0 g, 16.8 mmol; isopropanol (HPLC grade): 15 ml; SF_6 pressure: 2 bar; see chapter 4.2). In both experiments, sub-stoichiometric amounts of acetone (0.02 mL, 0.3 mmol) were added to the reaction mixture, which was then irradiated. The analysis for both experiments was performed according to **GP2**. The corresponding SF_6 uptake time plots are shown in Figure S59-Figure S61.

Experiment 1: The reaction mixture was irradiated continuously for 20 minutes, after which the irradiation was stopped. The SF_6 uptake was monitored using the PCU (Figure S59).

Experiment 2: The reaction mixture was irradiated continuously for 10 minutes, followed by 30 minutes without irradiation. Subsequently, the reaction mixture was irradiated for 2 minutes, followed by another 30 minutes without irradiation. This cycle of 2 minutes of irradiation and 30 minutes of no irradiation was repeated five times, resulting in a total irradiation time of 20 minutes. The SF_6 uptake was monitored using the PCU (Figure S60).

It is important to note that the addition of SF_6 by the PCU does not directly reflect the SF_6 degradation at the exact moment in time. This is because the consumption of SF_6 in the solution does not cause an immediate pressure drop in the gas phase but rather a gradual decrease. As a result, there is a time delay between the actual consumption of SF_6 and the addition of SF_6 by the PCU.

Results and Observations:

Experiment 1: Upon starting the irradiation, the SF_6 uptake increased until a constant reaction rate was reached. When the irradiation was stopped, the SF_6 uptake decreased, and the observed uptake was attributed to the saturation of the reaction mixture with SF_6. The overall yield of the reaction was 85%, based on reaction equation 11.

Experiment 2: During the first 10 minutes of continuous irradiation, the SF_6 uptake increased until the maximum degradation rate was reached. When the irradiation was turned off, the SF_6 uptake decreased, and the solution became saturated with SF_6. After 30 minutes without irradiation, the reaction mixture was irradiated for 2 minutes, resulting in a time-delayed increase in SF_6 uptake, followed again by saturation of the reaction mixture with SF_6. This cycle of turning the light source on and off was repeated five times, with the SF_6 uptake following a similar pattern in each cycle. The overall yield of the reaction was 94%, based on reaction equation 11.

The higher yield observed in Experiment 2 (94%) compared to Experiment 1 (85%) suggests that the light-independent dark reaction (redox catalysis) contributes to the SF_6 degradation process. However, the extent of this contribution is limited, as the redox catalysis does not exhibit long-term stability in the reaction mixture and ceases shortly after the irradiation is stopped.

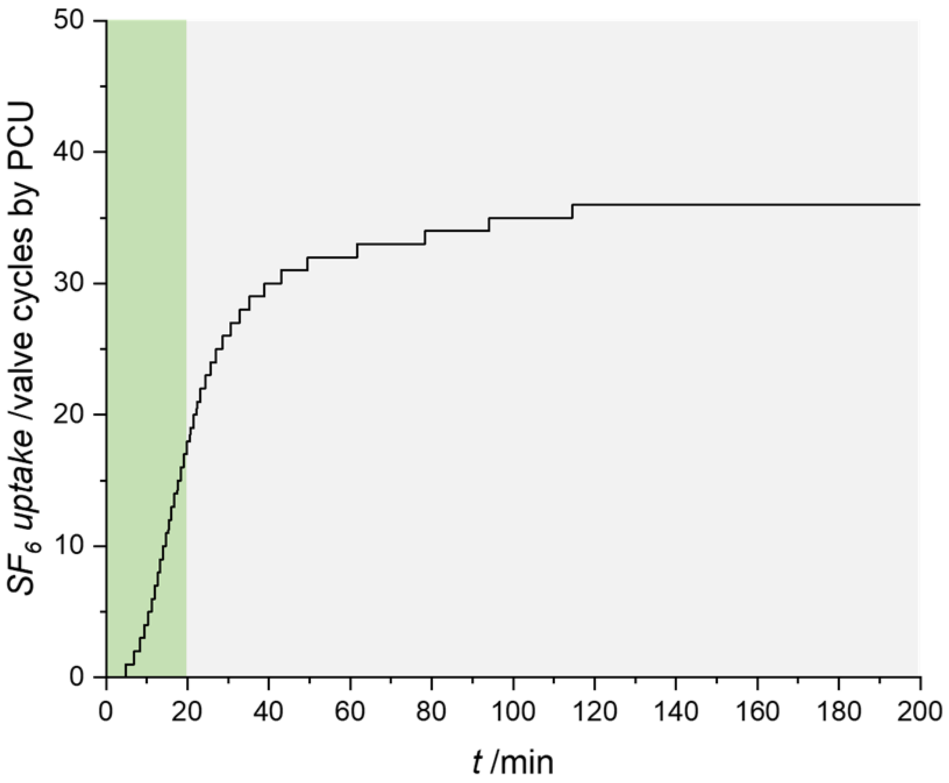

Figure S59: SF_6 uptake time plot for the KOH/iPrOH system (**GP2**) obtained by the PCU when only for the first 20 min the solution is irradiated. (green background = irradiation; grey background = no irradiation)

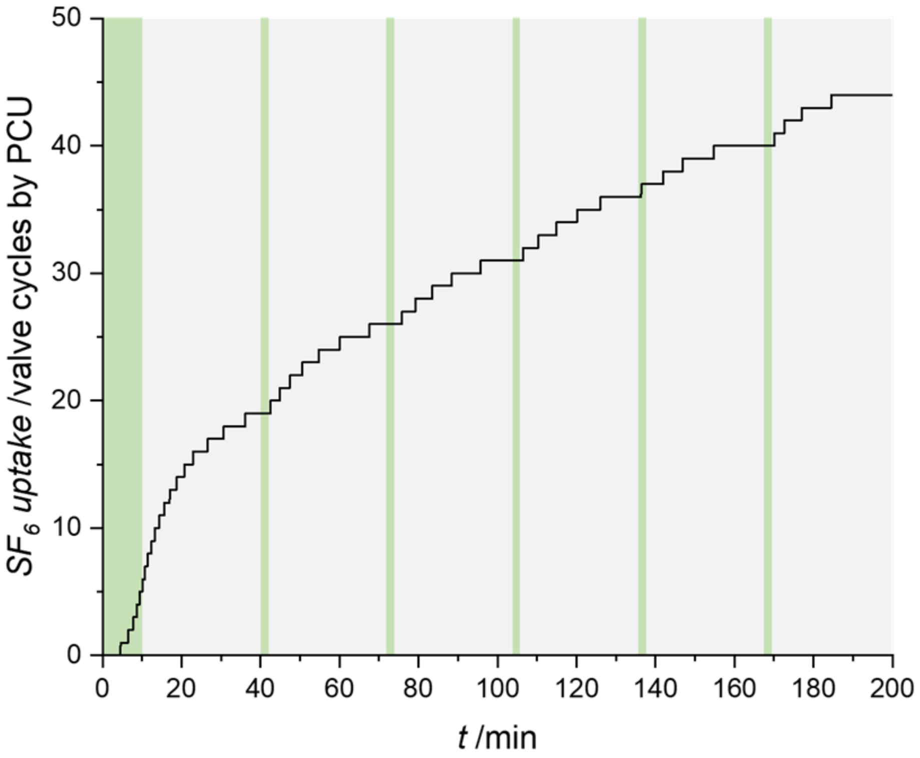

Figure S60: SF_6 uptake time plot for the KOH/iPrOH system (**GP2**) obtained by the PCU showing the effect of pulsating the LED on the reaction rate. (green background = irradiation; grey background = no irradiation)

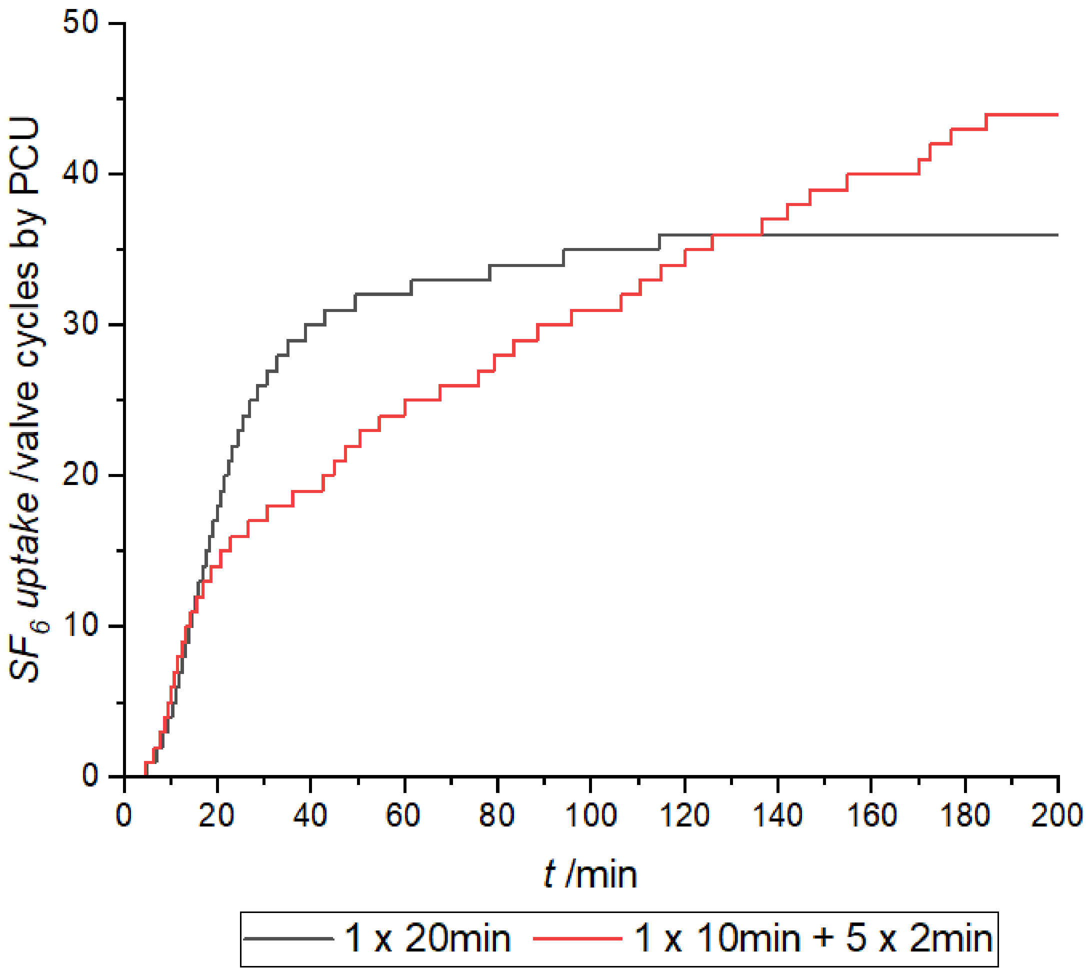

Figure S61: SF_6 uptake time plot for the KOH/iPrOH system (**GP2**) obtained by the PCU showing the difference between irradiating the solution for 20 min (black) and pulsating the LED with a total irradiation time of 20 min (red).

8.11 Influence of the isotope effect using the biphasic KOH/D_2O/iPrOH System

To determine whether there is a kinetic isotope effect (KIE) and to gain an insight into the reaction mechanism, D_2O was used instead of H_2O in the typical irradiation experiment of the biphasic system following **GP2** (KOH: 1.0 g, 16.8 mmol; D_2O: 2 mL; isopropanol (HPLC grade): 15 ml; SF_6 pressure: 2 bar; irradiation time: 2 h; see chapter 4.2). The biphasic system consisting of a layer of isopropanol on top of a concentrated solution of KOH in D_2O was vigorously stirred and the generated emulsion was irradiated for 2 h. After that, an aliquot of 0.7 ml was taken from the organic phase and analysed by NMR spectroscopy. The reaction yield was determined as described in **GP2** to be quantitative (>99%).

Monitoring the SF_6 uptake revealed no KIE, as both systems exhibited identical reaction rates (Figure S62). This suggests that the reaction rate is not limited by hydrogen atom transfer but is instead limited by photon availability or mass transfer.

NMR analysis of the organic phase after the irradiation experiment showed the formation of HDO, isopropanol-d_1 (with deuterium incorporated into the hydroxy group, C_α-position, or C_β-position), and acetone-d_1 (Figure S63).[11–13] The deuteration of the hydroxy group of isopropanol and the formation of acetone-d_1 are expected under the basic conditions of the system. Notably, acetone is known to slowly incorporate deuterium at the C_β-position in the presence of D_2O, even under weakly basic conditions.[14]

Consistent with the autocatalysis cycle, photoexcitation of acetone-d_1 generates two ketyl radicals, one of which carries deuterium at the C_β-position. This radical undergoes hydrogen atom transfer with a second isopropanol molecule, leading to the formation of isopropanol-d_1. Furthermore, the observed scrambling of deuterium into the α-position of isopropanol indicates that radical formation is reversible and confirms the in-situ generation of ketyl radicals.

To verify that the incorporation of deuterium occurred during the photocatalytic reaction and was not due to natural abundance, the organic phase of the KOH/H_2O/iPrOH system was also analyzed using ^{2}D and ^{13}C{^{1}H} NMR spectroscopy (Figure S64 and Figure S66).

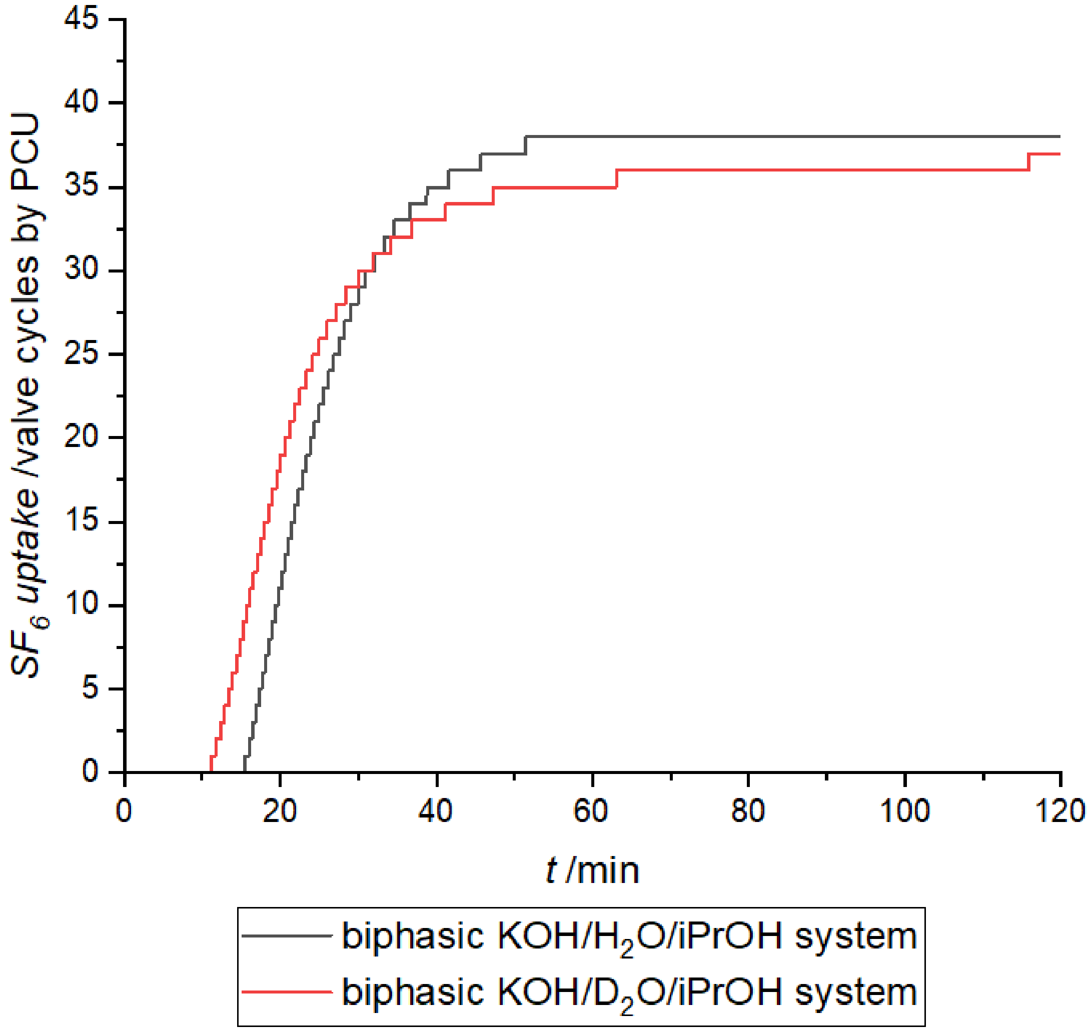

Figure S62: Uptake time plot obtained by the PCU (vide supra) of the biphasic KOH/H_2O/iPrOH system (black) and the biphasic KOH/D_2O/iPrOH system (red) following **GP2**. Note: The slight time differences in the first SF_6 repump cycle are due to slight variation in removing efficiency of the air atmosphere by 3 freeze-pump-thaw cycles before the experiment.

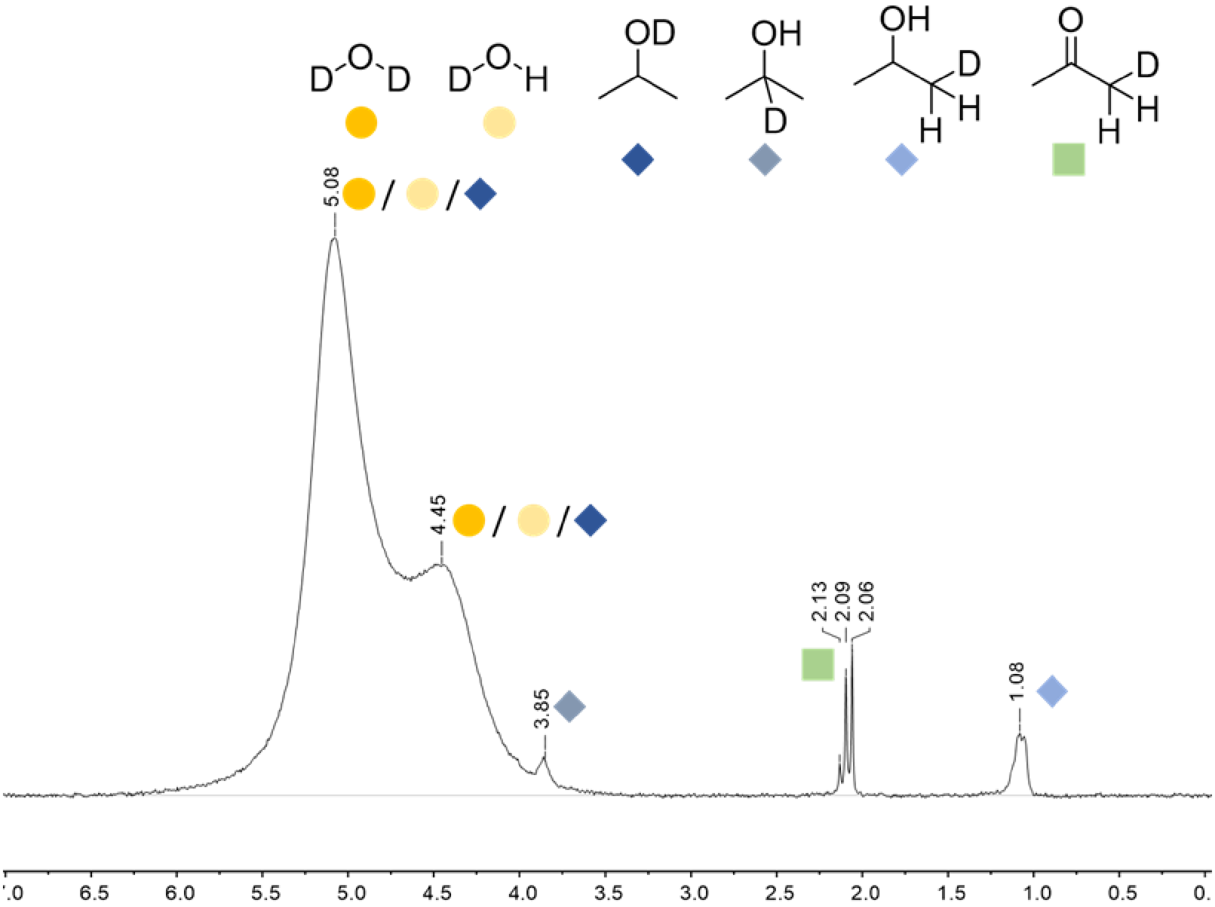

Figure S63: ^{2}D NMR spectrum of the organic phase after irradiation of the KOH/D_2O/iPrOH system at 280 nm for 2 hours. Note: The deuterium signals were references to the ^{1}H NMR chemical shift of the methyl group of isopropanol (δ = 1.08 ppm) since the primary isotope shift between ^{1}H and ^{2}D NMR is usually less than 0.1 ppm.[15] **^{2}D NMR** (61 MHz, reaction mixture): δ (ppm) = 5.08, 4.45 (br, D_2O, HDO, iPrOD); 3.85 (br, HOC*D*Me_2); 2.09 (t, OCMe{CH_2*D*}, $^2J_{HD}$ = 2.2 Hz and aceton-d_2); 1.08 (br, HOCHMe{CH_2*D*}).

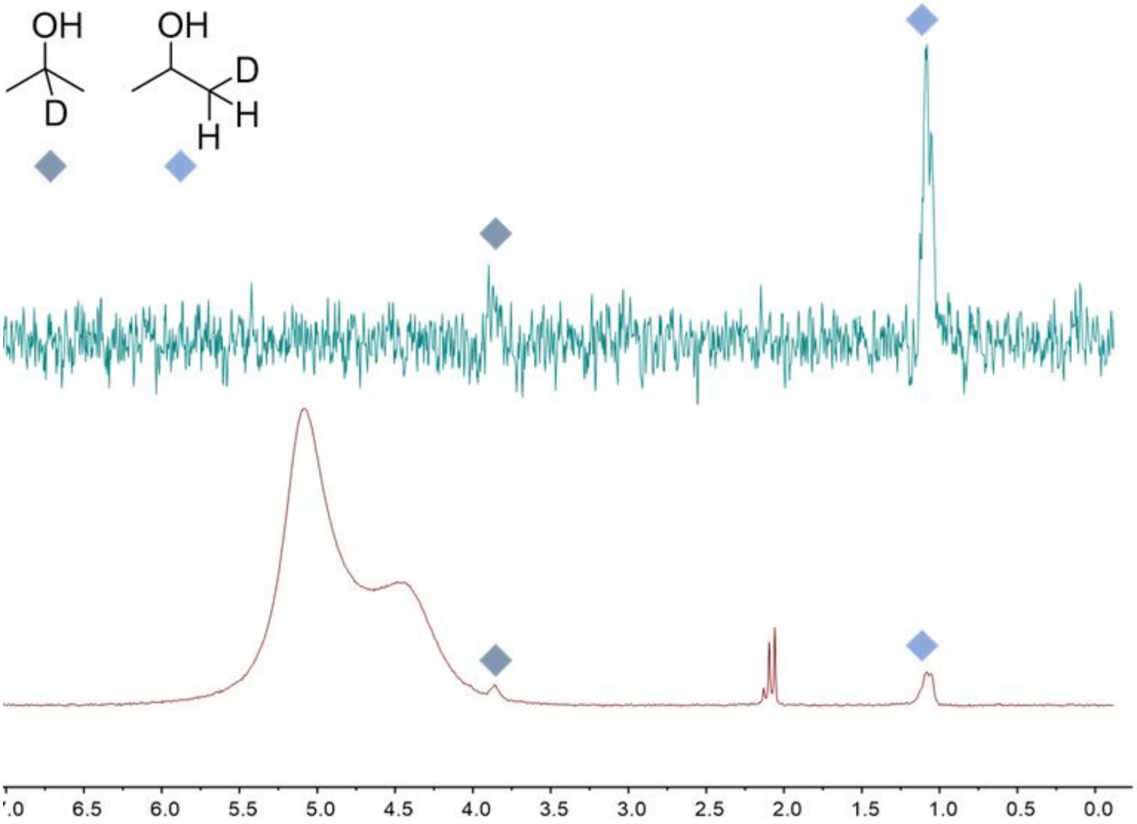

Figure S64: Stacked ^{2}D NMR spectra of the organic phase after following **GP2** (biphasic system) with H_2O (blue) and D_2O (red).

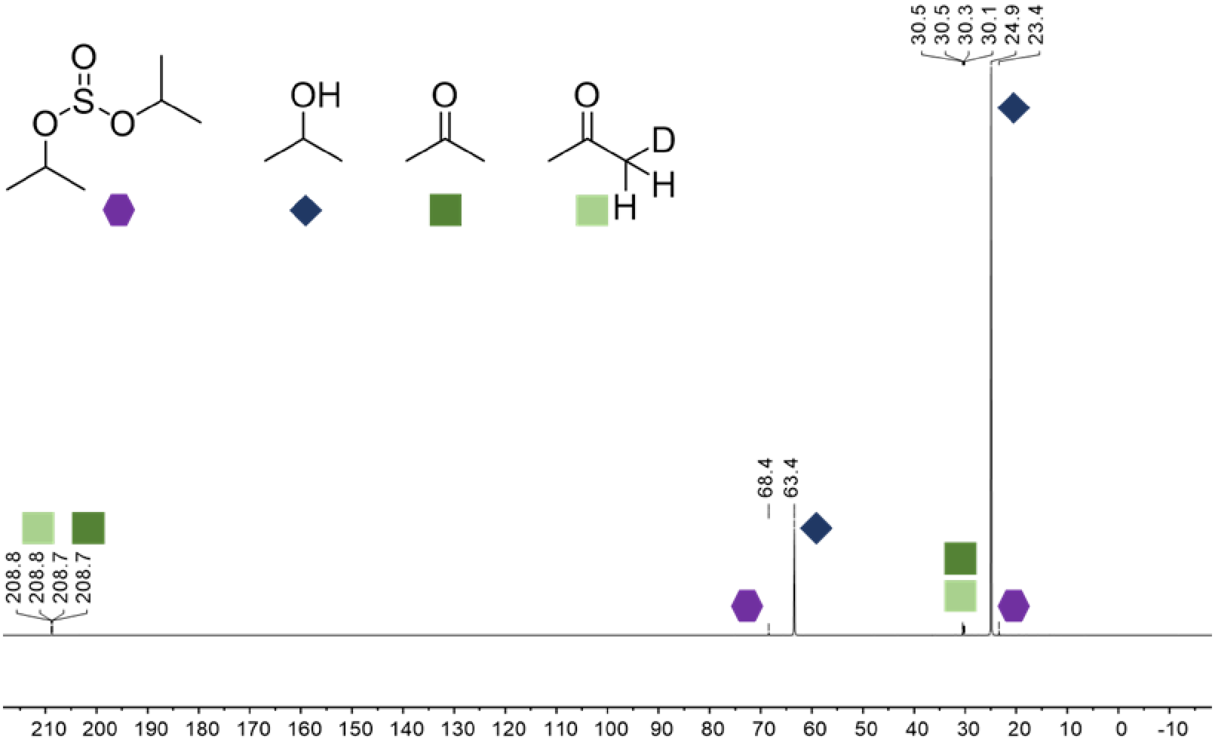

Figure S65: ^{13}C NMR spectrum of the organic phase after 2 h of irradiation at 280 nm of the KOH/D_2O/iPrOH system. **^{13}C{^{1}H} NMR** (101 MHz, reaction mixture): δ = 208.8 (t, O*C*{CH_3}{CH_2D}), 208.7 (s, O*C*Me_2), 68.4 (s, OS{O*C*HMe_2}$_2$), 63.4 (s, HO*C*HMe_2), 30.5 (s, OC{*C*H_3}$_2$), 30.3 (t, OC{CH_3}{*C*H_2D}), 24.9 (s, HOCH{*C*H_3}$_2$), 23.4 (s, OS{OCH[*C*H_3]$_2$}$_2$) ppm

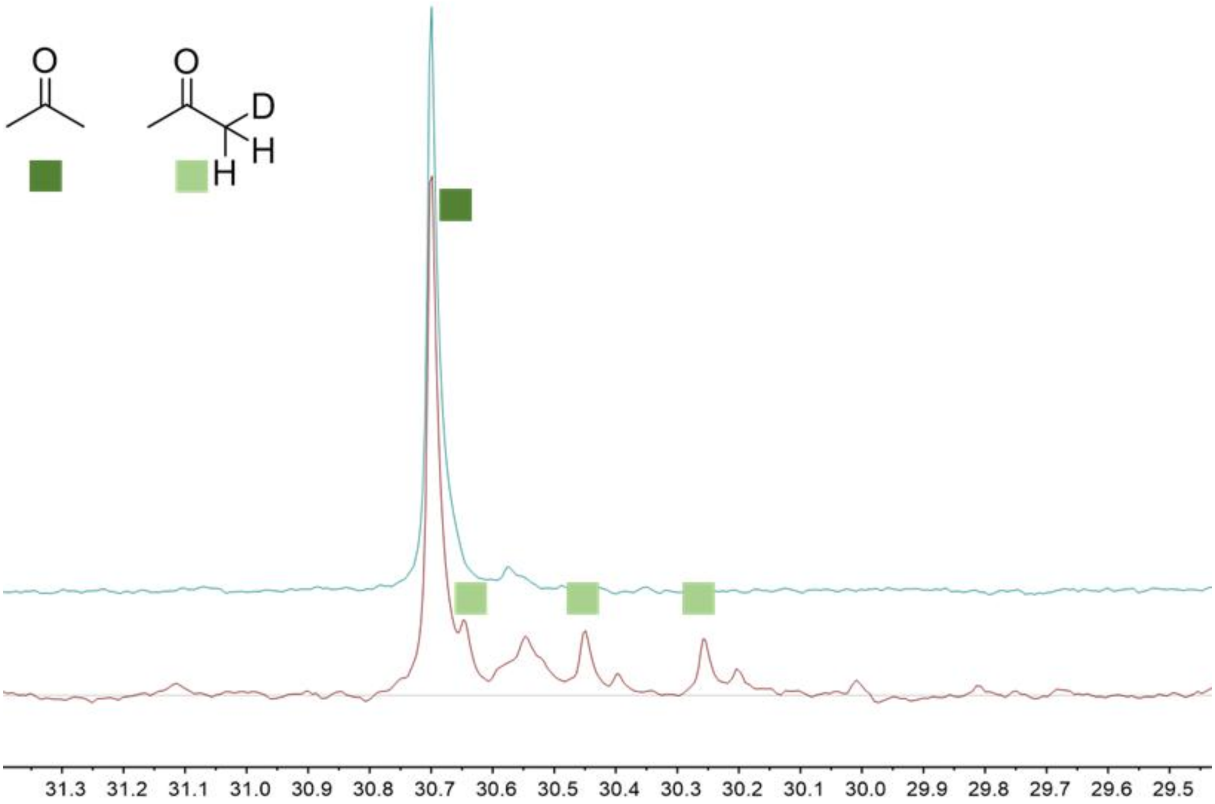

Figure S66: Zoom (31.4-29.5 ppm) of the stacked ^{13}C NMR spectra of the organic phase after following **GP2** (biphasic system) using H_2O (blue) and D_2O (red).

8.12 SF_6 degradation rate in quartz glass and borosilicate glass vessels

To determine whether quartz glass equipment is essential for the SF_6 degradation reaction, the reaction was conducted using a borosilicate glass vessel instead of quartz glass, following **GP2** (KOH: 1.0 g, 16.8 mmol; isopropanol (HPLC grade): 15 ml; SF_6 pressure: 2 bar; irradiation time: 2 h; see chapter 4.2). The use of borosilicate glass resulted in a significantly lower reaction rate compared to quartz glass (Table S24, Figure S67). This difference is attributed to the much lower transmittance of borosilicate glass at 280 nm.[16]

Table S24: Yields based on fluoride formation (determined by ^{19}F qNMR spectroscopy) assuming KOH conversion according to equation 11: 7.42 KOH + SF_6 + 1.58 iPrOH → 6 KF + 0.71 K_2SO_3 + 0.29 $(iPrO)_2SO$ + 1 acetone + 5 H_2O.

Glass	Maximum degradation rate (g/h)	Yield (%)
quartz	0.7	>99
borosilicate	0.2	98

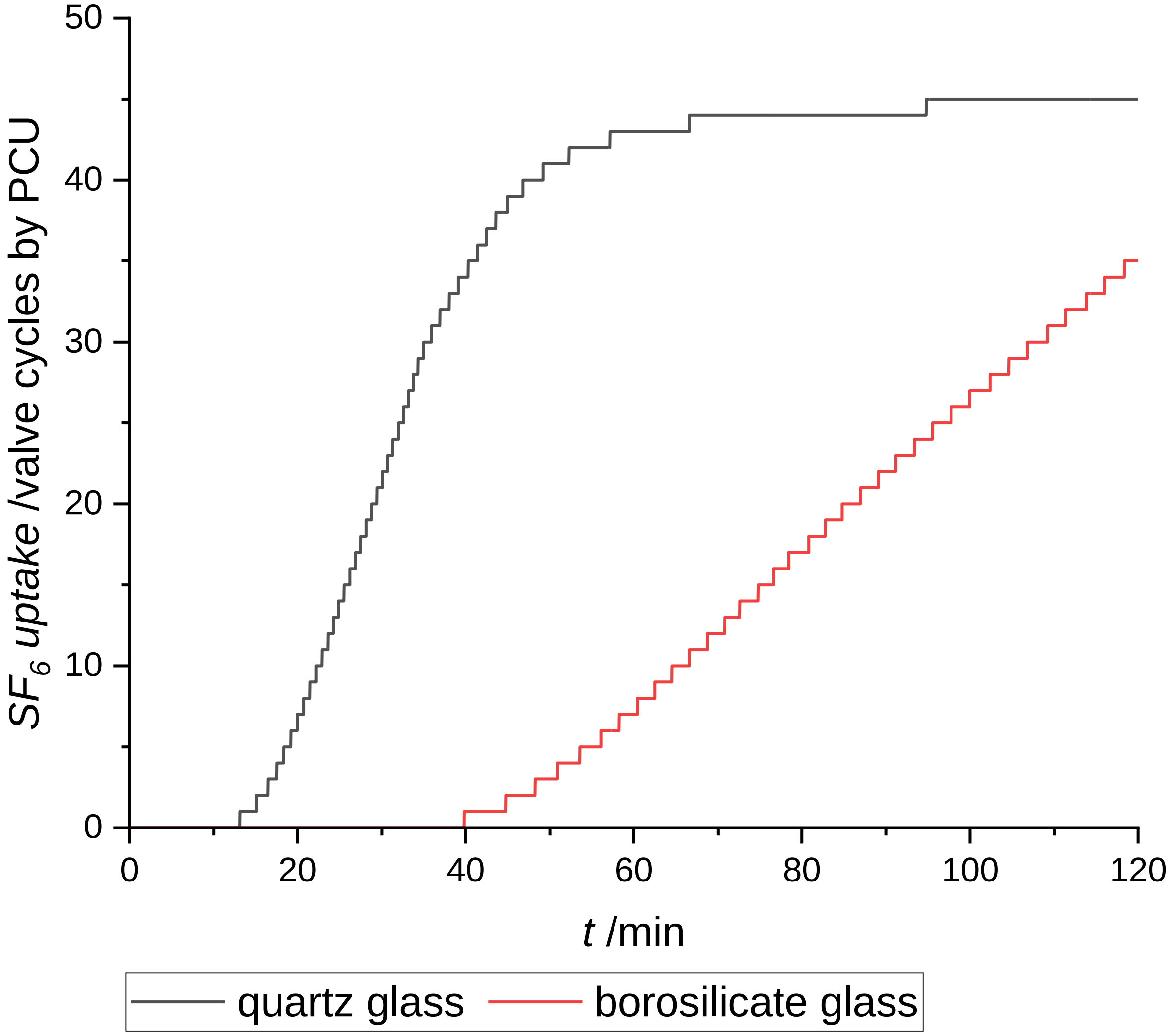

Figure S67: SF_6 uptake time plot for the KOH/iPrOH system (**GP2**) obtained by the PCU showing the influence of the glass on the reaction rate.

9 Degradation rates and energy efficiencies of the different setups

Table S25: Degradation rates and energy efficiencies of different setups and operational methods. The maximum degradation rate was determined using linear regression analysis of the SF_6 uptake time plot at its steepest point.

Experiment	time until full conversion (h)	Degraded SF_6 (g)	Maximum degradation rate (g/h)	Maximum energy efficiency (g/kWh)
Setup 2 (KOH/iPrOH system)	0.5	0.34	0.7	41
Setup 2 (KOH/H_2O/iPrOH system)	0.83	0.34	1.1	65
Setup 2 (KOH/H_2O/iPrOH system; 0.02 ml acetone)	0.34	0.34	1.1	65
Setup 3 (KOH/iPrOH system)	21	1.5	0.1	6
Setup 4 (KOH/iPrOH system)	24	19	5*	42
Setup 4 (KOH/H_2O/iPrOH system)	5	19	12	100
Setup 4 (KOH/H_2O/iPrOH system; 1.0 ml acetone, argon)	2	19	12	100

*at around 2h of irradiation

10 Overview of selected solution-based SF_6 degradation methods reported in the literature

Table S26: Overview of selected solution-based methods for the fragmentation of SF_6 and their conditions. The degradation rates were determined based on the available data in the respective publications. To note is, that most of these reactions were not optimized with respect to their SF_6 degradation rate.

Publication	Procedure	Catalyst	Stoichiometric starting material	Solvent	Products	Degradation rate (g/h)
Röschenthaler 2002[17]	Room temperature		Tetrakis(dimethylamino)ethylene; 1-Phenlyethanol	Dimethylformamid	1-Fluor-1-phenylethan	0.03
Ernst 2007[18]	Room temperature		Transition metal complexes (V, Cr, Fe)	*n*-hexane, toluol, THF	Fluorinated transition metal complexes (V, Cr)	0.04
Braun 2015[19]	80 °C	$Rh[(H)(PEt_3)_3]$	PR_3; $HSiEt_3$	toluene	SPR_3; $FSiEt_3$; F_2PR_3, H_2	0.003
Jamison 2016[20]	Blue LED	$Ir(ppy)_2(dtbbpy)PF_6$	DIPEA; allylic alcohol	DCE	Allylic fluorides	0.09
Kraus 2017[21]	-60 °C	-	Li-Cs; Sr	Liquid NH_3	MF, M_2S	0.3
Braun 2017[22]	60 °C	-	$[Pt(PR_3)_2]$; PR_3	toluene	*trans*-$[Pt(F)(SF_3)(PR_3)_2]$; F_2PR_3	0.002
Rueping 2017[23]	Room temperature	-	2,2'bipyrdine derivatives	toluene	2,2'bipyrdine derivatives^{2+} $SF_5^-F^-$	1.2
Beier 2018[24]	60 °C	-	Alken, TEMPO, $Li^+Ph_2CO^-$	THF	R-SF_5, R-F, …	0.03
Dielmann 2018[25]	-78 °C; 21 °C; 150 °C	-	PR_3	THF, Et_2O	F_2PR_3; SPR_3 or $[(R)_3PF][SF_5]$	0.05
Braun 2018[26]	311 nm	-	NHC	toluene	Difluoro imidazolidine; imidazolidine sulfide	0.00001
Wagenknecht 2018[27]	365 nm and 525 nm	N-Phenylpheno-thiazine; $Cu(acac)_2$	α-methyl and phenyl styrene	acetonitrile	Pentafluoro-sulfanylated α-methyl and phenyl styrene	0.0004

Moon 2020[28]	Electro-chemical	$Ag(NO_3)_2$; $[Ni(CN)_4]^{2-}$		5 M H_2SO_4; 10 M KOH	SO_4^{2-}; HF; O_2	0.03
Magnier 2020[29]	Electro-chemical	Pt electrode	acetonitrile	acetonitrile	F^-, S^{2-}, NC-$(CH_2)_2$-CN	not determined
Nagorny 2020[30]	365 nm	DMBP	DIPEA; benzylated hexose	THF	Fluorinated benzylated hexose	0.003
Hoge 2020[31]	Room temperature	-	$[R_3PNH\textit{tBu}]$ $[^{MeOtBu2}PhO]$	diethyl ether	$[R_3PNH\textit{tBu}]_2SF_5^-F^-$	0.0003
Speed 2021[32]	Room temperature	-	$KPPh_2$	THF	Ph_2PPPh_2, S_8, K_2S, KF	not determined
Crimmin 2021[33]	Room temperature	-	$[\{(ArNCMe)_2CH\}Al]$	benzene	$[\{(ArNCMe)_2CH\}AlF_2]$ $[\{(ArNCMe)_2CH\}Al(\mu\text{-}S)]_2$	0.002
Dielmann 2022[34]	365 nm	-	PPh_3	Polysiloxane	F_2PPh_3; $SPPh_3$	3.1
Tlili 2022[35]	455 nm	-	TDAE	pentane	$[TDAE][(SF_5^-)(F^-)]$	0.01
Dielmann 2024[36]	365 nm	-	$P(tmg)_3$	THF	$[(tmg)_3PF][SF_5]$	0.4
Cornella 2024[37]	60 °C	*N,C,N*-bismuthnidenes	PMe_3	acetonitrile	F_2PMe_3, $SPMe_3$	0.005
Lan-Gui Xie 2025[38]	Blue LED	$Ir(ppy)_2(dtbbpy)PF_6$	DIPEA; propargyl alcohol	DCE	Propargyl fluoride	0.02

11 Overview of selected gas phase photochemical SF_6 degradation methods reported in the literature

Table S27: Overview of photochemical SF_6 degradation methods for the fragmentation of SF_6 and their conditions. The degradation rates were determined based on the available data in the respective publications. To note is, that most of these reactions were not optimized with respect to their SF_6 degradation rate.

Publication	Light source	Stoichiometric starting material	condition	Products	Degradation rate (g/h)
Hou 2006[39]	Low-pressure Mercury lamp	acetone	in the gas phase	COS, HF, Ch_3CF_3, …	0.07
Hou 2007[40]	Low-pressure Mercury lamp	propene	In the gas phase	C_2F_6, C_3H_7F, SiF_4, …	0.001
Hou 2007[41]	Low-pressure Mercury lamp	styrene	In the gas phase	SiF_4, CH_3F, C_6H_5F, S, …	0.001
Hou 2009[42]	Low-pressure Mercury lamp	polyisoprene	In the gas phase	F and S introduced into the polyisoprene	0.01
Yamada 2011[43]	193 nm Laser	H_2O	Xenon matrix	SF_4, HF, HOF, …	Not enough data
Zai 2024[44]	365 nm LED	$Bi_2O_2CO_3$	Gas-solid reaction	Bi_2S_3, BiOF, …	0.0001

12 Computational Details

12.1 Methods

To estimate the energy required to detach an electron from potential donors and to evaluate the influence of solvation on this process, the vertical detachment energies (VDE) of the donors OH^-, $iPrO^-$ and pure $iPrOH$ were examined. Therefore, theoretical calculations at four different density function theory (DFT) levels (*i.e.* B3LYP,[45] ωB97XD,[46] CAM-B3LYP,[47] HSE06[48]) in conjunction with 6-311++G(d,p)[49,50] as basis set, were performed for each molecule. All of the latter energy minimizations were carried out not only *in vacuo* but also in implicit $iPrOH$ solvent, modeled by the polarizable continuum model (PCM).[51,52] For the estimation of VDEs, time-dependent DFT (TDDFT)[53] calculations for the optimized geometries of the molecules considering all four DFT methods were carried out similar to previous works.[54,55] Each of the described TDDFT calculations was performed *in vacuo* and in implicit solvent with two different basis sets, *i.e.* 6-311++G(d,p) and aug-cc-pVTZ.[56,57] In the latter case minimum geometries at DFT/6-311++G(d,p) were taken.

For a more accurate determination of the energy required to transfer an electron from OH^- to the SF_6 molecule, energy minimization and subsequent TDDFT calculations were performed on a combined $SF_6\cdots OH^-$ system. These calculations were carried out at the ωB97XD/6-311++G(d,p)/PCM level of theory, using tight convergence criteria for energy and gradients.

Additionally, the potential dissociation pathways of $SF6^{\bullet-}$ and subsequent hydrogen atom transfer reactions were investigated using ωB97XD/6-311++G(d,p)/PCM.

All obtained structures were confirmed to be actual minima on the potential energy surface by associated frequency calculations required also for the thermochemical analysis. The software package Gaussian16[58] was used to carry out all of the mentioned calculation setups.

12.2 Results

12.2.1 Photo-induced formation of $SF_6^{\bullet-}$

Vertical excitation energetics of potential electron donors. Table S28 lists the wavelengths of the first absorption band, which serves as a estimation of VDE[54,55] calculated at the different levels of DFT of the excited state calculations.

Firstly, a small difference in the calculated wavelengths between vacuum and implicit solvent conditions is observed for the uncharged iPrOH molecule. In contrast, significant changes are seen for the charged ions OH^- and $iPrO^-$. This difference can be attributed to the stabilization of the exposed negative charge of the anions by solvent effects, which consistently results in higher excitation energies (i.e., shorter wavelengths).

When comparing the applied basis sets, the aug-cc-pVTZ basis set produces wavelengths closer to experimentally expected values than 6-311++G(d,p). This improvement arises from the enhanced treatment of the charged character of OH^- and $iPrO^-$ due to the additional diffuse basis functions in aug-cc-pVTZ. This is further supported by the much smaller wavelength difference observed for the uncharged iPrOH species when comparing the two basis sets.

Furthermore, the calculated wavelengths in Table S28 indicate that the light source (280 nm) provides sufficient energy to detach an electron from both OH^- or $iPrO^-$ (approximately 280–300 nm), whereas it does not provide enough energy to trigger vertical excitation of iPrOH, either in vacuo or in implicit solvent. These findings highlight the necessity of the highly basic conditions (KOH) to enable the initial photoreaction triggered by light at 280 nm leading to the formation of radicals which subsequently enable the autocatalysis cycle.

Table S28: Wavelengths of the first vertical electronic excitation in nm for OH^-, $iPrO^-$ and $iPrOH$ obtained via TDDFT calculations using four different functionals and two different one-electron bases. The comparison between the values obtained in vacuo and in implicit solvation provide a direct estimate of charge-transfer-to-solvent effects (all calculations were performed at minimum structure obtained at DFT/6-311++G(d,p) level.

	**B3LYP**		**ωB97XD**		**CAM-B3LYP**		**HSE06**	
	vac	PCM	vac	PCM	vac	PCM	vac	PCM
**6-311++G(d,p)**								
OH^-	528.9	285.5	466.9	268.5	469.8	270.5	482.3	271.6
$iPrO^-$	582.2	321.7	422.7	278.4	447.5	284.4	546.7	305.0
$iPrOH$	198.6	190.4	182.9	175.9	187.7	180.1	190.7	183.1
**aug-cc-pVTZ**								
OH^-	618.4	296.0	535.4	278.5	515.1	276.6	563.8	282.5
$iPrO^-$	649.3	328.3	464.2	289.0	480.4	291.8	608.2	311.9
$iPrOH$	199.1	191.9	184.2	178.1	187.8	181.1	191.2	184.5

Charge-transfer energetics for the SF_6···OH^- system. While the above mentioned TDDFT calculations using range-separated hybrid functionals in implicit solvent provide valuable insights into the photo-induced charge-transfer-to-solvent energetics, the excitation energy associated with electron transfer from the most abundant donor, OH^-, to the electron acceptor, SF_6, was examined by considering the combined complex between SF_6 and OH^-. Energy minimization was performed for both hydrogen-bonded and halogen-bonded encounter complexes, and stable minimum structures were identified (see Figure S68), albeit very small interaction energies ΔE of -1.7 and -0.2 kJ mol^{-1} were identified with the corresponding ΔG values being 22.6 and 28.8 kJ mol^{-1}, respectively. Despite the weak interactions, the associated minima were confirmed by the presence of only real vibrational frequencies, indicating true minima on the potential energy surface.

Subsequent TDDFT calculations revealed the first excitation energies for the hydrogen- and oxygen-bonded encounter complexes at 317.6 nm and 311.6 nm, respectively. However, the associated oscillator strengths were very small, at only 0.005 and 0.003, respectively, suggesting these transitions are unlikely to play a significant role. In contrast, the next observed excitations at 258.6 nm (H-bonded complex) and 268.0 nm (O-bonded complex) not only match the wavelength used in the experimental setup but also exhibit significantly higher oscillator strengths of 0.0854 and 0.0732, respectively. Analysis of the molecular orbitals involved in these excitations revealed that, in both cases, the transitions correspond to an excitation from the two highest occupied molecular orbitals (HOMO-1 and HOMO), localized on the oxygen atom of OH^-, to low-lying virtual orbitals (LUMO, LUMO+1, etc.) associated with the SF_6 molecule. These results provide strong evidence for a photo-induced intermolecular charge transfer process between OH^- and SF_6.

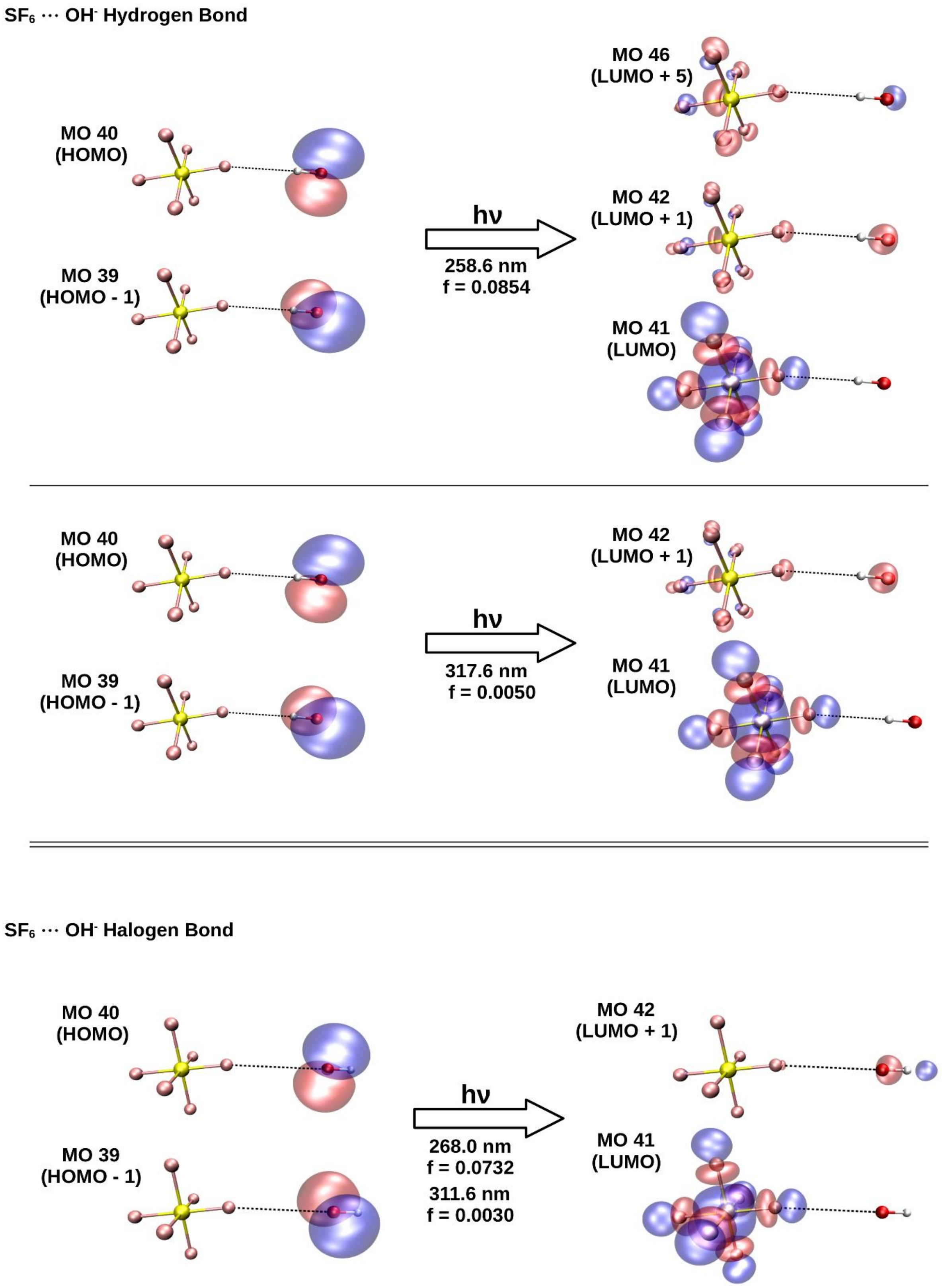

Figure S68: Visualization of the molecular orbitals (isovalue ±0.04) at the ωB97XD/6-311++G(d,p) level of theory, illustrating the intermolecular charge transfer in $SF_6\cdots OH^-$. The top and bottom panels show the hydrogen- and oxygen-bonded encounter complex, respectively.

In summary, the DFT results support the experimental findings that the initial activation of SF_6 requires strongly basic conditions and proceeds via charge-transfer excitation from OH^- (or, less

likely, iPrO$^-$) to SF_6. Additionally, it is worth noting that studies over the past decade have demonstrated the superior performance of range-separated hybrid functionals in TDDFT calculations,[59] as can be again underlined by the presented data, where B3LYP represents the only non-range-separated hybrid functional and features also the largest deviation not only compared to the expected wavelengths, but also to the resulting wavelengths of all other applied functionals.

12.2.2 Dissociation pathways of $SF_6^{\bullet-}$

The possible dissociation pathways of $SF6^{\bullet-}$ were investigated using ωB97XD/6-311++G(d,p)/PCM. The calculated energy differences ΔE and free energy differences ΔG for these pathways are listed in Table S29. While both dissociation reactions are clearly endotherm and endergonic, the formation of the $F^\bullet$ radical is slightly preferred. This finding is in line with earlier estimations of Chen *et al.*[60], reporting a dissociation energy of 1.35 eV (130 kJ mol^{-1}) based on atmospheric pressure ionization/mass spectroscopy (API/MS) measurements and a corresponding Morse potential energy curve analysis.

Table S29: Energy ΔE and free energy ΔG in kJ mol^{-1} for the two considered dissociation reactions of $SF_6^{\bullet-}$ obtained at ωB97XD/6-311++G(d,p)/PCM level or theory.

Reaction	ΔE	ΔG
$SF6^{\bullet-} \rightarrow SF_5^- + F^\bullet$	134.0	103.1
$SF6^{\bullet-} \rightarrow SF_5^\bullet + F^-$	140.4	118.2

12.2.3 Hydrogen atom abstraction from iPrOH by the radicals $F^\bullet$ and $SF_5^\bullet$

As discussed above, the dissociation of $SF6^{\bullet-}$ can produce either $F^\bullet$ or $SF_5^\bullet$ radicals, depending on the dissociation pathway. DFT calculations on the subsequent hydrogen atom abstraction from iPrOH reveal a significant difference in the energetics of these reactions (Table S30). Hydrogen atom abstraction by the fluorine radical ($F^\bullet$) is highly exothermic and exergonic, making it energetically favorable. In contrast, hydrogen atom abstraction by the $SF_5^\bullet$ radical is energetically unfavorable. This difference suggests that the radical chain reaction is sustained only when the dissociation of $SF_6^{\bullet-}$ produces $F^\bullet$ radicals. Conversely, dissociation into $SF_5^\bullet$ radicals effectively terminates the dark reaction, preventing the continuation of the redoxcatalysis cycle.

Table S30: Energy ΔE and free energy ΔG in kJ mol^{-1} for the two considered hydrogen atom transfer reactions of involving $F^\bullet$ and $SF_5^\bullet$ obtained at ωB97XD/6-311++G(d,p)/PCM level or theory.

Reaction	ΔE	ΔG
$F^\bullet + Me_2CHOH \rightarrow HF + [Me_2COH]^\bullet$	−164.1	−189.1
$F_5S^\bullet + Me_2CHOH \rightarrow HSF_5 + [Me_2COH]^\bullet$	+51.6	+51.0

12.2.4 Minimum Structures of $SF_6 \cdots OH^-$ Encounter Complexes

Hydrogen-bonded configuration:

E(ωB97XD/6-311++G(d,p)/PCM(iPrOH)) = -1073.05339662 Hartree

Lowest Frequency 17.5972 cm^{-1}

Charge: -1 Multiplicity: 1

The corresponding coordinate file is available as source data file SF6HO.xyz

Oxygen-bonded configuration:

E(ωB97XD/6-311++G(d,p)/PCM(iPrOH)) = -1073.05282475 Hartree

Lowest Frequency: 31.3489 cm^{-1}

Charge: -1   Multiplicity: 1

The corresponding coordinate file is available as source data file SF6OH.xyz

13 Supplementary References

1. VCC Optoelectronics. High Power Light VAOL-SX1XAX-SA. Available at https://www.mouser.com/catalog/specsheets/vcc_vaol-sx1xax-sa.pdf?_gl=1*1ij5snm*_gcl_au*MTM1ODQyNTMwNS4xNzYzMTIyMTQ1*_ga*dW5kZWZpbmVk*_ga_15W4STQT4T*czE3NjMxMjIxNDgkbzEkZzAkdDE3NjMxMjIxNDkkajU5JGwwJGgw (2025).
2. SEOULVIOSYS. UV CA3535 series (CUD1GF1A). Available at https://seoulviosys.com/en/product/detail/8 (2025).
3. HepatoChem. SKU: HCK1012-01-029. Available at https://hepatochem.com/product/hck1012-01-029/ (2023).
4. HepatoChem. SKU: HCK1012-01-010. Available at https://hepatochem.com/product/hck1012-01-010/ (2025).
5. LUMINUS. XBT-3535-UV Surface Mount UVC LED. Available at https://led-tech-shop.s3.eu-central-1.amazonaws.com/files/207/Luminus_XBT-3535-UV_Datasheet.pdf (2025).
6. PESCHL PHOTOREACTORS. Peschl Ultraviolet advanced uv-products. Available at http://uvcp-es.com/wp-content/uploads/2016/03/Ultravioleta-Fotoqu%C3%ADmica-L%C3%A1mparas-UV-para-el-sistema-MPDS-Basic.pdf (2025).
7. Topas (version 4.2). General Profile and Structure Analysis Software for Powder Diffraction Data & Bruker AXS: Karlsruhe (Germany) (2009).
8. Hamamatsu. Spot light sources lightningcure® series. Available at https://www.hamamatsu.com/content/dam/hamamatsu-photonics/sites/documents/99_SALES_LIBRARY/etd/LC8_TLSZ1008E.pdf (2025).
9. Atkins, P. W., Paula, J. de & Keeler, J. J. *Atkins' physical chemistry* (Oxford University Press, Oxford, New York, 2018).
10. Turro, N. J., Ramamurthy, V. & Scaiano, J. C. *Modern molecular photochemistry of organic molecules* (University Science Books, Sausalito, California, 2010).
11. Shahkhatuni, A. A., Shahkhatuni, A. G., Ananikov, V. P. & Harutyunyan, A. S. NMR-monitoring of H/D exchange reaction of ketones in solutions of imidazolium ionic liquids. *J. Mol. Liq.* **362,** 119746 (2022).
12. Wang, Y. *et al.* Customizing H2O-Poor Electric Double Layer and Boosting Texture Exposure of Zn (101) Plane towards Super-High Areal Capacity Zinc Metal Batteries. *Angew Chem. Int. Ed.* **64,** e202414757 (2025).
13. Pucheault, M., Darses, S. & Genet, J.-P. Direct access to ketones from aldehydes via rhodium-catalyzed cross-coupling reaction with potassium trifluoro(organo)borates. *J. Am. Chem. Soc.* **126,** 15356–15357 (2004).
14. Shahkhatuni, A. A., Shahkhatuni, A. G., Mamyan, S. S., Ananikov, V. P. & Harutyunyan, A. S. Proton-deuterium exchange of acetone catalyzed in imidazolium-based ionic liquid-D2O mixtures. *RSC advances* **10,** 32485–32489 (2020).
15. Davies, D. B., Christofides, J. C. & Hoffman, R. E. Isotope shifts in NMR spectroscopy - measurement and applications. In *Isotopes: essential chemistry and applications 2* (1988), pp. 147–172.
16. Gross, A., Stangl, F., Hoenes, K., Sift, M. & Hessling, M. Improved Drinking Water Disinfection with UVC-LEDs for Escherichia Coli and Bacillus Subtilis Utilizing Quartz Tubes as Light Guide. *Water* **7,** 4605–4621 (2015).

17. Kirsch, P., Roeschenthaler, G. V., Sevenrard, D. & Kolomeitsev, A. *Production of pentafluoro sulfuranide compounds, useful as organic fluorinating agents, involves reduction of sulfur hexafluoride with a divalent cation-forming reducing agent e.g. tetrakis dimethylamino-ethylene.* DE20021020901 (2002).

18. Harvey, B. G., Arif, A. M., Glöckner, A. & Ernst, R. D. SF 6 as a Selective and Reactive Fluorinating Agent for Low-Valent Transition Metal Complexes. *Organometallics* **26,** 2872–2879 (2007).

19. Zámostná, L. & Braun, T. Catalytic Degradation of Sulfur Hexafluoride by Rhodium Complexes. *Angew Chem. Int. Ed.* **54,** 10652–10656 (2015).

20. McTeague, T. A. & Jamison, T. F. Photoredox Activation of SF6 for Fluorination. *Angew Chem. Int. Ed.* **55,** 15072–15075 (2016).

21. Deubner, H. & Kraus, F. The Decomposition Products of Sulfur Hexafluoride (SF6) with Metals Dissolved in Liquid Ammonia. *Inorganics* **5,** 68 (2017).

22. Berg, C., Braun, T., Ahrens, M., Wittwer, P. & Herrmann, R. Activation of SF6 at Platinum Complexes: Formation of SF3 Derivatives and Their Application in Deoxyfluorination Reactions. *Angew Chem. Int. Ed.* **56,** 4300–4304 (2017).

23. Rueping, M., Nikolaienko, P., Lebedev, Y. & Adams, A. Metal-free reduction of the greenhouse gas sulfur hexafluoride, formation of SF 5 containing ion pairs and the application in fluorinations. *Green Chem.* **19,** 2571–2575 (2017).

24. Iakobson, G., Pošta, M. & Beier, P. Reductive activation of sulfur hexafluoride with TEMPOLi: Addition of the pentafluorosulfanyl group and TEMPO to terminal alkenes. *J. Fluor. Chem.* **213,** 51–55 (2018).

25. Buß, F., Mück-Lichtenfeld, C., Mehlmann, P. & Dielmann, F. Nucleophilic Activation of Sulfur Hexafluoride: Metal-Free, Selective Degradation by Phosphines. *Angew Chem. Int. Ed.* **57,** 4951–4955 (2018).

26. Tomar, P., Braun, T. & Kemnitz, E. Photochemical activation of SF6 by N-heterocyclic carbenes to provide a deoxyfluorinating reagent. *Chem. Commun.* **54,** 9753–9756 (2018).

27. Rombach, D. & Wagenknecht, H.-A. Photoredox Catalytic Activation of Sulfur Hexafluoride for Pentafluorosulfanylation of α-Methyl- and α-Phenyl Styrene. *ChemCatChem* **10,** 2955–2961 (2018).

28. Govindan, M., Adam Gopal, R. & Moon, I. S. Electrochemical sequential reduction and oxidation facilitates the continual ambient temperature degradation of SF6 to nontoxic gaseous compounds. *Chem. Eng. J.* **382,** 122881 (2020).

29. Bouvet, S. *et al.* Controlled decomposition of SF6 by electrochemical reduction. *Beilstein J. Org. Chem.* **16,** 2948–2953 (2020).

30. Kim, S., Khomutnyk, Y., Bannykh, A. & Nagorny, P. Synthesis of Glycosyl Fluorides by Photochemical Fluorination with Sulfur(VI) Hexafluoride. *Org. Lett.* **23,** 190–194 (2021).

31. Weitkamp, R. F., Neumann, B., Stammler, H.-G. & Hoge, B. Non-Coordinated Phenolate Anions and Their Application in SF6 Activation. *Chemistry* **27,** 6460–6464 (2021).

32. Huchenski, B. S. N. & Speed, A. W. H. Room-temperature reduction of sulfur hexafluoride with metal phosphides. *Chem. Commun.* **57,** 7128–7131 (2021).

33. Sheldon, D. J. & Crimmin, M. R. Complete deconstruction of SF6 by an aluminium(I) compound. *Chem. Commun.* **57,** 7096–7099 (2021).

34. Rotering, P., Mück-Lichtenfeld, C. & Dielmann, F. Solvent-free photochemical decomposition of sulfur hexafluoride by phosphines: formation of difluorophosphoranes as versatile fluorination reagents. *Green Chem.* **24,** 8054–8061 (2022).

35. Taponard, A. *et al.* Metal-Free SF6 Activation: A New SF5 -Based Reagent Enables Deoxyfluorination and Pentafluorosulfanylation Reactions. *Angew Chem. Int. Ed.* **61,** e202204623 (2022).

36. Röthel, M. B., Schöler, A., Buß, F., Löwe, P. & Dielmann, F. Phosphonium SF5- Salts Derived from Sulfur Hexafluoride as Deoxyfluorination Reagents. *Chem. Eur. J.,* e202402028 (2024).

37. Béland, V. A., Nöthling, N., Leutzsch, M. & Cornella, J. Activation and Catalytic Degradation of SF6 and PhSF5 at a Bismuth Center. *J. Am. Chem. Soc.* **146,** 25409–25415 (2024).

38. Zhao, Y. *et al.* Photoinduced SF6 degradation for deoxyfluorination of propargyl alcohols. *Org. Biomol. Chem.* **23,** 1094–1097 (2025).

39. Huang, L., Dong, W., Zhang, R. & Hou, H. Investigation of a new approach to decompose two potent greenhouse gases: photoreduction of SF(6) and SF(5)CF(3) in the presence of acetone. *Chemosphere* **66,** 833–840 (2007).

40. Huang, L. *et al.* A novel method to decompose two potent greenhouse gases: photoreduction of SF6 and SF5CF3 in the presence of propene. *J. Hazard. Mat.* **151,** 323–330 (2008).

41. Huang, L. *et al.* Photoreductive degradation of sulfur hexafluoride in the presence of styrene. *J. Environ. Sci.* **20,** 183–188 (2008).

42. Song, X. *et al.* Photodegradation of SF6 on polyisoprene surface: implication on elimination of toxic byproducts. *J. Hazard. Mat.* **168,** 493–500 (2009).

43. Yamada, Y., Tamura, H. & Takeda, D. Photochemical reaction of sulfur hexafluoride with water in low-temperature xenon matrices. *J. Chem. Phys.* **134,** 104302 (2011).

44. Zhou, W. *et al.* Efficient photocatalytic degradation of potent greenhouse gas SF6 at liquid-solid interface. *Appl. Catal., B* **363,** 124773 (2025).

45. Becke, A. D. Density-functional thermochemistry. III. The role of exact exchange. *J. Chem. Phys.* **98,** 5648–5652 (1993).

46. Chai, J.-D. & Head-Gordon, M. Long-range corrected hybrid density functionals with damped atom-atom dispersion corrections. *PCCP* **10,** 6615–6620 (2008).

47. Yanai, T., Tew, D. P. & Handy, N. C. A new hybrid exchange–correlation functional using the Coulomb-attenuating method (CAM-B3LYP). *Chem. Phys. Lett.* **393,** 51–57 (2004).

48. Paier, J. *et al.* Screened hybrid density functionals applied to solids. *J. Chem. Phys.* **125** (2006).

49. Clark, T., Chandrasekhar, J., Spitznagel, G. W. & Schleyer, P. V. R. Efficient diffuse function-augmented basis sets for anion calculations. III. The 3-21+G basis set for first-row elements, Li–F. *J. Comput. Chem.* **4,** 294–301 (1983).

50. Krishnan, R., Binkley, J. S., Seeger, R. & Pople, J. A. Self-consistent molecular orbital methods. XX. A basis set for correlated wave functions. *J. Chem. Phys.* **72,** 650–654 (1980).

51. Cossi, M., Barone, V., Cammi, R. & Tomasi, J. Ab initio study of solvated molecules: a new implementation of the polarizable continuum model. *Chem. Phys. Lett.* **255,** 327–335 (1996).

52. Miertuš, S., Scrocco, E. & Tomasi, J. Electrostatic interaction of a solute with a continuum. A direct utilizaion of AB initio molecular potentials for the prevision of solvent effects. *Chem. Phys.* **55,** 117–129 (1981).

53. Bauernschmitt, R. & Ahlrichs, R. Treatment of electronic excitations within the adiabatic approximation of time dependent density functional theory. *Chem. Phys. Lett.* **256,** 454–464 (1996).

54. Isborn, C. M., Mar, B. D., Curchod, B. F. E., Tavernelli, I. & Martínez, T. J. The charge transfer problem in density functional theory calculations of aqueously solvated molecules. *J. Chem. Phys.* **117,** 12189–12201 (2013).

55. Masamura, M. Structures, energetics, and spectra of OH−(H2O)n and SH−(H2O)n clusters, n=1–5: Ab initio study. *J. Chem. Phys.* **117,** 5257–5263 (2002).

56. Dunning, T. H. Gaussian basis sets for use in correlated molecular calculations. I. The atoms boron through neon and hydrogen. *J. Chem. Phys.* **90,** 1007–1023 (1989).

57. Kendall, R. A., Dunning, T. H. & Harrison, R. J. Electron affinities of the first-row atoms revisited. Systematic basis sets and wave functions. *J. Chem. Phys.* **96,** 6796–6806 (1992).

58. M. J. Frisch, G. W. Trucks, H. B. Schlegel, G. E. Scuseria, M. A. Robb, J. R. Cheeseman, G. Scalmani, V. Barone, G. A. Petersson, H. Nakatsuji, X. Li, M. Caricato, A. V. Marenich, J. Bloino, B. G. Janesko, R. Gomperts, B. Mennucci, H. P. Hratchian, J. V. Ortiz, A. F. Izmaylov, J. L. Sonnenberg, D. Williams-Young, F. Ding, F. Lipparini, F. Egidi, J. Goings, B. Peng, A. Petrone, T. Henderson, D. Ranasinghe, V. G. Zakrzewski, J. Gao, N. Rega, G. Zheng, W. Liang, M. Hada, M. Ehara, K. Toyota, R. Fukuda, J. Hasegawa, M. Ishida, T. Nakajima, Y. Honda, O. Kitao, H. Nakai, T. Vreven, K. Throssell, J. A. Montgomery, Jr., J. E. Peralta, F. Ogliaro, M. J. Bearpark, J. J. Heyd, E. N. Brothers, K. N. Kudin, V. N. Staroverov, T. A. Keith, R. Kobayashi, J. Normand, K. Raghavachari, A. P. Rendell, J. C. Burant, S. S. Iyengar, J. Tomasi, M. Cossi, J. M. Millam, M. Klene, C. Adamo, R. Cammi, J. W. Ochterski, R. L. Martin, K. Morokuma, O. Farkas, J. B. Foresman, and D. J. Fox. Gaussian~16 Revision C.01, 2016. Gaussian Inc. Wallingford CT.

59. Maitra, N. T. Charge transfer in time-dependent density functional theory. *J. Phys. Condens. Matter* **29,** 423001 (2017).

60. Chen, E. C. M., Shuie, L.-R., Desai D'sa, E., Batten, C. F. & Wentworth, W. E. The negative ion states of sulfur hexafluoride. *J. Chem. Phys.* **88,** 4711–4719 (1988).
