# Supplementary material for: Autocatalytic degradation of the extremely potent greenhouse gas SF6 in basic alcoholic solution
Source: Nat Commun. 2025 Dec 6;17:465. doi: 10.1038/s41467-025-67158-w (PMC12800194; doi:10.1038/s41467-025-67158-w)
Supplement: Supplementary file 2 — Description of Additional Supplementary Files [file 41467_2025_67158_MOESM2_ESM.pdf]

Description of Additional Supplementary Files:

Supplementary Video 1: Photochemical degradation of SF6 with the KOH/iPrOH system using Irradiation Setup 2. Displayed on the left side is the quartz tube containing the reaction mixture under an atmosphere of 2 bar SF6 which gets exposed to light at 280 nm emitted from an LED array next to the tube. Displayed on the right is the simultaneous uptake of SF6 during the reaction monitored by the pressure control unit (PCU).

Supplementary Video 2: Photochemical degradation of SF6 with the KOH/iPrOH system using Irradiation Setup 4. Displayed on the left side is the 1L round-bottom flask containing the reaction mixture under an atmosphere of 2 bar SF6 which gets exposed to light at 280 nm emitted from LED arrays located in a quartz tube inside of the round-bottom flask. Displayed on the right is the simultaneous uptake of SF6 during the reaction monitored by the pressure control unit (PCU).
